# Living alone with dementia is a neglected source of inequality: findings from a scoping review of research evidence

**DOI:** 10.1186/s13643-025-03002-y

**Published:** 2025-12-11

**Authors:** Linda Clare, Anthony Martyr, Maria Caulfield, Laura D. Gamble, Catherine Charlwood, Jane Ward, Claire Hulme, Matthew Prina, Jan R. Oyebode

**Affiliations:** 1 NIHR Policy Research Unit in Dementia and Neurodegeneration University of Exeter (DeNPRU Exeter), Exeter, UK; 2 Department of Health and Community Sciences, University of Exeter Medical School, Exeter, UK; 3 NIHR Applied Research Collaboration South-West Peninsula, Exeter, UK; 4 Centre for Applied Dementia Studies, University of Bradford, Bradford, UK; 5 Wolfson Centre for Applied Health Research, Bradford, UK; 6 Population Health Sciences Institute, Newcastle University, Newcastle-Upon-Tyne, UK; 7 Expert by experience, Hampshire, UK

**Keywords:** Adverse outcome, Community, Crisis, Emergency preparedness, Harm, Inclusion, Precarity, Satisfaction with life

## Abstract

**Background:**

With growing proportions of single-person households, increasing numbers of people with dementia are living alone, challenging the still-prevalent assumption that people have an informal carer available. We aimed to characterise the research literature on people living alone with dementia and summarise what is known about their characteristics and needs.

**Method:**

This scoping review followed Joanna Briggs Institute methodology and PRISMA-ScR reporting guidelines. Seven databases (PubMed, Web of Science, CINAHL, Ageline, EMBASE, PsycInfo, Social Policy and Practice) were searched for English-language publications on 18/01/2024, without date limits. Eligible studies reported on people with dementia living alone, using any research design; reviews, editorials, and conference abstracts were excluded. Titles, abstracts, and full texts were screened independently. Data were extracted using structured forms and summarised narratively, grouping quantitative findings descriptively and qualitative findings thematically.

**Results:**

We included 200 articles (162 quantitative, 38 qualitative) from 161 studies. Living alone was the primary focus in 30.5% of articles, living situation was explored in secondary comparisons or sub-group analyses in 62.5%, and noted only in describing samples in 7%. Most research (80.1%) was from Europe or North America. The first study was published in 1962 and the next in 1984, since when the annual number of publications has gradually increased. Across studies, people living alone with dementia were commonly described as older, more often female, and as experiencing significant unmet need. Reports noted variation in the extent of informal support, with some people receiving little or no support. Compared with those living with others, people living alone were often described as having less timely diagnosis, lower access to formal services, higher home care costs, and greater likelihood of moving into residential care or dying outside the home. Studies commonly reported social isolation, loneliness, and difficulties with daily living in people living alone with dementia. Family members providing support at a distance were described as receiving little assistance. Few studies examined approaches to addressing these needs or improving support.

**Conclusions:**

This review highlights living alone with dementia as a growing but neglected source of inequality. Practical steps are needed now to address this issue in policy, service provision, practice and research.

**Supplementary Information:**

The online version contains supplementary material available at 10.1186/s13643-025-03002-y.

## Background

Thirty years ago, Alzheimer’s Society in the UK issued a policy report titled ‘Dementia and Living Alone’ [1]. This emphasised the urgent need for better identification and support for people living alone with dementia, including tailored care services, improved community networks, and proactive interventions to reduce risks such as isolation, neglect, and harm, and called for coordinated efforts across health, social care, and voluntary sectors. At that time, 12% of the UK population and 33% of people aged over 65 were living alone.

Today, almost 1 in 3 UK households (8.3 million, 30%) are single person households, and this is now the second most common type of living arrangement [2]. This trend is equally evident in the USA, where more than 1 in 4 households were single person households in 2020 (36.2 million, 27.6%), up from 7.7% in 1940 [3]. Half of people aged 65 or over in the UK are living alone [2], and a significant proportion will have dementia. A recent study reported that 42% of people living in the community in Northern Ireland, UK, who were receiving dementia medication were living alone [4] while a recent US estimate suggested 25 to 30% [5].

People living alone with dementia are a group with significant unmet needs, inequitable access to services, and increased risk of adverse outcomes including crises and emergency admissions, serious harms, and social isolation [6]. They experience difficulties engaging and interacting with services and may find services unresponsive to their situation and needs. Data from the British IDEAL cohort demonstrated that, relative to those living with others, people with dementia living alone had poorer physical and mental health and lower levels of social participation, and rated their quality of life, satisfaction with life and well-being less positively, with all these differences persisting over time [6].

Within this overall picture, people living alone with dementia are a heterogenous group. Some have good support from family members living nearby, others have more limited support, perhaps because family members live at a distance, and some have little or no support, perhaps because they have no family, or have lost touch [7]. Some have the financial resources to pay for support, while others live in precarity [8]. The most vulnerable may only be identified through contact with emergency services. In the IDEAL cohort, 18% of participants with dementia recruited through memory services who were living alone had little or no support [9], while in a German study 9% of participants with dementia recruited in primary care had no informal support [10].

Nevertheless, despite the early warning in the ‘Dementia and Living Alone’ report and awareness of the continued trends towards greater numbers of older people living alone, progress in recognising and addressing the needs of people living alone with dementia appears limited. The assumption that everyone with dementia has a carer available remains widespread in policy, practice and research [8]. This needs to change if people living alone are to experience equitable access to health and social care services and community agencies that can respond to their needs as these evolve over time, reducing the risk of crises and emergency admissions and enabling people to remain at home for as long as possible if they so wish.

We set out to conduct a scoping review of the research evidence about people living alone with dementia. Our research question was: what is known about the characteristics and needs of people with dementia who are living alone and how best to support them?

## Method

The scoping review was part of a wider policy research study that covered living alone with a range of neurodegenerative conditions [11, 12]. Our methodological approach was structured according to Joanna Briggs Institute guidance for scoping reviews [13], and the review was reported in line with the Preferred Reporting Items for Systematic reviews and Meta-Analyses extension for Scoping Reviews (PRISMA-ScR) guidelines [14], as shown in the checklist included in Supplementary Table 1, Additional File 1. The protocol is publicly and freely available and details our approach and methods [15]. As appropriate for a scoping review, we did not seek to assess the quality of the evidence identified.

### Identifying relevant studies

We searched for relevant literature in seven databases, selected to cover a wide range of relevant disciplines: PubMed; Web of Science Core Collection; CINAHL ultimate and Ageline via EBSCOhost; and EMBASE, PsycInfo, and Social Policy and Practice via OVID. The search was limited to English-language publications, except in Social Policy and Practice, which does not support this restriction. No date limits were applied. The search was conducted on 18th January 2024 by one author (AM).

Search terms were:

- dement* OR Alzheimer* OR Parkinson* OR Lewy OR fronto* OR Parkinsonism OR Huntington* OR Chorea OR amyotrophic lateral sclerosis OR ALS OR motor neuron* disease OR MND OR progressive muscular atrophy OR Gehrig OR neurodegen* OR neurolog* OR cognitive impairment
- AND
- Living alone OR Live* alone OR Single-living OR One-person household OR Singlehood OR Single people OR Single person OR Single men OR Single women OR solo

The exact search strings used in each database are shown in Supplementary Table 2, Additional File 1.

### Eligibility criteria

The populations of interest were people who lived alone with a diagnosis of a relevant neurodegenerative condition, irrespective of type, severity, or age. While dementia, Parkinson’s, Huntington’s, and motor neurone disease were specific targets in the search, studies of rarer neurodegenerative conditions were also eligible. For a study to be included, at least 80% of the sample had to have a relevant condition. This was to allow for inclusion of studies with mixed samples where a sufficient proportion had the relevant condition such that the findings were likely to be meaningful in relation to that condition.

Where study participants were described as having ‘cognitive impairment’, the article was eligible if the term was being used to cover probable undiagnosed dementia, but not if it reflected a formal diagnosis of mild cognitive impairment, as many people diagnosed with mild cognitive impairment do not progress to dementia [16]. Studies where participants were described as having ‘cognitive impairment not dementia’ or ‘CIND’, or where the cognitive impairment was due to head injury, stroke, other acute insults, or other health conditions were excluded.

There were no restrictions on research design. Quantitative, qualitative, mixed method, and case studies reporting cross-sectional and/or longitudinal associations were all eligible. Where studies reported trials, baseline information was included, and outcomes were included where data were disaggregated according to living situation and compared for people living alone and with others. Reviews, editorials, letters, opinion pieces, and published conference abstracts were excluded. Published conference abstracts were used to find subsequently published articles that were not already included in the review. When articles were found via this route, they were included in the ‘Identification via other methods’ section of the PRISMA flowchart.

### Procedure

**Fig. 1:**
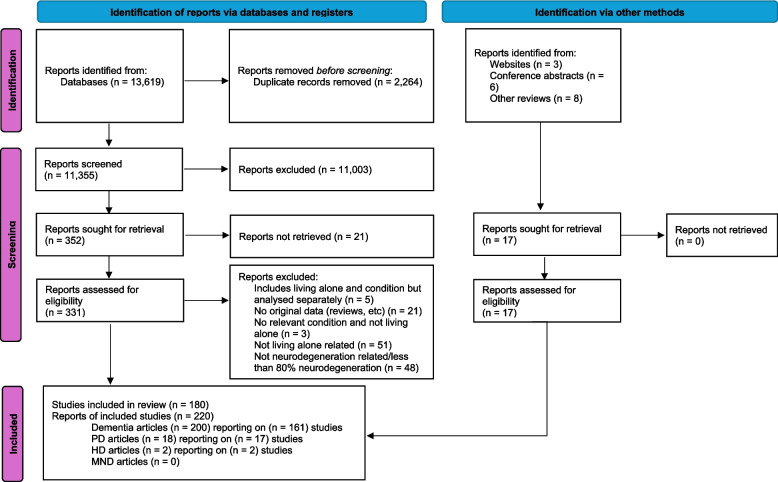
Flowchart showing the selection process for research articles PD Parkinson's, HD Huntingdon's disease, MND Motor neurone disease

The PRISMA flowchart shows the article selection and screening process; see Fig. 1. EndNote was used to manage records throughout the review. Duplicate entries were removed using the duplicate function and then checking during the screening process for any duplicate titles that were previously missed.

Titles and abstracts of all records were screened by two reviewers working independently (AM, LC). Where it was unclear whether articles should be included, these were discussed by the two reviewers. Full texts of potentially relevant articles were obtained and screened for eligibility by one researcher (quantitative: AM, qualitative: MC). To check for consistency, 20% of these titles were reviewed independently by another member of the review team (LC, LDG, JRO, MP), blinded to the decision of the first. Any discrepancies with the initial screening were discussed, and consensus was reached in all cases.

Articles using data from the same study were identified and collated to avoid duplication of reporting. Where the same variables were included across multiple articles from the same study, priority was given to the article reporting the largest sample size. Where sample sizes were identical and where the same variables were included, priority was given to the most recently published article.

Data and study characteristics from articles that met inclusion criteria following full text screening were extracted into an Excel spreadsheet (quantitative: AM, qualitative: MC). Ten percent of extracted data was checked by a second reviewer (quantitative: LDG, qualitative: JRO). For quantitative articles, details of measures and summary statistics were included. For qualitative studies, overarching themes and subthemes, representative quotations, and main findings related to living alone were extracted. Data were charted using a structured extraction form. Quantitative findings were summarised descriptively, and qualitative findings were grouped thematically to provide a narrative synthesis of trends and experiences.

### Collating, summarising and reporting results

Articles were first grouped by condition. Here we focus solely on studies relating to people living alone with dementia of any type. Characteristics of this set of research evidence were summarised and described. The articles were then grouped by topic into categories to summarise the evidence through detailing key findings. Categories were refined through an iterative process to form the structure for a narrative account covering who is living alone and why; dementia, health and access to formal care; informal support; costs of care; the experience of living alone with dementia; managing everyday life; implications for family members and practitioners; and better supporting people living alone with dementia. Evidence from quantitative studies provided an indication of trends, patterns, and associations relevant to people living alone with dementia and differences between those living alone and those living with others. Qualitative studies provided insight into personal experiences and challenges; findings from these are included here but will be reported separately in more detail.

### Consultation

As part of the wider policy research project, we involved contributors with lived experience throughout, discussed our findings with members of our stakeholder and lived experience networks, and explored perceptions of the findings in two dedicated consultation workshops, one with lived experience contributors and one with health and social care professionals. The primary purpose of the consultation was to facilitate translation of findings into meaningful policy recommendations for the English health and social care context. This is not reported here but can be found in the related policy report [12].

## Results

Full text screening covered a total of 331 reports, resulting in the inclusion of 200 peer-reviewed journal articles/reports, of which 162 were quantitative and 38 qualitative. These reported findings from 161 discrete research studies, 128 quantitative and 33 qualitative. The flowchart in Fig. 1 summarises the screening process; see Supplementary Table 3, Additional File 1 for details of the reports excluded at the full text screening stage. We identified through other sources four conference abstracts containing brief reports of two intervention studies. A list of the included articles can be found in the Supplementary Materials, Additional File 1. Details of the quantitative and qualitative studies are summarised in Supplementary Table 4, Additional File 1 and Supplementary Table 5, Additional File 1, respectively. An overview of the qualitative studies will be provided as part of the separate paper focusing on these in more detail.

### Overview of the research literature

**Fig. 2:**
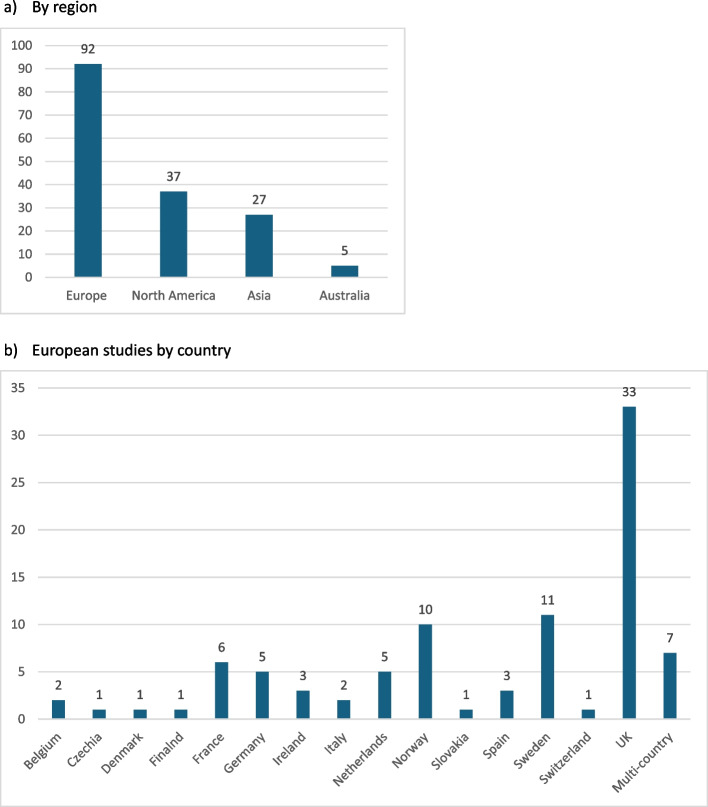
Geographical distribution of the included studies

The geographical distribution of the research is summarised in Fig. 2. Four-fifths of the studies (129; 80.1%) were conducted in or led from Europe (92; 57.1%) or North America (37; 23%).

**Fig. 3:**
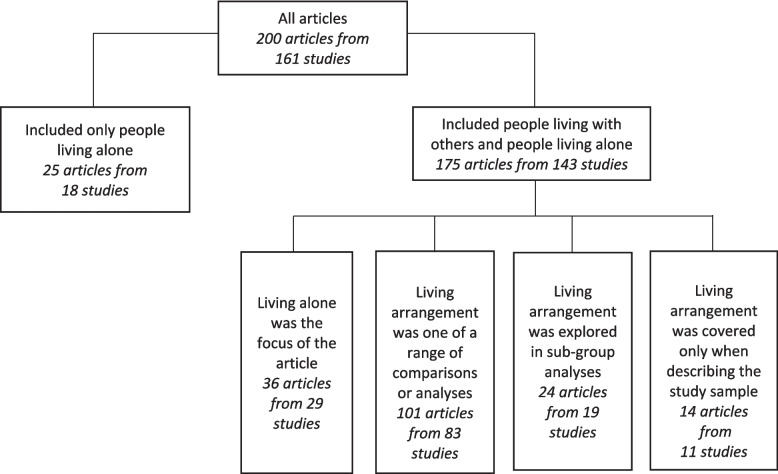
Focus of the included articles

Living alone was the primary focus of 61 (30.5%) articles, of which 25 (12.5%) recruited only people living alone. In 125 (62.5%) articles with a broader focus, comparisons according to living arrangement were included alongside other analyses or as sub-group analyses. The remaining 14 (7%) articles considered living arrangement only in terms of describing the study sample. The focus of the included articles is summarised in Fig. 3.

Across the studies which included people living alone alongside people with other living arrangements, the proportion of people living alone varied from 2.2 to 82.6%. Variability is to be expected given different approaches to sampling and participant recruitment, but even when considering only information from sources such as national registries and routine health data, considerable variability remains. Table 1 provides a summary of the proportion of people living alone with dementia recorded in studies based on clinical records, administrative health data, national registries and large surveys, and across all included studies.

**Table 1:** Proportions of people with dementia living alone by data source

**Type of data**	**Source of data**	**Total number of Plwd**	**% Plwd living alone**	**Publication date**
Clinic records	Barcelona (Spain) [17]	5792	23.4%	2017
Administrative health data	South London and Maudsley NHS Trust London [18] Dutch Primary Care Data [19]	3075 1040 3471 5423 1078	26% 21% under 70 years 33% 70–80 years 52% 80–90 years 70% 90–100 years	2016 2020
National registries	SveDem (Sweden) [20]	64,955	47.4%	2021
	Several Swedish registries [21]	43,372	62.0%	2021
Large surveys	Health and Retirement Survey (USA) [22]	4760	36.8%	2022
	English Longitudinal Study of Ageing [23]	1157†	42.4%	2023
	Survey of Health, Ageing and Retirement (EU and Israel) [23]	5166†	36.3%	2023
	China Health and Retirement Longitudinal Study [23]	2069†	10.2%	2023
Specific groups	Plwd who applied for long-term care services (Japan) [24]	23,638	18.8%	2017
	Plwd prescribed dementia medication (Northern Ireland) [4]	25,418	42.0%	2020
	Plwd not using long-term care insurance (South Korea) [25]	278,215	7.0%	2021
	Plwd 75 + who conveyed to hospital by ambulance (England) [26]	8277	23.4%	2021
	Plwd admitted to a care home (Wales) [27]	34,514	17.5%	2021
All quantitative studies included in scoping review*	Studies with sample size 500 + (32 studies) Studies with sample size < 500 (81 studies)	Range 503–7609 Range 4–468	2.3–81.5% 2.2–82.6%	

*Plwd* People living with dementia

*Excluding studies that recruited only people living alone

† This paper reported person-waves rather than number of people for prevalence of living alone

**Fig. 4:**
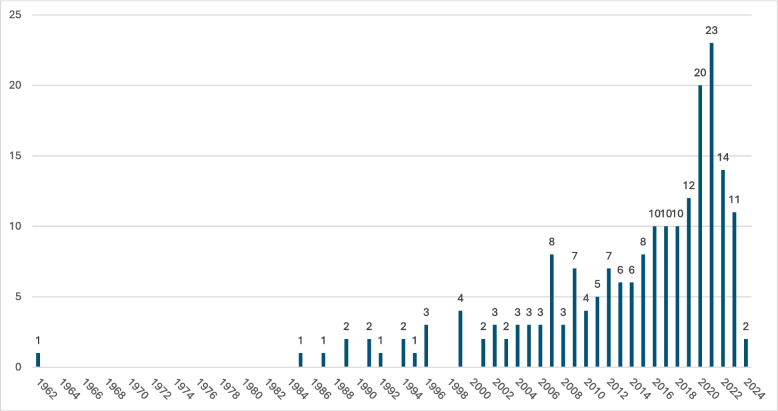
Research articles included in the scoping review by publication date

The earliest paper we identified was published in 1962, and the next appeared in 1984. Subsequent publication trends indicate a growing interest in this topic; see Fig. 4.

### Who is living alone with dementia and why

People living alone with dementia are a diverse group. The nature and extent of their family and social networks, and hence availability of informal support from family or other sources, varied widely. They may have lived alone throughout their lives or may have been widowed or divorced; for some, the onset of dementia itself may have contributed to relationship breakdown [28]. Some were in good mental and physical health while others experienced long-standing challenges in these areas, including substance abuse. Financial circumstances, suitability of housing and the potential to access local resources likewise varied considerably.

It is undisputed, however, that worldwide there are more women than men living alone with dementia. This finding is evident in all 35 studies reporting sex distribution; see Table 2. Many of these women were living alone following the death of a husband. Twenty-five of the 30 studies reported that people living alone were, on average, older than those living with others. There was little evidence about differences linked to ethnicity in the proportion of people living alone with dementia. Compared to those living with others, levels of education (12 studies) and income (5 studies) were similar or lower, and in one study people living alone were slightly more likely to live in deprived areas [4].

**Table 2:** Overview of findings related to dementia, health, and service utilisation

Topic	Articles	Summary of findings
Dementia		
Dementia severity [29–35]	7	Less in 4, similar in 3
Dementia progression over time [6, 36, 37]	3	Similar in 2, slower in 1
Cognitive ability [6, 9, 10, 29–31, 33–35, 37–47]	21*	Better in 10, similar in 9, poorer in 2
Functional ability [9, 10, 29–31, 33–35, 37, 38, 40–42, 44–50]	20	Better in 10, similar in 8, poorer in 2
Distress or neuropsychiatric symptoms [6, 30, 34, 35, 37, 38, 41, 45, 51, 52]	10†	Similar in 6, more in 3, fewer in 2
Mental and physical health		
Depression [6, 9, 10, 37, 38, 41, 44, 45, 53–56]	14	Similar in 9, more in 3
Physical health [6, 9, 32, 37, 55, 57]	6	Similar in 2, better in 2, similar or worse in 2
Co-morbid conditions [6, 31, 37, 39, 40, 42, 44, 58]	8	Similar in 6, more in 2
Mortality rates [34, 37, 40, 49, 59–61]	7	Lower in 3, similar in 2, higher in 2
Falls [10, 41, 62]	3	Similar in 1, more in 1, fewer in 1
Malnutrition or weight loss [10, 34, 37, 40, 41, 43, 44, 63–65]	10	Similar in 5, worse in 5
Medication		
AChEI and memantine prescribing [4, 9, 31, 39, 40, 49, 66]	7	Similar in 3, less in 2, mixed picture in 2
Antipsychotic prescribing [4, 39, 40, 49, 67, 68]	6	Similar in 2, more in 2, less in 2
Antidepressant prescribing [4, 39, 40, 49]	4	Similar in 2, higher in 2
Anxiolytic prescribing [4, 39, 40]	3	Similar in 3
Health care utilisation		
GP home visits [6, 9, 31, 69]	4	Similar in 1, more in 3
GP office attendance [6, 9, 10, 30, 31, 47]	7	Similar in 2, more in 1, fewer in 4
Nurse home visits [9, 31, 32, 41, 69–71]	7	Similar in 4, more in 2, fewer in 1, mixed in 1
OT or physiotherapy consultations [9, 31, 32, 41]	4	Similar in 3, fewer in 1
Outpatient appointment attendance [9, 24, 30, 32, 40, 41, 44, 72, 73]	9	Similar in 4, more in 4, fewer in 1
Emergency department attendance [9, 31, 32, 69]	4	Similar in 3, less frequent in 1
Ambulance use [26, 31]	2	Higher in 2
Hospitalisation rates [10, 18, 32, 34, 37, 40, 44, 47, 60, 69, 74]	11	Similar in 5, more frequent in 3, lower in 3
Discharge home from hospital [19, 60, 75–77]	5	Less likely in 5
Social and home care utilisation		
Social worker visits [9, 31, 32, 72]	4	Similar in 2, more in 1, fewer in 1
Use of meal delivery services [9, 10, 30, 32–34, 41, 47, 69, 71, 72, 78]	13*	More in 10, same in 3
Use of home help or cleaner [6, 9, 10, 34, 41, 47, 69, 78]	8	More in 5, same in 3, fewer in 1
Use of home care [9, 10, 19, 21, 30–34, 37, 40, 41, 45, 47, 49, 64, 69–73, 78–85]	29	Similar in 10, more in 17, less in 2
Day care use [6, 9, 10, 31–33, 41, 45, 71, 81, 83, 85]	14*	Same in 7, more in 5, less day care use in 1
Rate of admission to residential care [18, 19, 21, 27, 31, 33, 34, 37, 40, 41, 45, 49, 59, 60, 71, 81, 85–96]	28	Higher in 22, similar in 6

^*^ One article [41] included data from two separate studies so is counted twice here

^†^ One article [38] looked at agitation and number of psychiatric symptoms so is counted twice here

*AChEI* Acetylcholinesterase inhibitor, *GP* General practitioner, *OT* Occupational therapy

### Dementia, health and access to formal care

Comparisons between people living alone with dementia and those living with others provided little evidence for consistent differences in relation to dementia severity or symptoms, mental or physical health, prescribing of medication, or use of health care services; see Table 1. The evidence does, however, highlight possible differences in relation to diagnosis and subsequent care.

Four of five studies that explored timeliness of diagnosis reported that people living alone were more likely to receive a later diagnosis. Some authors suggested that symptoms of dementia may be less readily recognised by family members or GPs in people living alone [29, 38, 97]. One study found that in specialist services, people living alone received less detailed assessments, with fewer scans, less neuropsychological testing, and less precise diagnoses [39]. One study described variations in patterns of prescribing cholinesterase inhibitors and memantine between people living alone and those living with others [4].

Some studies described people living alone as having more unmet needs across multiple domains, for example relating to cognition, physical health, mobility, eyesight and hearing [30, 98]. These differences in needs point to the possibility that people living alone may experience or respond to interventions in ways that are not the same as those living with others. Three trials suggested differences in outcome for people living alone. They were more likely to improve physical performance during rehabilitation after hip fracture [57] but less likely to benefit from a cognitive training programme [99]. In the German DelpHi trial of dementia care management, 50% of participants lived alone, and while some outcomes were positive overall, quality of life improved only for those living with others [100].

Rates of hospitalisation did not appear to differ, but when people living alone went into hospital, it was a consistent finding that they were less likely to be discharged home. People living alone were more frequently described as being discharged to institutional settings, with some studies highlighting that such instances appeared to increase over time [6]. Qualitative studies indicate that many people living alone with dementia found the prospect of moving into a care home frightening or unbearable [101, 102], and institutional living may be particularly challenging for people used to living alone. People living alone were more likely to die in hospitals or long-term care settings [86, 103].

### Informal support

Informal support from unpaid, usually family, carers may be less readily available for those living alone. People living alone with dementia who received some informal support might have an identified carer, not necessarily near at hand but possibly in a different county, country or continent; often this was an adult child [40] whereas carers of those living with others were more likely to be spouses [30–32]. Those who had no identified carer often received some informal support from various sources including relatives, neighbours and friends [104]. Several studies noted limitations in support for people living alone. For example, one US study found that 64% of people living alone described their support as inadequate [72], while a UK study reported that fewer than half of those living alone had daily in-person contact with an unpaid carer [105].

Some people living alone with dementia had little or no informal support; they might have no family at all, or no relatives who were willing or able to provide care. The proportions identified in research studies ranged from 3 to 15% [9, 10, 17, 106], but these may be underestimates. As many as 10% might have no one they can count on in an emergency [10]**.**

### Costs of care

We identified 13 papers outlining nine distinct studies that examined costs of care for people with dementia who lived alone. Of the nine studies, four were from the UK [18, 33, 107–109]; the remainder were one each from South Korea [25], China [58], Japan [110], Germany [111–114] and Finland [31]. The majority were cohort studies that compared individuals with dementia or cognitive impairment either living alone or living with others, and some included care home residents. Only one paper compared the cost-effectiveness of an intervention for those living alone and those living with others [113].

While the evidence that related to costs was generally sparse, there was relatively good consensus within the available evidence across geographical locations. With respect to formal services, health and social care costs were reported as higher for those living alone than for those living with family and/or friends, but lower than for people in care homes [18, 25, 33, 107, 111, 112]. Estimates of the magnitude of the difference varied [31, 109] with numerous factors contributing. Social care packages tended to be larger or more intensive, and thus more costly, for those living alone [33]. Costs of packages were primarily driven by home care but services such as day care could also be costly drivers [31].

Where costs included the value of informal care and out of pocket costs, the picture was different. Societal costs (health and social care, out of pocket costs and unpaid care) were *lowest* for those living alone compared to those in a care home or living with others [107]. The value of informal caring provided by co-resident carers was high, and so the cost of social care packages for those living with a carer was relatively lower [33, 107]. The observation of higher home care costs for those living alone supports the notion that care for people with dementia has shifted from the social care sector to informal caring where a family member was available [31]. Living alone with dementia or cognitive impairment could lead to a high burden of catastrophic expenditure [58]; i.e., expenditure that exceeds a specific proportion of a household’s income [115]. However, the evidence was limited to a single study and based on the cost of health care to an individual and their family.

The German DelpHI trial assessed the cost-effectiveness of collaborative dementia care management (DCM) vs usual care [113] and conducted secondary analyses [116] based on living situation from the health system (public payer) perspective to assess who benefits most from DCM; 50% of participants lived alone. Overall, DCM was cost-effective compared with usual care due to a lower hospitalisation rate and an average delay in institutionalisation of 7 months, with a probability of 88% at willingness-to-pay thresholds of 40,000€ per QALY gained. When results were disaggregated the probability of DCM being cost-effective was higher in people living alone compared to those not living alone (96% vs. 26%).

### The experience of living alone with dementia

People living alone were reported as generally being at greater risk of experiencing loneliness and isolation [117, 118] and loneliness was more likely to be severe [118]. Social networks were similar in size or smaller [118], and people had less social support [10] and more unmet needs for company [30]. People living alone were less likely to participate in social and cultural activities [9, 119]. Diminishing abilities and loss of confidence resulted in fewer social interactions, and for some, the television or looking out of the window provided the only source of company [101, 120].

Accounts described a sense of lacking purpose and meaning [121–125]. Although there was no evidence of differences in quality of life ratings across 11 studies, one study found lower scores for satisfaction with life [9], especially among those with little or no support. Qualitative studies suggest that the emotional impact of losses associated with dementia may be felt more profoundly when living alone [101, 121, 122, 126, 127].

### Managing everyday life

Some studies reported that, even where levels of functional ability were described as similar across groups, people living alone had more unmet needs in relation to managing everyday activities, household tasks and self-care [30, 98]. They were more likely to buy in social care services to cover household tasks, but this was only available to those who could afford it. There may be sex-based inequities; a Swedish study noted that women living alone were less likely to receive home care than men living alone [21].

People living alone were reported to be more likely to use assistive technology than those living with others [9, 128]. Specifically, they were more likely to use safety-related products such as pendant alarms and smoke alarms whereas those living with others were more likely to have devices to track their whereabouts if they become lost [105]. User-activated social alarms that enable people living alone to call for help in an emergency were common [128], but their utility has been questioned [129].

Studies described growing difficulty with coping strategies as dementia progresses, especially for more complex tasks such as managing finances [48], and people living alone may become less willing to seek or accept help and more suspicious of the intentions of others [41, 51]. Over time, increasing difficulties were described with self-care, hygiene and nutrition [41, 49, 79], with problems due to not eating or drinking most frequently causing concern [130]. Safety at home was also highlighted, with reports of harm through their own actions, such as inadvertently starting a fire, as well as having an increased vulnerability to crime [41, 42]. In emergency situations, studies noted that people living alone were less likely to be prepared and to have evacuation plans or emergency supplies [131]. Studies also reported that people living alone went missing at similar frequency levels as those living with others [41] but that people living alone were more likely to die before being found [132, 133].

### Implications for family members and practitioners

Evaluating and managing risk is a key concern for family members involved in providing or arranging care from a distance. Family members generally supported the person’s preference to remain at home and tried to balance the perceived risks of living alone against the risks associated with institutionalisation [102, 134]. However, over time ‘red flags’ such as observations of weight loss, changes in personal hygiene, a build-up of unwashed laundry, a failure to return phone calls, or suspected exploitation or financial abuse marked the transition from acceptable to ‘unacceptable’ levels of risk. This was compounded where the person with dementia resisted measures aimed at increasing vigilance such as bringing in paid carers [102, 134, 135] and where it was difficult to find trustworthy paid carers [136].

Practitioners involved in supporting people living alone with dementia grappled with the dilemma of ‘safety and supervision versus risk and independence’ and the challenge of finding the best balance [137] in a situation where ‘few decisions are perfect’ [138]. Decision-making was especially hard where there was evidence of communication difficulties or a lack of awareness of difficulties and needs [139], or the person’s capacity was in question. Practitioners identified a range of concerns and risks [140] and found it especially challenging where it was difficult to work together with family members [125, 137]. They often felt constrained by systemic limitations such as inflexible service structures, cutbacks and resource shortages [138] which impeded their ability to support people with dementia living at home.

### Better supporting people living alone with dementia

Few of the identified studies explored ways of addressing unmet needs and better supporting people who live alone.

Two small-scale studies reported in conference abstracts described targeted intervention approaches. One was a virtual support group for 12 recently diagnosed individuals living alone with dementia, most of whom had no identified carer [119, 141]. The second was a proof-of-concept study aimed at establishing feasibility of a case management intervention, Services to Age in Your Home (STAY Home), to increase utilisation of health and social care services among some of the most vulnerable individuals living alone with dementia or cognitive impairment [142, 143].

A befriending scheme for isolated individuals with dementia was not limited to those living alone but provided three case studies illustrating the way in which facilitated friendships contributed to addressing unmet needs for social contact among people with dementia living alone [144]. A case study from China [145] described implementation of a personalised, intensive community-based social care intervention not designed specifically for people living alone to enable an isolated person living alone with dementia to reintegrate into the community and maintain functional ability.

A few small-scale studies described the use of assistive technology specifically for people living alone [146, 147]. The relatively small number of studies may reflect the need for caution as adopting and using various forms of assistive technology may require input and oversight from a carer or other supporter, or perhaps an assumption that people living alone would be unable to adapt to using assistive technology, when in fact they may be able to do so given appropriate support; it is essential to ensure that any assistive technology deployed is fit for purpose and useful, and this was not always the case [129].

Community agencies play an important role in supporting people with dementia and are potentially well-placed to understand and address the specific needs of people living alone. An early demonstration of this was a programme run by the Alzheimer’s Association of Los Angeles which focused on support for long-distance carers of a person with dementia [136].

## Discussion

This scoping review aimed to provide an overview of research activity on living alone with dementia, give a descriptive account of research findings, and identify research gaps. Inclusion of 200 articles suggests extensive research activity, and the number of publications year-by-year demonstrates a growing interest in the topic. However, less than one-third of the identified studies focused specifically on living alone. The descriptive account of research findings indicates that living alone with dementia is more common among older women and that people living alone with dementia experience disadvantage in several respects. These included descriptions of greater and more varied needs, particularly in the absence of informal support, and accounts of later diagnosis and less access to formal support. Some studies also noted that people living alone, or their families, incurred greater personal costs for care, while state costs tended to be lower overall than for people living with others. Other reports described people living alone with dementia as experiencing more loneliness and isolation, encountering greater difficulties managing everyday life, and receiving less informal support. People living alone with dementia were also described as moving into residential care earlier and as being less likely to be discharged home from hospital or to die in their own home. Evaluating and managing risk is a key task for family members providing support from a distance, along with any community-based practitioners involved. This evidence points to the relevance of considering living situation through an inequalities lens. Although the review has considered a large body of research literature, several major gaps are evident. First, there is limited consideration of inequalities and intersectionality, compounded by a general tendency to consider people living alone as one homogeneous group, whereas there are multiple pathways to living alone with dementia and a great deal of diversity. Second, evidence from lower- and middle-income countries about people living alone with dementia is lacking. Third, remarkably little attention has so far been paid to exploring ways of better supporting people living alone with dementia.

While few trials analysed outcomes by living situation, some studies described differences between those living alone and those living with others that were framed in terms of potential inequalities. Andersen’s behavioural model of health service use [148] emphasises that living with others facilitates access to formal care, and the lack of a co-resident carer is a key factor likely to limit access to care for those living alone, especially where compounded by smaller informal support networks. In the literature reviewed here, people living alone were described as having smaller informal support networks, and reports of delayed diagnosis [29, 38, 97] and more unmet needs [30, 98] were more commonly seen in this group. However, lack of a co-resident carer is not the only consideration; some studies referred to wider pre-existing structural disadvantage such as poverty, poor housing or estrangement from family, and reported that people living alone with dementia were likely to experience poorer health and more limited access to services. These social determinants [149] do not occur as isolated factors but are complex and overlapping [150]. Examples included the ways that living alone intersects with gender and age, with reports suggesting that older women living alone with dementia, sometimes facing social disadvantage, were among those in less frequent receipt of support services [151]. Class, ethnicity and sexual orientation, among other things, also combine to shape the experience of living alone with dementia and accessing or interacting with services, yet the intersection of these factors is rarely explored. Instead of considering people living alone with dementia as one homogenous group, future research could aim to better understand how equity-focused approaches to improving support can take account of the multiple intersecting aspects of situation and identity that influence the experience of living alone with dementia.

None of the included studies focused on people living alone with dementia in less well-resourced countries. Although living alone may be less common than in Western high-income countries [152], demographic shifts and changing family structures mean that it is becoming more likely in lower- and middle-income countries, where two-thirds of people living with dementia are to be found [153]. These countries are less likely to have dementia plans in place [154] and less likely to provide basic services, with the full responsibility of care falling on family members. Challenges for people living alone with dementia may be far greater in these contexts, but evidence about the proportions of people involved and their experiences and needs is lacking. Raising awareness of this issue would be a first step.

Despite the striking lack of research about better supporting people living alone with dementia uncovered by this review, the experience of service providers, practitioners and community agencies yields a valuable knowledge-base. Several resources set out key elements of good practice in supporting people living alone with dementia [7, 155–157]. Resources are available to support practitioners and guide decision-making about the ability of a person with dementia to continue living alone at home [158–161], and to advise people living alone and their families [162, 163], and virtual support groups and community initiatives have begun to develop [164, 165]. A recent US initiative demonstrated the potential value of supporting community agencies to address the needs of people living alone with dementia, awarding grants to community-based and state agencies from 2014 to 2017, of which 25 completed and reported by 2021 [166]. The grants funded implementation of evidence-based or evidence-informed initiatives with an emphasis on sustainability. The initiative highlighted some important lessons. Across all the initiatives, providers found that the needs of people living alone with dementia were more extensive and complex than initially expected, requiring more input from qualified staff, and that approaches and systems needed modification, for example allowing more time for engaging and building trust, checking in with people more frequently, and simplifying data collection. This growing understanding derived from practice and community initiatives could both stimulate and be augmented by future research focused on developing and implementing better systems and service structures to ensure equitable support for people living alone with dementia.

### Limitations

Limitations of this review include those that are common to scoping reviews; the broad focus permitted inclusion of a large body of literature, but we did not conduct quality assessments or consider the relative robustness of findings. While the evidence pointed to a focus on inequalities, the research identified was conducted almost entirely in high-income countries, predominantly in Europe and North America. Within that context, the research provided little insight into possible cultural or ethnic differences, and people living alone in the most isolated or difficult circumstances are likely to have been underrepresented in study samples. Another consideration specific to the dementia focus is that a trend towards reporting on samples of people living with ‘cognitive impairment’ resulted in the exclusion of some otherwise insightful studies [167, 168]. While sampling in this way can be seen as a pragmatic approach given that many people living with dementia are undiagnosed, ‘cognitive impairment’ may arise for a variety of reasons or simply refer to age-related cognitive changes, and may not reflect a progressive condition, and this makes it difficult to distinguish the specific issues or needs related to living alone with dementia. Finally, a further limitation of the review is that the search strategy did not incorporate controlled vocabulary terms, such as MeSH. This decision was made to prioritise comprehensiveness and maintain a consistent search methodology across all selected databases, some of which do not utilise a controlled vocabulary. While this keyword-based approach maximised the sensitivity of our search, it likely reduced its precision and increased the number of irrelevant records to screen. It is also possible that some relevant articles were missed if their titles and abstracts did not include the specific keywords.

### Implications

The review, despite these limitations, suggests some potential implications for policy, service provision, practice and research. Policy makers may focus on ensuring that robust information about numbers of people living alone is available, identifying ways to strengthen the responses of statutory services and community agencies, raising public awareness and specifying how future research can be targeted to address identified knowledge gaps. Service providers can start by acknowledging the specific support needs of people living alone with dementia, adapt service systems and pathways to ensure they are suitable and accessible for this group, and enable practitioners to provide flexible, personalised support. Navigating services to find the right provision is challenging for all, but particularly so for people living alone with dementia, and availability of a support worker who can reliably and consistently help with ensuring the right provision is in place is crucial. The challenges facing carers providing support from a distance, who are not typically well supported, must also be acknowledged and suitable options made available. Research funders must ensure that people living alone with dementia are included in research and that research is targeted to address gaps in knowledge. As this review shows, there has been relatively little research on effective ways of supporting people living alone or implementing changes in services or pathways to better meet their needs, and this would be a valuable focus for future research efforts. In summary, the ‘scientific community and policy makers need to reframe the prevailing paradigm to reflect the reality that substantial numbers of older adults with dementia live alone with limited support from unpaid caregivers’ [8] and ensure this change is reflected across the spectrum of practice and research.

## Conclusions

This scoping review highlights living alone with dementia as a neglected source of inequality. With demographic changes resulting in a significant proportion of people with dementia living alone, a trend that is likely to increase globally, it is time to pay attention to the implications of this shift. This means, in effect, that we need to start thinking differently about how to support people with dementia. Practical steps are needed now to address this issue across the spectrum of policy, service provision, practice and research.

## Supplementary Information

Additional file 1.

## Data Availability

Data sharing is not applicable to this article as no datasets were generated or analysed during the current study.
