# Supplementary material for: Living alone with dementia is a neglected source of inequality: findings from a scoping review of research evidence
Source: Syst Rev. 2025 Dec 11;15:16. doi: 10.1186/s13643-025-03002-y (PMC12817627; doi:10.1186/s13643-025-03002-y)
Supplement: Supplementary file 1 — Additional file 1. [file 13643_2025_3002_MOESM1_ESM.docx]

**Living alone with dementia is a neglected source of inequality: findings from a scoping review of research evidence**

**Supplementary Material, Additional File 1**

Linda Clare* ^1,2,3^, Anthony Martyr^1,2^, Maria Caulfield^1,4,5^, Laura D. Gamble^1,6^, Catherine Charlwood^1,2^, Jane Ward^1,7^, Claire Hulme^1,2^, Matthew Prina^1,6^, Jan R. Oyebode^1,4,5^

1. NIHR Policy Research Unit in Dementia and Neurodegeneration University of Exeter (DeNPRU Exeter), United Kingdom

2. Department of Health and Community Sciences, University of Exeter Medical School, Exeter, United Kingdom

3. NIHR Applied Research Collaboration South-West Peninsula, Exeter, United Kingdom

4. Centre for Applied Dementia Studies, University of Bradford, Bradford, United Kingdom

5. Wolfson Centre for Applied Health Research, Bradford, United Kingdom

6. Population Health Sciences Institute, Newcastle University, Newcastle-upon-Tyne, United Kingdom

7. Expert by experience

**Supplementary Table 1. Preferred Reporting Items for Systematic reviews and Meta-Analyses extension for Scoping Reviews (PRISMA-ScR) Checklist**

| **SECTION** | **ITEM** | **PRISMA-ScR CHECKLIST ITEM** | **REPORTED ON PAGE #** |
| --- | --- | --- | --- |
| **TITLE** | | | |
| Title | 1 | Identify the report as a scoping review. | 1 |
| **ABSTRACT** | | | |
| Structured summary | 2 | Provide a structured summary that includes (as applicable): background, objectives, eligibility criteria, sources of evidence, charting methods, results, and conclusions that relate to the review questions and objectives. | 2-3 |
| **INTRODUCTION** | | | |
| Rationale | 3 | Describe the rationale for the review in the context of what is already known. Explain why the review questions/objectives lend themselves to a scoping review approach. | 4-5 |
| Objectives | 4 | Provide an explicit statement of the questions and objectives being addressed with reference to their key elements (e.g., population or participants, concepts, and context) or other relevant key elements used to conceptualize the review questions and/or objectives. | 5 |
| **METHODS** | | | |
| Protocol and registration | 5 | Indicate whether a review protocol exists; state if and where it can be accessed (e.g., a Web address); and if available, provide registration information, including the registration number. | 6 |
| Eligibility criteria | 6 | Specify characteristics of the sources of evidence used as eligibility criteria (e.g., years considered, language, and publication status), and provide a rationale. | 7 |
| Information sources* | 7 | Describe all information sources in the search (e.g., databases with dates of coverage and contact with authors to identify additional sources), as well as the date the most recent search was executed. | 6 |
| Search | 8 | Present the full electronic search strategy for at least 1 database, including any limits used, such that it could be repeated. | Supplementary |
| Selection of sources of evidence† | 9 | State the process for selecting sources of evidence (i.e., screening and eligibility) included in the scoping review. | 7 |
| Data charting process‡ | 10 | Describe the methods of charting data from the included sources of evidence (e.g., calibrated forms or forms that have been tested by the team before their use, and whether data charting was done independently or in duplicate) and any processes for obtaining and confirming data from investigators. | 8 |
| Data items | 11 | List and define all variables for which data were sought and any assumptions and simplifications made. | 8 |
| Critical appraisal of individual sources of evidence§ | 12 | If done, provide a rationale for conducting a critical appraisal of included sources of evidence; describe the methods used and how this information was used in any data synthesis (if appropriate). | n/a |
| Synthesis of results | 13 | Describe the methods of handling and summarizing the data that were charted. | 9 |
| **RESULTS** | | | |
| Selection of sources of evidence | 14 | Give numbers of sources of evidence screened, assessed for eligibility, and included in the review, with reasons for exclusions at each stage, ideally using a flow diagram. | 9, figure 1 |
| Characteristics of sources of evidence | 15 | For each source of evidence, present characteristics for which data were charted and provide the citations. | Supplementary |
| Critical appraisal within sources of evidence | 16 | If done, present data on critical appraisal of included sources of evidence (see item 12). | n/a |
| Results of individual sources of evidence | 17 | For each included source of evidence, present the relevant data that were charted that relate to the review questions and objectives. | Supplementary |
| Synthesis of results | 18 | Summarize and/or present the charting results as they relate to the review questions and objectives. | 10-19 |
| **DISCUSSION** | | | |
| Summary of evidence | 19 | Summarize the main results (including an overview of concepts, themes, and types of evidence available), link to the review questions and objectives, and consider the relevance to key groups. | 19 |
| Limitations | 20 | Discuss the limitations of the scoping review process. | 21 |
| Conclusions | 21 | Provide a general interpretation of the results with respect to the review questions and objectives, as well as potential implications and/or next steps. | 19-21 |
| **FUNDING** | | | |
| Funding | 22 | Describe sources of funding for the included sources of evidence, as well as sources of funding for the scoping review. Describe the role of the funders of the scoping review. | 24 |

JBI = Joanna Briggs Institute; PRISMA-ScR = Preferred Reporting Items for Systematic reviews and Meta-Analyses extension for Scoping Reviews.

* Where *sources of evidence* (see second footnote) are compiled from, such as bibliographic databases, social media platforms, and Web sites.

† A more inclusive/heterogeneous term used to account for the different types of evidence or data sources (e.g., quantitative and/or qualitative research, expert opinion, and policy documents) that may be eligible in a scoping review as opposed to only studies. This is not to be confused with *information sources* (see first footnote).

‡ The frameworks by Arksey and O’Malley (6) and Levac and colleagues (7) and the JBI guidance (4, 5) refer to the process of data extraction in a scoping review as data charting*.*

§ The process of systematically examining research evidence to assess its validity, results, and relevance before using it to inform a decision. This term is used for items 12 and 19 instead of "risk of bias" (which is more applicable to systematic reviews of interventions) to include and acknowledge the various sources of evidence that may be used in a scoping review (e.g., quantitative and/or qualitative research, expert opinion, and policy document).

*From:* Tricco AC, Lillie E, Zarin W, O'Brien KK, Colquhoun H, Levac D, et al. PRISMA Extension for Scoping Reviews (PRISMAScR): Checklist and Explanation. Ann Intern Med. 2018;169:467–473. [doi: 10.7326/M18-0850](http://annals.org/aim/fullarticle/2700389/prisma-extension-scoping-reviews-prisma-scr-checklist-explanation).

Supplementary Table 2. Specific search strings used in applicable databases

| Database | Search string |
| --- | --- |
| PubMed | (((((((((((((((((((dement*[Title/Abstract]) OR (Alzheimer*[Title/Abstract])) OR (Parkinson*[Title/Abstract])) OR (Lewy[Title/Abstract])) OR (Fronto*[Title/Abstract])) OR (Parkinsonism[Title/Abstract])) OR (Huntington*[Title/Abstract])) OR (Chorea[Title/Abstract])) OR (amyotrophic lateral sclerosis[Title/Abstract])) OR (ALS[Title/Abstract])) OR (motor neuron* disease[Title/Abstract])) OR (MND[Title/Abstract])) OR (progressive muscular atrophy[Title/Abstract])) OR (Gehrig[Title/Abstract]) OR (neurodegen*[Title/Abstract]) OR (neurolog*[Title/Abstract]) OR (cognitive impairment[Title/Abstract]))))))) AND (((((((((Living alone[Title/Abstract]) OR Live* alone[Title/Abstract]) OR (Single*living[Title/Abstract])) OR (One-person household[Title/Abstract])) OR (Singlehood[Title/Abstract])) OR (Single people[Title/Abstract])) OR (Single person[Title/Abstract])) OR (Single men[Title/Abstract])) OR (Single women[Title/Abstract])) OR (Solo[Title/Abstract]) |
| Web of Science | (TI=(dement* OR Alzheimer* OR Parkinson* OR Lewy OR Fronto* OR Parkinsonism OR Huntington* OR Chorea OR amyotrophic lateral sclerosis OR ALS OR motor neuron* disease OR MND OR progressive muscular atrophy OR Gehrig OR neurodegen* OR neurolog* OR cognitive impairment)) AND (TI=(Living alone OR Live* alone OR Single-living OR One-person household OR Singlehood OR Single people OR Single person OR Single men OR Single women OR solo)) OR (AB=(dement* OR Alzheimer* OR Parkinson* OR Lewy OR Fronto* OR Parkinsonism OR Huntington* OR Chorea OR amyotrophic lateral sclerosis OR ALS OR motor neuron* disease OR MND OR progressive muscular atrophy OR Gehrig OR neurodegen* OR neurolog* OR cognitive impairment)) AND (AB=(Living alone OR Live* alone OR Single-living OR One-person household OR Singlehood OR Single people OR Single person OR Single men OR Single women OR solo)) |
| EBSCOhost (CINAHL and Ageline) | TI ( dementia OR Alzheimer* OR Parkinson* OR Lewy OR Fronto OR Parkinsonism OR Huntington OR Chorea OR amyotrophic lateral sclerosis OR ALS OR motor neuron disease OR MND OR progressive muscular atrophy OR Gehrig OR neurodegen* OR neurol* ) AND TI ( Living alone OR Single-living OR One-person household OR Singlehood OR Single people OR Single person OR Single men OR Single women OR solo ) OR AB ( dementia OR Alzheimer* OR Parkinson* OR Lewy OR Fronto OR Parkinsonism OR Huntington OR Chorea OR amyotrophic lateral sclerosis OR ALS OR motor neuron disease OR MND OR progressive muscular atrophy OR Gehrig OR neurodegen* OR neurol* ) AND AB ( Living alone OR Single-living OR One-person household OR Singlehood OR Single people OR Single person OR Single men OR Single women OR solo ) |
| Ovid (EMBASE, PsycInfo, and Social Policy and Practice) | ((dement* or Alzheimer* or Parkinson* or Lewy or Fronto* or Parkinsonism or Huntington* or Chorea or amyotrophic lateral sclerosis or ALS or motor neuron* disease or MND or progressive muscular atrophy or Gehrig or neurodegen* or neurolog* or cognitive impairment) and (Living alone or Live* alone or Single-living or One-person household or Singlehood or Single people or Single person or Single men or Single women or solo)).ti,ab. |

Supplementary Table 3: Articles excluded at the full-text screening stage

|  | n |  |
| --- | --- | --- |
| Includes living alone and condition but analysed separately | 5 | Abrams, 2002[1], Barker & Himchak, 2006 [2], Bloch, 2017 [3], Boynton, 2014 [4], McArthur, 2022 [5] |
| No original data (reviews, etc) | 21 | Andrews, 2013 [6], Barry & Hughes, 2021 [7], Gilmour, 2004 [8], Griffith & Channon, 2005 [9], Keady, 1994 [10], Kikuchi, 2022 [11], Kikuchi, 2023 [12], Kolanowski, 2018 [13], Medical Ethics Advisor, 2018 [14], Miyamae, 2022 [15], National LGBT Partnership, 2015 [16], Newhouse, 2001 [17], Portacolone, 2018 [18], Portacolone, 2019 [19], Portacolone, 2022 [20], Regal & Heatherington, 2012 [21], Robinson, 2017 [22], Soniat, 2004 [23], Starns, 2002 [24], Tierney, 1997 [25], Webb, 2010 [26] |
| No relevant condition and not living alone | 3 | Casaccia, 2020 [27], Flatt, 2021 [28], Wiese, 2018 [29] |
| Not living alone related | 51 | Bourennane, 2013 [30], Campbell, 2016 [31], Cañabate, 2015 [32], Chou, 2019 [33], Chu, 1991 [34], Cloutier, 2017 [35], Cunningham, 2022 [36], de Medeiros, 2022 [37], Dean, 2015 [38], Dickins, 2018 [39], Domínguez-Berjón, 2015 [40], Doron, 2017 [41], Doughty, 1998 [42], Evans, 2020 [43], Fæo, 2020 [44], Fredriksen-Goldsen, 2019 [45], Ganzini, 1999 [46], Gold, 1995 [47], Goldfarb, 2022 [48], González, 2017 [49], Gordon, 1998 [50], Gwyther, 1997 [51], Harrison, 2022 [52], Hildick Smith, 1983 [53], Hobson, 2019 [54], Hossain, 2022 [55], Hughes, 2000 [56], James, 1994 [57], King, 2017 [58], Lancioni, 2012 [59], Lebert, 2005 [60], Macleod, 2016 [61], Maunsell, 2020 [62], McGoldrick, 2021 [63], Meuleners, 2017 [64], Naharci, 2023 [65], Nwabuobi, 2019 [66], Ohta, 2018 [67], Pentecost, 2022 [68], Pfalzer, 2021 [69], Pizzi, 2022 [70], Rahja, 2018 [71], Read, 2018 [72], Read, 2021 [73], Rees, 2023 [74], Risi, 2016 [75], Savitch, 2015 [76], Tsunawaki, 2023 [77], Walker, 2011 [78], Watts, 1989 [79], Zafeiridi, 2023 [80] |
| Not neurodegeneration related/less than 80% neurodegeneration | 48 | Barry, 2019 [81], Cambridge, 2006 [82], Chen, 2020 [83], Chen, 2023 [84], Dura-Perez, 2022 [85], Edwards, 2020 [86], Fæo, 2019 [87], Feng, 2016 [88], Field, 2021 [89], Gill, 1995 [90], Goodman-Casanova, 2020 [91], Green-Harris, 2016 [92], Guzman-Parra, 2020 [93], Hagiwara, 2022 [94], Hashimoto, 2020 [95], Ishii, 2016 [96], Ito, 2022 [97], Jarrett, 2013 [98], Jurkeviciute, 2020 [99], Kadoya, 2021 [100], Kim, 2001 [101], Kirk, 2017 [102], Kullberg, 2008 [103], Larsson, 2004 [104], Livingston, 1997 [105], Lu, 2020 [106], Luppa, 2011 [107], Machesney, 2014 [108], Muurling, 2023 [109], Newman, 1995 [110], Nordloh, 2011 [111], Oxford Brookes University Institute of Public Care, 2010 [112], Portacolone, 2018 [113], Portacolone, 2019 [114], Portacolone, 2021 [115], Robertson, 2020 [116], Rockwood, 2011 [117], Schiedermayer, 1982 [118], Schwartz, 2013 [119], Senesi, 2020 [120], Sibley, 2002 [121], Tan, 2017 [122], Taylor, 2023 [123], Tsuda, 2022 [124], Walker, 1998 [125], Wrigley, 1992 [126], Xu, 2022 [127], Yang, 2022 [128] |

Supplementary Table 4. Summary of included quantitative studies

| **Study** | **Country of study** | **n** | **N living alone** | **Mean age** | **Females** | **Severity** | **Dementia type** | **Living situation** |
| --- | --- | --- | --- | --- | --- | --- | --- | --- |
| Abel, 2020[129] | Germany | 127 | alone 81 (63.8%) vs with others or assisted living | 84.7 (6.5) | f(105) | MMSE 22.8 (2.6) | Cognitive impairment | Community, hospital |
| Amjad, 2016[130] | United States of America | 7609 | alone: 33.4% (147*) probable dementia with diagnosis, 32.2% (194*) probable dementia with no diagnosis, 35.8% (357*) possible dementia |  |  | AD8 Dementia Screening Interview scores | Dementia | Community or care home |
| Amjad, 2018[131] | United States of America | 585 | Alone. Undiagnosed: 31.4% of 231 (73*), Diagnosed but unaware: 45.8% of 112 (51*), Diagnosed and aware: 33.4% of 242 (81*) |  |  | AD8 Dementia Screening Interview scores | Dementia | Community or care home |
| Okoye, 2023[132] | United States of America | 488 | alone: 115 (20.6%), spouse: 179 (45.3%), other: 194 (34.1%) | 65-74: 60 (22.4%), 75-84: 189 (41.3%), 85+: 239 (36.3%) | f(297, 54.5%) |  | Dementia | Community |
| Andrieu, 2016[133] | France | 574 | alone: 156 (32.6%), spouse: 282, other: 63 | 79.6 (5.9) | 331, 66.6% | MMSE: 19.5 (3.9) | AD | Community |
| Nourhashemi, 2008[134] | France | 1120 | intervention. alone: 182 (32.4%), spouse: 300 (53.4%), other: 80 (14.2%). Control: alone: 164 (29.4%), spouse: 290 (52.0%), other: 104 (18.6%). | 79.61 (5.72) | intervention 375, 66.7%, Control: 395, 70.8% | MMSE: 19.73 (4.01) | AD | Community |
| Soto, 2015[135] | France | 1131 | alone: 348 (30.8%), others: 783 (69.2%), senior housing: 26 (7.5%) | alone: 81.1 (5.2), others: 79.0 (5.8) | alone: f(314, 90.2%), others: f(463, 59.1%) | MMSE. alone: 19.8 (3.9), others: 19.7 (4.1) | AD | Community |
| Angel, 2024[136] | HEPESE = United States of America, MHAS = Mexico | 1623 | HEPESE=208 (28.0%) MHAS=214 (13.2%). Not all of these had dementia | HEPESE 84.0 (2.2); MHAS 81.3 (2.3) | HEPESE 65% MHAS 57% |  | Dementia | Community |
| Cantu, 2022[137] | HEPESE = United States of America, MHAS = Mexico | 115 Mexicans, 288 Mexican Americans | alone HEPESE=25 (22%), MHAS=59 (21%), live with others HEPESE= 0 (78%), MHAS=226 (79%). | HEPESE=91.63. MHAS 90.02 | Alone 9294 others 6917 |  | Probable dementia | Community |
| Rote, 2021[138] | United States of America | 2880, dementia 34% | alone: 21%, spouse: 33.2%, others: 45.8%. Note these are for the full sample and not just those with dementia | 73.6, 65-108 | 57.7% |  | Probable dementia | Community |
| Balouch, 2019[139] | England | 93 | alone: 31 (33.7%), others: 61 (66.3%) | 82.61 (6.27) | 51, 55.4% | MMSE: 20.41 (3.66), 12-26 | AD | Community |
| Bostrom, 2007[140] | Sweden | 34 | alone: 19, others: 15 | 77.4 (64-87) | 19 | MMSE: 17.3 (0-29) | DLB | Community |
| Brown, 2019[141] | England | 201 | live alone=46, live with others=63, lives in care=92 |  | 4053 |  | Dementia | Community or care home |
| Cañabate, 2017[142] | Spain | 5792 | alone=1355 (23.4%), Couple=2473 (42.7%), adult child=655 (11.3%), nursing home or professional carer=319 (5.5%), other=990 (17.1%) | 79.9 (7.7) | f(4053, 70%), m(1739, 30%) | MMSE >20=3011, MMSE 10-20=2491, MMSE <10=290 | AD=4102, VaD=770, DLB=439, FTD=280, other=201 | Community or care home |
| Carter, 2021[143] | Ireland | 42 | alone: 24% (10*), spouse: 57% (24*) | 82.5, 74-88 | f(24*, 57%), m(18, 43%) | mild=52%, moderate=38%, severe=10% | Dementia | Community |
| Keogh, 2018[144] | Ireland | 297 | alone: 84 (28.5%), spouse/partner: 155 (52.7%), adult child: 43 (14.6%), other family: 7 (2.3%), other: 5 (1.7%) | 80.6 (8.9) 51-101 | f(179, 60.5%), m(117, 39.5%) | Barthel score: 6.6 (4.7). brain imaging dependency level. maximum: 137 (46.9%), high: 94 (32.2%), medium: 51 (17.5%), low: 8 (2.7%), independent: 2 (0.7%) | Dementia | Community: 123 (41.8%), acute hospital: 145 (49.3%) or community hospitals: 21 (7.1%), nursing home: 3 (1%), psychiatric hospital: 2 (0.7%) |
| O'Brien, 2019[145] | Ireland | 428: dementia: 347. CI: 81 | alone: 221 (51.6%) | 84.2 (7.2) | f(295, 68.9%) | Barthel: 12.6 (4.0) | Dementia or suspected cognitive impairment | Community |
| Čermáková, 2017[146] | Sweden | 26123 | alone=11878 (46%), with others=14245 | alone=81.2 (7.2), others=77.0 (7.6) | Institutionalized m(168; 34.4) and Non-Institutionalized m(11; 36.7) |  | AD (n=16477) or mixed (n=9646) | Community |
| Haaksma, 2020[147] | Sweden | 55578 | 21419 (42.8%), others=23849 (47.6%), in care 4808 (9.6%) | 81.6 (76.5-86.0) | 29737, 59.4% | median MMSE=21 (17-27) | AD=15945 (31.%), mixed=10226 (20.4%), VaD=10040 (20.0%), DLB=1079 (2.2%), PDD= 41 (1.5%), FTD=601 (1.2%), other=1146 (2.3%), unspecified=10298 (20.6%) | Community and care home |
| Lexomboon, 2021[148] | Sweden | 10444 | alone=4677 (44.8%), others=4930 (47.2%), nursing home=816 (7.8%), missing=21 (0.2%) | <70=1077 (10.3%), 70-79=3468 (33.2%), 80-89=5041 (48.3%), 90+=858 (8.2%) | 6293, 60.3% | MMSE 0-9=238 (2.3%), 10-18=2314 (22.2%), 19-23=3492 (33.4%), 24-30=4136 (39.6%), missing 264 (2.5%) | AD=3174 (30.4%), mixed=3622 (34.7%), VaD=2035 (19.5%), DLB=236 (2.3%), FTD=153 (1.5%), PDD=176 (1.7%), other=1036 (9.9%), missing=12 (0.1%) | Community, nursing home |
| Schwertner, 2021a[149] | Sweden | 65717 | alone: 30809 (46.9%*), others: 26912 (41.0%*), nursing home: 7234 (11.0%*), missing: 762 (1.2%*) | <74: 12648 (19.3%*), 74-78: 13002 (19.8%*), 79-84: 16566 (25.2%*), 85+: 23386 (35.6%*), missing: 115 (0.2%*) | 38789, 59.0% | MMSE: 24-30: 20374 (31.0%*), 19-23: 22905 (34.9%*), 11-18: 15179 (23.1%*), 0-10: 2164 (3.3%*), missing: 5095 (7.8%*) | AD=20152 (30.7%*), FTD=987 (1.5%*), DLB=2316 3.5%*), mixed=12050 (18.3%*), VaD=12377 (18.8%*), Other=1693 (2.6%*), unspecified=16142 (24.6%*) | Community, care |
| Schwertner, 2021b[150] | As above (2021a) | As above (2021a) | As above (2021a) | As above (2021a) | As above (2021a) | As above (2021a) | As above (2021a) | As above (2021a) |
| Charles, 2015[151] | Canada | 224 | 224 | harm: 84.4 (5.6) | harm: f(19; 82.6%) | harm: 23.3 (2.4) | Cognitive impairment | Community, hospital |
| Chen, 2022[152] | Taiwan | 518 | 36 | Institutionalized (<70=3 (10%), 70-80=10 (33.3%), >80=1 (36.7%)) and Non-Institutionalized (<70=79 (16.2%), 70-80=185 (37.9%), >80=224 (45.9%)) | f(4053), m 1739) | Institutionalized (CDR-SOB=4.5; 2-7) and Non-Institutionalized (CDR-SOB=6.0; 5-8.75) | Non-AD n=229 | Institutionalized (n=30) and Non-Institutionalized (n=488) |
| Chi, 1995[153] | England | 144 (46 cognitively impaired, 49 physically frail, 49 physically healthy) | 14=cognitively impaired, 25=physically frail, 24=physically healthy | Cognitively impaired (75-79=12, 80-84=14, 85+=20), physically frail (75-79=20, 80-84=24, 85+= 5). physically healthy (75-79=30, 80-84= 14, 85+=5) | Cognitively impaired: f(21), m(25), physically frail: f(15), m(34). physically healthy: f(14), m(35) |  | Cognitive impairment | Community |
| Clare, 2020[154] | England, Scotland, Wales | 1541 | 285 (18.5%)=alone, 1256 (81.5%)=others. 51 (3%) had little or no support | alone=79.88 (9.15), others=75.57 (8.19) | alone=f(193, 67.7%), m(92, 32.3%) others=f(480, 38.2%), m(776, 61.8%) | MMSE: alone=23.59 (3.33), others=23.15 (3.68) | alone: AD=157 (55.1%), VaD=32 (11.2%), mixed=80 (28.1%), FTD=4 (1.4%), PDD=1 (0.4%), DLB=8 (2.8%), other/ unspecified=3 (1.1%), others: AD=697 (55.5%), VaD=138 (11.0%), mixed=246 (19.6%), FTD=50 (4.0%), PDD=43 (3.4%), DLB=45 (3.6%), other/ unspecified=37 (2.9%) | Community |

| Clare, 2024[155] | England, Scotland, Wales | 1525 | 281 (18.4%*)=alone, 1244 (81.6%*)=others. | Time 1: alone=79.97 (9.10), others=75.59 (8.16)  Time 2: alone=80.65 (9.39), others=76.46 (7.95)  Time 3: alone=80.79 (9.71), others=76.88 (7.99) | Time 1: alone=f(191, 68.0%), m(90, 32.0%) others=f(473, 38.0%), m(771, 62.0%),  Time 2: alone=f(130, 65.0%), m(70, 35.0%), others=f(372, 38.5%), m(593, 61.5%),  Time 3: alone=f(97, 67.4%), m(47, 32.6%), others=f(271, 38.9%), m(425, 61.1%) | Time 1: alone=23.56 (3.29), others=23.11 (3.67)  Time 2: alone=22.69 (4.25), others=21.35 (5.19)  Time 3: alone=21.39 (5.84), others=20.32 (6.30) | Time 1: alone AD=153 (54.4%), VaD=32 (11.4%), mixed=80 (28.5%), FTD=4 (1.4%), PDD=1 (0.4%), DLB=8 (2.8%), other=3 (1.1%), others AD=692 (55.6%), VaD=135 (10.9%), mixed=243 (19.5%), FTD=50 (4.0%), PDD=43 (3.5%), DLB=45 (3.6%), other=36 (2.9%)  Time 2: alone AD=107 (53.5%), VaD=22 (11.0%), mixed=59 (29.5%), FTD=3 (1.5%), PDD=1 (0.5%), DLB=5 (2.5%), other=3 (1.5%), others AD=543 (56.3%), VaD=92 (9.5%), mixed=204 (21.1%), FTD=36 (3.7%), PDD=33 (3.4%), DLB=34 (3.5%), other=23 (2.4%). Time 3: alone AD=77 (53.5%), VaD=16 (11.1%), mixed=44 (30.6%), FTD=2 (1.4%), PDD=1 (0.7%), DLB=3 (2.1%), other=1 (0.7%), others AD=406 (58.3%), VaD=65 (9.3%), mixed=140 (20.1%), FTD=29 (4.2%), PDD=16 (2.3%), DLB=24 (3.4%), other=16 (2.3%) | Community |
| --- | --- | --- | --- | --- | --- | --- | --- | --- |
| Henderson, 2019[156] | England, Scotland, Wales | 1547 | 306 (20%) | <65=136 (9%), 65-69=178 (12%), 70-74=260 (17%), 75-79=370 (24%), 80+=603 (39%) | f(675, 44%), m(872, 56%) | Participants with no carer had mean baseline MMSE 1.07 points higher than those with carers (24.12; 95% CI, 23.74–24.50 vs. 23.05; 95% CI, 22.83–23.25) | AD=858 (55%), VaD=171 (11%), mixed=326 (21%), FTD=54 (3%), PDD=44 (3%), DLB=53 (3%), other/unspecified=41 (3%), | Community |

| Henderson, 2022[157] | England, Scotland, Wales | Time 1: 1537,  Time 2: 1199,  Time 3: 910 | Time 1: 285 (18.6%), Time 2: 208 (17.7%), Time 3: 141 (16.6%). Imputed data (M= 40); N= 1175 at Time 2 and N= 846 at Time 3 | Time 1: 76.4 (8.5), Time 2: 76.1 (8.4), Time 3: 75.5 (8.5) | f(675, 44%), m(872, 56%) | MMSE: Time 1=23.2 (3.6), Time 2=21.6 (5.1), Time 3=20.5 (6.2) | Time 1: AD=851 (55.4%), VaD=170 (11.1%), mixed=324 (21.1%), FTD=54 (3.5%), PDD=44 (2.9%), DLB=53 (2.7%), other/ unspecified=41 (2.7%), | Community |
| --- | --- | --- | --- | --- | --- | --- | --- | --- |
| Sabatini, 2023[158] | England, Scotland, Wales | 1182 | alone: 132 (11.2%), spouse: 987 (83.4%), other: 61 (5.2%) | 76.10 (8.17) | f(484,. 41.0%), m(698, 59.1%) | alone MMSE=23.59 (3.33), n=278. others MMSE=23.15 (3.68), n=1192. | AD=659 (55.8%) VaD=122 (10.3%), mixed=249 (21.1%), FTD=45 (3.8%), PDD=39 (3.3%), DLB=38 (3.2%), other=30 (2.5%) | Community |
| Victor, 2020[159] | England, Scotland, Wales | 1547 | alone: 285 (18.5%), others: 1256 (81.5%) | <65: 136 (8.8%), 65-69: 178 (11.5%), 70-74: 260 (16.8%), 75-79: 369 (23.9%), 80+: 604 (39.0%) | f(676, 43.7%), m(871, 56.3%) | 23.2 (3.6), missing n=71 | AD=858 (55.5%), VaD=171 (11.1%), mixed=326 (21.1%), FTD=54 (3.5%), PDD=44 (2.8%), DLB=53 (3.4%), other/ unspecified=41 (2.7%) | Community |
| Conde-Sala, 2009[160] | Spain | 236 | alone: 34 (14.4%), spouse: 120 (50.8%), other family: 39 (16.5%) | 77.8 (6.9), 55-93 | f(157, 66.5%) | MMSE. mild (21+): 76 (32.2%), moderate (11-20): 154 (65.3%), severe (0-10): 6 (2.5%) | AD | Community |
| Curnow, 2021[161] | England | 451 | alone=203, live with spouse=181, live with others=67 |  | f(264) | alone MMSE=18.8, spouse=17.6, other=17.6 | Dementia | Community |
| Curran, 1996[162] | Scotland | 19 | alone=9. responders:(6), non-responders: (3) | responders: 80.4 (73-86), non-responders: 78.5 (67-90) | responders: f(8; 100%), non-responders: f(3), m(8) | responders: MMSE=19 (8-25), non-responders: MMSE=17.3 (12-28) | Dementia | Not stated, likely community |
| Dartigues, 2023[163] | France | Pre-plan (baseline)=108, post plan (T10 years)=115 | Pre-plan=31 (29.0%), post plan=42 (36.5%). Living in care: Pre-plan=18 (16.7%), post plan=30(26.1%). | Pre-plan=82.9 (5.2), post plan=86.6 (5.6). | Pre-plan=f(44, 40.7%), post plan=f(47, 40.9%). | MMSE: Pre-plan=17.7 (6.9), post plan=18.4 (5.0). MMSE groups: Pre-plan 0-9=13 (12.4%), 10-17=35 (33.3%), 18+=57 (54.3%), post plan 0-9=6 (6.5%), 10-17=24(25.8%), 18+=63 (67.7%) | Dementia | Community and residential care |
| Diesfeldt, 1992[164] | Netherlands | 224 | alone: 87 (47.3%), spouse: 97 (52.7%). This is after 1 year. No baseline living alone data available | female: 79.3 (7.0). Male: 77.7 (8.0) | f(133), m(91) | moderate dementia 76.2%, severe dementia 23.8% | Dementia, 95.1%, amnestic syndrome 8, recurrent major depression 2, hypochondriasis 1 | Community 66.1% (148), residential homes 33.9% (76) |

| Dramé, 2012[165] | France | 425 | 42% | 86 (6) | 63% | MMSE=15 (7) | Dementia | Geriatric ward |
| --- | --- | --- | --- | --- | --- | --- | --- | --- |
| Durand, 2009[166] | England | 50 (plus their carers) | 50 | 80.92 (6.32); 62-92 | f(42, 84%) | MMSE=21.64 (3.42); 12-30 | Dementia | Living in a private residence 36 (72%), in a sheltered flat 14 (28%) |
| Ebly, 1999[167] | Canada | 317 at baseline, follow-up 242 (63 died, 2 refused, 10 lost to follow-up) | alone=100 (31.5%) | alone: 82.9 (5.5), not alone 81.7 (6.2) | alone: f(77%), not alone f(53.9%) | 3MS score: alone: 56.6 (13.1), not alone 52.4 (16.2) | Alone: Probable and possible AD: 75%, VaD: 13%, other: 12%. Not alone: Probable and possible AD: 67.3%, VaD: 19.8%, other: 12.9%. | Community and institution, though numbers for these are unclear. Everyone living alone lived in the community |
| Tuokko, 1999, study 1[168] | Canada | 177 | alone: 70 (39.55%*), others: 107 (60.45%*) | alone: 83.20, married: 80.34, widowed: 84.84 |  |  | AD | Community |
| Edwards, 1996[169] | United States of America | 65 | alone=37, others=28 | alone=75.6 (9.2), others=79.8 (7.6) | alone: f(35, 95%) m(2, 5%), not alone f(18, 64%) m(10, 36%) | alone=CDR0.5=10, CDR1=14, CDR2=12, CDR3=1; others=CDR0.5=8, CDR1=10, CDR2=7, CDR3=3 | Dementia | Community |
| Edwards, 2007[170] | United States of America | 343 | alone=179 | alone=81.3 (9.3), others=80.4 (7.4) | alone: f(87%) m(13%), not alone f(67%) m(33%) | MMSE alone=18.38 (6.9), others=15.05 (7.3). alone*= DR0.5=65 (36%), CDR1=95 (53%), CDR2=18 (10%), CDR3=2 (1%); others=CDR0.5=30 (18%), CDR1=71 (43%), CDR2=54 (33%), CDR3=8 (5%) | AD | Community |
| Eichler, 2016[171] | Germany | 511 | alone=260 (50.9%), others=251 | alone=81.5 (5.6), others=79.1 (5.2) | alone: f(186, 71.5%), not alone f(117, 46.6%) | MMSE=alone 22.2 (5.0), n=233, others 22.2 (5.6), n=235 | Dementia | Community |
| Michalowsky, 2016[172] | Germany | 262 | alone: 142 (54.2%) | 80.69 (5.4); 70-100 | f(148, 56.5%) | MMSE mean 21.66 (5.0), 6-30 | Dementia | Community |
| Michalowsky, 2018[173] | Germany | 425 | alone: 207 (48.7%) | 80.2 (5.3); 70-100 | f(240, 56.5%) | MMSE mean 22.8 (4.9), 5-30 | Dementia | Community |
| Michalowsky, 2019[174] | Germany | 444 | alone: intervention: 163 (51.8%), control: 61 (47.3%) | intervention: 80.7 (5.7), control: 79.7 (4.9) | intervention: f(190, 60.3%), control: f(77, 59.7%) | MMSE mean: intervention: 22.2 (5.1), control: 21.8 (5.8) | Dementia | Community |

| Rädke, 2020[175] | Germany | 444 | alone: intervention: 163 (51.8%), control: 61 (47.3%) | intervention: <80: 177 (56.2%), 80+: 138 (43.8%), control: : <80: 67 (51.9%), 80+: 62 (48.1%) | intervention: f(190, 60.3%), m(125, 39.7%), control: f(77, 59.7%), m(52, 40.3%) | intervention: 27-30: 107 (34.0%), 20-26: 110 (35.0%), 0-19: 92 (29.2%): control: : 27-30: 51 (39.5%), 20-26: 48 (37.2%), 0-19: 29 (22.5%) | Dementia | Community |
| --- | --- | --- | --- | --- | --- | --- | --- | --- |
| Thyrian, 2017[176] | Germany | 407 | alone: 204 (50.1%) | Intervention: 80.6 (5.7), control: 79.8 (5.0) | f(248, 60.9%) | MMSE. Intervention: 22.8 (4.6), control: 22.7 (5.2) | Formal dementia diagnosis: 156 | Community |
| Ennis, 2014[177] | United States of America | 382 PwD (total n=10.431) | n=118 (2.9% of 4100 who lived alone, 264 lived with others, 4.2% of 6331) |  |  |  | Dementia | Community |
| Farsi, 2022[178] | Italy, Netherlands, Switzerland, and Norway | 32 | 32 |  |  |  | Dementia | Community |
| Ferretti, 2010[179] | Switzerland | all=1764, PwD=425 | PwD living alone=57.6% (245*), people without dementia living alone=61.1% (818*) | all PwD=84.4 (6.2) | all PwD=66.1% (281*) | all PwD MMSE=19.1 (5.5) | AD=260, mixed=41, VaD=24, DLB=15, other=85 | Hospital ward |
| Fisk, 1987[180] | United States of America | all=1028, PwD=267, AD only=159 | AD living alone=47% (75*) | all AD=77 | all AD=73% (116*) | AD MMSE=15.3, n=113 | AD | Community |
| Fowler-Davis, 2020[181] | England | n=30 dyads | n=30 | female: 78-96, male: 65-92 | f(23), m(7) |  | Dementia | Community |
| Fritze, 2011[182] | Norway | 224 (199 with depression data, 25 without) | 35.6% (71*) with depression data, 45.5% (11*) without | 75.1 (7.8) with depression data, 79.2 (7.4) without | male 41.7% (83*) with depression data, male 45.8% (11*) without |  | AD=138, DLB=56, PDD=11, VaD=11, FTD=4, alcohol-related dementia=3 | Community |
| Fukatsu & Kanemoto, 2022[183] | Japan | 16 with PBS from 511 PwD | living alone=10 (62.5%) | 79.9 (4.4);72-87 | f(15), m(1) | MMSE=18.2 (7.3); 2-29 | DLB=7, AD=3, delusional disorder=3, unspecified dementia=2, VaD=1 | Not stated, likely community |
| Gage, 2015[184] | England | 109 | alone=63, care home=14, lives with carer=28, lives with others=4 | alone=85.9 (5.48);69-97, care home=88.4 (6.18); 75-99, lives with carer=83.5 (6.15); 73-98, lives with others=81.7 (9.03); 74-91 | alone=m (13, 20.6%), care home=m (5, 35.7%), lives with carer=m (8, 28.6%), lives with others=m (1, 25%) | MMSE: alone=13.93 (4.87), n=3, care home=8.0 (4.36), n=40, lives with care=13.38 (5.22), n=13, lives with others=13.0 (0.0), n=1 | Dementia | Community and care home |
| Gallucci, 2016[185] | Italy | 90 | alone=19 (21.11%), live with one relative=44 (48.88%), live with assistance=6 (6.66%), live with family=18 (20%), live in care=3 (3.33%) | 77.8 (6.7) | f(73, 81.11%), m(17, 18.88%) | MMSE=20.4 (3.4) | AD and AD with cerebrovascular disease | Community and care home |
| Gibson, 2014[186] | United States of America | 710 | 710 | 65-69=31 (4.4%), 70-74=70 (9.9%), 75-79=103 (14.5%), 80-84=157 (22.1%), 85-89=177 (24.9%), 90+=172 (24.2%) | m(215, 30.3%), f(495, 69.7%) |  | Cognitive impairment | Not stated, likely community |
| Gibson, 2017[187] | United States of America | 710 | 710 | 65-69=31 (4.4%), 70-74=70 (9.9%), 75-79=103 (14.5%), 80-84=157 (22.1%), 85-89=177 (24.9%), 90+=172 (24.2%) | m(215, 30.3%), f(495, 69.7%) |  | Cognitive impairment | Not stated, likely community |
| Giebel, 2021[188] | Wales | 34514 | 5896 (17.5%), others 27891 (82.5%) | 84 (7) | f(23586, 68.3%), m(10928, 31.7%) |  | AD=15534 (45.0%), VaD=12633 (36.6%), FTD=270 (0.8%), DLB=487 (1.4%) |  |
| Gregory, 1996[189] | England | 49 | 49 | 60-70=3, 70-80=27, 80+=19 |  |  | dementia | Community |
| Hamers, 2016[190] | Netherlands | 827 | 409 ((50%) | 81.6 (6.7) | f(497, 60%) | Cognitive Performance Scale mean 2.9 (1.3) | 76% dementia, no description of the others other than cognitive impairment | Community |
| Han, 2021[191] | South Korea | 457524 | 19526 (4.3%) | 95.1% over 65 | f(71.2%) |  | Dementia | Community and hospitals (living alone were community) |
| Hansen, 2011[192] | Denmark | 268 | 83 (31.0%) | 76.1 (6.8) | f(155, 57.8%) |  | AD | Community |
| Harsányiová & Prokop, 2018[193] | Slovakia | 428 | 80 (19%) | 77.4 (4.47). | f(269, 63%) | baseline: Alone=24.4 (3.34), with family=25.0 (2.43), nursing home=25.4 (3.26). 12 months: Alone= 14.2 (6.91), with family=21.1 (4.11), nursing home=19.2 (6.67) | VaD=217, (51%), AD=211 (49%) | Community and care home |
| Hattori, 2022[194] | Japan | 711 (cognitively impaired=353, not-cognitively impaired=358, 50.4%) | 256 (36.1%) (cognitively impaired=128 (36.4%), not-cognitively impaired=128, 35.9%) | 82.0 (4.8) (cognitively impaired=82.9 (4.9), not-cognitively impaired=81.2, (4.5) | Cognitively impaired: f(211, 59.8%), m(142, 40.2%). Not-cognitively impaired: f(231, 64.5%), m(127, 35.5%). |  | Cognitive impairment | Community |
| Herrick, 2016[195] | United States of America | 2320 | alone=854 (35%), spouse=1013 (46%), others=453 (19%) | 78 | f(1539, 65%), m(781, 35%) | no impairment=1278 (57%), mild=625 (27%), mod to severe=200 (9%), diagnosis of cognitive impairment=217 (8%) | Cognitive impairment | Community |
| Houttekier, 2014[196] | Belgium | 1409 | 208 (14.9%), LTC 706 (50.7%), multi-person house (478 (34.3%) | <65=14 (1.0%), 65-74=86 (6.1%), 75-84=566 (40.2%), 85+=743 (52.7%) | f(763, 54.23%), m(646, 45.8%) |  | AD=477 (33.9%), VaD=117 (8.3%), unspecified=815 (57.8%) | Community and care home |
| Hsieh, 2020[197] | Taiwan, South Korea | 503 from Taiwan, 77 from South Korea | 45 (9.0%) from Taiwan, 19 (24.7%) from South Korea | 77.6 (7.8) Taiwan, 77.2 (5.7) South Korea | f(349, 69.4%; Taiwan), f(58, 75.3%; South Korea) | Taiwan: MMSE baseline=16.9 (6.1), 1-yr=16.4 (6.4), 2-yr=14.6 (7.3), South Korea: MMSE baseline=17.0 (5.4), 1-yr=15.9 (6.1), 2-yr=14.6 (6.1), | Dementia |  |
| Iwasaki, 2021[198] | Japan | 1051 | alone=24 | alone=79.1 (4.9) | alone=f(16, 66.7%%), m(8, 33.3%) | alone=22 (19-23) | Cognitive impairment | Community |
| Janssen, 2020[199] | Netherlands | 11012 | 45-70=21% of 1040 (218*), 70-80=33% of 3471 (1145*), 80-90=52% of 5423 (2812*), 90-100=70% of 1078 (755*) | 79.8 (8.0) | f(61%) (n=6717*) |  | Dementia | Community |
| Ju, 2019[200] | South Korea | 2304 | intervention group=80 (22.7%), control group 1=185 (54.9%), control group 2=378 (23.4%) | intervention group=70-79: n=137 (38.9%), 80-89: n=194 (55.1%), 90+: n=21 (6.0%), control group 1=70-79: n=145 (43.0%), 80-89: n=175 (52.0%), 90+: n=17 (5.0%), control group 2=70-79: n=596 (36.9), 80-89: n=881 (54.6%), 90+: n=138 (8.5%) | intervention group=f(282, 80.1%), m(70, 19.9%), control group 1=f(256, 76.0%), m(81, 24.0%), control group 2=f(1288, 79.7%), m(327, 20.3%) | very mild and mild dementia using either CDR or GDS. No scores provided as data were restricted | Dementia | Community and facilities |

| Kahle-Wrobleski, 2017[201] | France, Germany, United Kingdom | 971 | alone: dependence non-progressors: 16.52% of 563 (n=93*) and dependence progressors: 12.50% of 408 (n=51*) | dependence non-progressors: 77.11 (7.69) and dependence progressors: 76.49 (7.55) | dependence non-progressors: f(55.24%) (n=311*) and dependence progressors: f(53.19%) (n=217*) | MMSE: dependence non-progressors: 18.46 (5.97) and dependence progressors: 18.14 (5.74) | AD | Not stated, likely community |
| --- | --- | --- | --- | --- | --- | --- | --- | --- |
| Kamimura, 2019[202] | Japan | 4 | 2 | alone: 78 $ 81, others 79 & 71 | Alone 2 (100%), others 1 (50%) | MMSE: Alone 21 & 23, Others 30 & 21. CDR all 0.5 | AD | Community |
| Kerpershoek, 2020[203] | Netherlands, Wales, Germany, Norway, Sweden, Ireland, Portugal, Italy | 451 | 127 (28%) | 77.8 (7.9; 47-92 | f(244, 54%), m(207, 46%) | MMSE=19 (4.9), CDR-SoB 7.1 (2-16) | AD=218 (48%), VaD=52 (11%), mixed=56 (12%), DLB=6 (1%), other/unknown=117 (27%) | Not stated, likely community |
| Kikuchi, 2019[204] | Japan | 160 | Alone=8 (5%), others=146 (91.3%), nursing or hospital care=6 (3.8%) | <65=4 (2.5%), 65-69=8 (5.0%), 70-74=22 (13.8%), 75-79=41 (25.6%), 80-84=45 (28.1%), 85-89=32 (20.0%), 90-94=7 (4.4%), 95+=1 (0.6%) | f(79, 49.4%), m(81, 50.6%) | FAST1=6 (3.8%), FAST2=20 (12.5%), FAST3=13 (8.1%), FAST4=14 (8.8%), FAST5=26 (16.3), FAST6=65 (40.6%), FAST7=16 (10.0%) | Dementia | Community and care home |
| Kikuchi, 2023[205] | Japan | 88 | 88 | 65-69=4 (4.4%), 70-74=10 (11.4%), 75-79=20 (22.7%), 80-84=31 (35.2%), 85+=23=26.1%) | f(50, 56.8%), m(38, 43.2%) |  | Dementia | Not stated, likely community |
| Kisvetrová, 2019[206] | Czechia | 282 | alone: 100 (35.5%), others: 182 (64.5%) | 80.0 (7.8) | f(177, 62.8%), m(105, 37.2%) |  | Dementia | Community |
| Kisvetrová, 2021[207] | Czechia | 279 PwD, 284 controls | alone PwD:101 (36.2%), controls: 90 (31.7%). With others PwD: 178 (63.8%), controls: 194 (68.3%) | PwD: 80.9 (7.5; 62-98. controls: 74.3 (6.8); 64-94 | PwD: f(175, 62.7%), m(104, 37.3%). Controls: f(194, 68.3%), m(90, 31.7%) |  | Dementia | Community |
| Kitamura, 2018[208] | Japan | 58 | alone:=39 (67.2%*), live with 1 working adult child=19 (32.8%*) | Preceding home-visit nursing: 77.7 (4.9), n=12, no preceding home-visit nursing: 84.1 (6.2), n=46 | Preceding home-visit nursing: 10 (83.3%), no preceding home-visit nursing: 31 (67.4%) | Preceding home-visit nursing: 16.8 (7.2), no preceding home-visit nursing: 11.8 (7.3) | Preceding home-visit nursing: AD=7 (58.3%), DLB=2 (16.7%), other=3 (25.0%). No preceding home-visit nursing: AD=25 (54.4%), DLB=13 (28.3%), other=8 (17.4%) | Time of study: acute psychogeriatric ward |
| Knapp, 2016[209] | England | 3075 | alone: 798 (26.0) | 40-59=43 (1.4%), 60-69=207 (6.7%), 70-79=1049 (34.1%), 80-89=1486 (48.3%), 90+=289 (9.4%) | f(2059, 67.0%), m(1013, 33.0%) | MMSE: 0-10=323 (10.5%), 11-20=1391 (45.2%), 21-30=1361 (44.3%) | AD | Community |
| Lam, 1989[210] | England | 200 (126 dementia) | PwD only. hospital: 22% (n=18*), homes: 57% (26*) | PwD only. hospital: 80.0 (9.7), homes: 84.3 (6.4) | PwD only. hospital: f(50), m(31), homes: f(35), m(10) | PwD only, % with self-care difficulties. hospital: 54%, homes: 84% | Hospital: 84% (n=81), homes: 43% (45) | PwD only: 81 Islington hospital ward, 45 Islington old people homes |
| Lampinen, 2022[211] | Sweden | 1176 | alone: 883 (76.0%). PwD: 284, without dementia: 599 | 90.6 (4.72) | f(70.9%, 244) | MMSE, 16.3 (5.0) | Dementia | Community, nursing home (PwD only: n=190, all=324) |
| Lehmann, 2010[212] | United States of America | 349 | alone: 97 (27.8%), others: 252 (72.2%). Spouse 64, adult child/in-law 150, sibling 11, non-relative 27 | 81.8 (7.1) | f(284, 81.4%) | MMSE. alone: 20.7 (5.5), others: 15.6 (7.3) |  | Community |
| Lin (H.R.), 2017[213] | Japan | 77159: PwD=23638 (30.6%), without=53521 (69.4%) | PwD: 9612 (18.0%). Without: 4433 (18.8%), p<0.05 | PwD: 84 (7.14), without=83.26 (7.79) | PwD: f(16619, 70.3%), m(7019, 29.7). Without: f(36656, 68.5%), m(16865, 31.5%) |  | Dementia | Not stated, likely both community and in care |
| Lin (Z.), 2023[214] | United States of America, England, European Union + Israel, China | HRS: 2750, ELSA: 1157, SHARE: 5166, CHARLS: 2069 | HRS: 804 (29.2%), ELSA: 491 (42.4%), SHARE: 1876 (36.3%), CHARLS: 212 (10.2%) | HRS: 75.0 (11.8), ELSA: 75.6 (10.8), SHARE: 78.7 (9.4), CHARLS: 68.8 (9.1) | HRS: f(1751, 62.6%), ELSA: f(645, 55.7%), SHARE: f(3076, 59.5%), CHARLS: f(1170, 56.5%) | number of I/ADL limitations: HRS: 3.9 (2.9);n=2698, ELSA: 3.3 (2.5);n=1157, SHARE: 3.7 (2.9);n=5166, CHARLS: 3.3 (2.6);n=2068 | Dementia | Community |
| Li, 2022[215] | China | 38341** | 909 | alone: 71.9 (9.5), not alone: 58.1 (8.6) | alone: f(623, 68.5), m(286 (31.5%), not alone: f(17632, 47.1%), m(19800, 52.9%) |  | Cognitive impairment | Not stated, likely community |
| White, 2022[216] | United States of America | 4760 | alone: 1754 (36.9%*) | <65=719 (15.1%*), 65-74=934 (19.6%*), 75-84=1688 (35.5%*), 85+=1419 (29.8%*) | m(1851, 38.9%*) |  | Dementia | Not stated, likely community and care |

| Zuo & Heflin, 2023[217] | United States of America | 3531 | alone: 30% (n=1059*) | 60-69: 20.8% (735*), 70-79: 33.8% (1194*), 80+: 45.3% (1600*) | f(65.2%, 2302*) |  | Dementia | Community |
| --- | --- | --- | --- | --- | --- | --- | --- | --- |
| Lofthouse-Jones, 2021[218] | England | 110781 (PwD: 18288, without: 92493) | PwD alone: 887 (60.7%)=1461*, PwD alone with care package: 1056 (49.4%)=2138* | PwD 75-79: 1417 (60.3%)=2350*, 80-84: 2587 (59.2%)=4370*, 85-89: 3561 (60.0%)=5935*, 90+: 3231 (57.3%)=5639* | PwD f(6645, 58.2%=11418*), m(4119, 60.5%=6808*) |  | Dementia | Community, care |
| LoGiudice, 2001[219] | Australia | PwD=353 (English speakers: 273, non-English speakers: 81) | PwD English speakers=92 (35.7%), PwD non-English speakers=14 (20.6%) | PwD English speakers=76.2 (7.9), PwD non-English speakers=71.2 (8.3) | PwD English speakers=f(178, 65.2%), PwD non-English speakers=f(46, 56.8%) | MMSE: PwD English speakers=18.0 (5.3); n=262, PwD non-English speakers=14.7 (6.2); n=67 | PwD English speakers: AD=165 (62.7%), VaD=33 (12.5%), other=65 (24.6%), PwD non-English speakers: AD=54 (66.7%), VaD=8 (9.9%), other=19 (23.5%%) | Community |
| Manivannan, 2022[220] | United States of America | 97 | n=22 (23%) | 74.5 (15.6) | f(58; 60%) | mild=45, moderate=38, advanced=14 | Dementia | Community |
| Måvall & Malmberg, 2007[221] | Sweden | 51 | 22 | men: 79, women: 80.3 | f(30), m(21) |  | Dementia=39 (76%), remaining 12 had memory problems, and 11 of these had executive dysfunction | Community |
| Meaney, 2005[222] | Ireland | 82 | 22 (26.8%*) | 76 (7.8) | f(55), m(27) | MMSE mean 15.9 | Dementia | Community |
| Mengelers, 2022[223] | Netherlands, Belgium | 844: Netherlands (n=627), Belgium (n=217) | 349 (41.4%) (Netherlands (n=292, 46.6%), Belgium (n=57, 26.3%) | 82.0 (6.7) Netherlands (81.5, 6.8), Belgium (83.4, 6.4) | f(507, 60.1%); : Netherlands f(366, 58.4%), Belgium f(141, 65.0%) |  | Dementia | Community |
| Mets, 2013[224] | Belgium | 719 | alone: 234 (32.5%), spouse: 385 (53.5%), other family: 58 (8.1%), other: 42 (5.8%) | 79.1 (6.4) | f(453, 62.9%) | MMSE mean 20.5 (4.3) | AD | Community |
| Michelet, 2020[225] | Norway | 226 | with others 40.2% of 222 (89*). Alone: 133* | 81.3 (6.7) | f(135*, 59.7%) | MMSE median 22.0 | Subjective cognitive impairment/not dementia 3.5% (8*), MCI 10.6% (24*), dementia 85.8% (194*) | Community |
| Miranda-Castillo, 2010[226] | England | 152 | alone: 50 (32.9%*), others 102 | alone: 81.7 (5.9), others: 78.0 (6.9) | alone: f(38, 76.0), others: f(40, 82.4) | MMSE: alone: 19.9 (6.2), others: 18.8 (7.7) | Dementia | Community |
| Miyawaki, 2023[227] | United States of America | 25 | alone: 12 (48.0%), spouse: 3 (12.0%), other: 10 (40.0%) | 81.0 (9.0) | f(21, 84.0%), m(4, 16.0%) |  | Dementia | Community (17, 68%), care (8, 32%) |
| Mjørud, 2020[228] | Norway | 2938 | alone: 1208 (41.1*), others: 1622 (55.2%*) | women: 76.6 (8.6), men: 75.2 (8.4) | f(1679, 57.1%*), m(1259, 42.9%*) | MMSE: women: 76.6 (8.6), men: 75.2 (8.4) | AD=1549, VaD=269, mixed=433, DLB/PDD=227, other=460 | Community at baseline |
| Moholt, 2020[229] | Norway | 430 | alone: 68.8% others: 31.2% | 82.59 (6.86) | f(63.5%), m(36.5%) |  | Dementia or cognitive impairment consistent with dementia | Community (82.6%), care (17.4%) |
| Montastruc, 2013[230] | France | 684 | alone: 180 (26.3%), spouse: 403 (58.9%), family: 80 (11.7%), other: 21 (3.2%) | 77.9 (6.8) | f(486, 71.1%), m(198) | MMSE: 20.00 (4.2) | AD | Community |
| Nourhashemi, 2005[231] | France | 677 | alone: 186 (27.5%*), others: 491 (72.5%*) | alone: 79.4 (5.9), others: 76.9 (7.0) | alone: m(12*, 6.46%), others: m(188*, 38.28%) | alone: 19.8 (4.0), others: 20.1 (4.2) | AD | Community |
| Tavassoli, 2013[232] | France | 686 | alone: 182 (26.5%), others: 492 (71.7%), home for elderly: 12 (1.8%) | 77.9 (6.8) | f(488, 71.1%) | MMSE: 20.0 (4.2) | AD | Community |
| Morris, 1962[233] | England | 100, 62 dementia | alone: 32. dementia alone=21* | 73 (5.9); 66-88 | f(100, 100%) |  | Dementia=62, depression=30, paraphrenia=7 | Not stated likely community |
| Morycz, 1985[234] | United States of America | 80 | alone: 7*, 21%, family: 52, 65% | 78 | f(62, 77%) | MMSE: 17 | AD | Community |
| Murayama, 2009[235] | Japan | 56 | alone: 12, any family: 44 | delusion group: 77.2 (6.1), non-delusion group: 78.6 (4.9) | f(39), m(17) | delusion group: 17.7 (2.8), non-delusion group: 18.2 (2.7) | AD | Community |
| Nakanishi, 2020[236] | Japan | 553 | alone: 75 (13.6%) | 80.3 (7.3); 57-100 | f(402, 72.7%) | mild=156, moderate=209, moderately severe/severe=188 | AD | Community |
| Nguyen, 2024[237] | Vietnam | 90 | alone: 2 (2.2%), spouse: 51 (56.7%), adult child: 31 (34.4%), other family: 6 (6.7%) | 75.8 (10.0) | f(52, 57.8%), m(38, 42.2%) | CDR0.5: 8 (8.9%), CDR1: 19 (21.1%), CDR2: 27 (30.0%), CDR3: 36 (40.0%) | Dementia | Community |
| Nicholas, 2021[238] | United States of America | dementia: 27302, not dementia: 54062 | dementia: 27302, not dementia: 54062 | dementia: 79.4 (7.52), not dementia: 74.8 (7.3) | dementia: m(8586, 31.4%), not dementia: m(18196, 33.7%) |  | AD and related dementias | Not stated, likely community |
| Nygård, 2008[239] | Sweden | 788 | alone: 642 (81.5%), cohabiting: 125 (15.9%), missing, 21 (2.7%) | 83.5, 43-104 | f(591, 75.0%), m(197, 25.0%) |  | Dementia=83 (10.5%), memory and cognitive deficits=565 (71.7%), memory and physical deficits=140 (17.8%) | Community |
| O'Connor, 1989[240] | England | 160 | alone: 70, 90: others (spouses=61, siblings=5, cousin=1, adult children=20, daughter-in-law=1, granddaughter=1, housekeeper=1) | 83.8, 75-97 |  | mild: 82, moderate: 64, severe: 14 | Dementia | Community |
| O'Connor, 1991[241] | England | 159 | alone: 71, with others: 88 | mild: trial 85.0; 75-97, control: 83.5, 77-94. mod or severe: trial 82.5, 75-101, control: 84.0, 75-92 | f(106) | mild: 80, moderate: 66, severe: 12 | Dementia | Community |
| Okamura, 2022[242] | Japan | 198 | alone: 57 (38%), others 95 (63%) | 80.9 (5.8) | f(91, 59%), m(64, 41%) | CDR0: 60 (39%), CDR0.5: 49 (32%), CDR1: 35 (23%), CDR2: 7 (5%), CDR3: 4 (3%) | Dementia=57 (37%), no dementia=98 (63%) | Community |
| Pentzek, 2009[243] | Germany | dementia=111 |  |  |  | mild=78, moderate=27, severe=6 | AD=60, VaD=27, mixed=14, other=5, unspecified=5 | Community |
| Pitkala, 2021[244] | Finland | 604 | alone: 106, others: 508 | alone: 83 (5), with others: 78 (6) | alone: f(87, 82%), with others: f(195, 38%) | MMSE: alone: 20.6 (3.8), with others: 17.9 (6.5) | Dementia |  |
| Pongan, 2017[245] | France | 140 | alone: 68 (48.6%)*, in relationship: 72 (51.4%)* | discharge home: 79.23 (7.09), discharge to nursing home: 80.02 (7.92) | discharge home: f(54, 62.8%, m(32, 37.2%), discharge to nursing home: f(42, 77.8%, m(12, 22.2%) | CDR1: home, 12 (16.2%), care, 3 (5.6%), CDR2: home, 38 (51.4%), care, 40 (74.1%), CDR3: home, 24 (32.4%), care, 11 (20.4%) | Dementia | Hospital, then community, care |
| Prescop, 1999[246] | United States of America | 109 | alone: 38 (34.9%*), others: 71 (65.1%*) | alone: 82.6 (3.8), others: 81.0 (5.0) | alone: m(9, 24%), others: m(40, 56%) | CDR0.5: alone, 25 (66%), others, 40 (56%), CDR1: alone, 11 (29%), others, 21 (30%), CDR2+: alone, 2 (5%), others, 10 (14%) | Dementia | Community |
| Puaschitz, 2021[247] | Norway | 276 | alone: 136 (49.3%), spouse: 135 (48.9%), adult child: 5 (1.8%) | 82.1 (7.0) | f(173, 62.7%), m(103, 37.3%) | MMSE: 20.7 (3.8) | AD=101 (36.6%), VaD=11 (4.0%), DLB=3 (1.1%), FTD=1 (0.4%), mixed/unspecified=152 (55.1%), other=8 (2.9%) | Community (264, 95.7%), residential home (9, 3.3%), other (3, 1.1%) |
| Puaschitz, 2023[248] | Norway | 82 | alone: 37 (50%), spouse: 37 (50%) | 82.9 (6.9) | m(29, 35.4%) | MMSE: 17.9 (5.0) | AD=37 (45.1%), VaD=2 (2.4%), unspecified=41 (50%), other=2 (2.4%) | Community |
| Regan, 2005[249] | England | 224 | 53 (23.77%) | 80 (55-98) | f(160, 71.4%) | mild (MMSE 21+): 66 (29.5%), moderate (MMSE 10-20): 92 (41.1%), severe (MMSE 0-9): 66 (29.5% | AD | Community, care |
| Rhee, 2011[250] | United States of America | 307 | alone: 37.8% (116*), spouse: 12.1% (37*), adult child: 13.5% (42*), other: 36.6% (112*) | 84.4 (6.9) | f(68.3%) | CDR0.5 or 1: 60.7% (187*), CDR2: 36.3% (112*), CDR3+: 3.0% (9*) | AD=67.5% (208*), VaD=20.8% (64*), other=11.4% (35*) | Community |
| Risvoll, 2017[251] | Norway | 151 | alone: 48 (31.8%) | 73.3 (10.4) | f(95, 62.9%) | 19.6 (5.8) | Dementia | Community |
| Rokstad, 2018[252] | Norway | 257 | alone: 135 (52.5%) | 81.5 (6.4) | f(168, 65.4%) | MMSE: 20.4 (3.5) | AD=202 (78.6%), VaD=22 (8.6%), mixed=14 (5.4%), DLB/PDD=13 (5.1%), FTD=2 (0.8%), other=4 (1.6%) | Community baseline |
| Rongve, 2014[253] | Norway | 189 | alone: 73 (38.6%*), others: 113 (59.8%*), missing=3 | 75.5 (7.7) | f(104), m(85) | MMSE: 24.0 (2.3) | AD=122 (64.6%), DLB=43 (22.8%), other=24 (12.7%) (PDD=9, VaD=9, FTD=3, alcohol dementia=3) | Community baseline |
| Rowe, 2012[254] | United States of America | 156 | not stated | 80.4 (6.6) | m(72.4%) |  | Dementia | Community |
| Rozzini, 2006[255] | Italy | 214 | alone: 31 (14.5%) | 80.2 (6.9) | f(143, 67%) | MMSE: 12.2 (7.4) | AD or mixed=137, VaD=28, DLB=15, FTD=16, other=18 | Hospital |
| Salva, 2009[256] | Spain | 946 | alone: 30 (3.2%), spouse: 425 (44.9%), carer: 229 (24.2%), other family: 228 (24.1%), other: 34 (3.6%) | 79.0 (7.3) | f(644, 68.1%), m(302, 31.9%) | MMSE: 15.4 (6.2) | AD=608 (64.3%), VaD=119 (12.6%), mixed=131 (13.8%), other=88 (9.3%) | Community |
| Sandberg, 2019[257] | Sweden | 131; cognitively impaired=43 (33%), not cognitively impaired=88 (67%) | alone: cognitively impaired=34 (79.1%), not cognitively impaired=76 (86.4%) | cognitively impaired: 84.9 (6.8), not cognitively impaired: 85.5 (6.6) | cognitively impaired: f(30, 69.8%), m(13, 30.2%), not cognitively impaired: f(71, 80.7%), m(17, 19.3%) |  | Cognitive impairment | Community |
| Sandberg, 2018[258] | As above | As above | As above | As above | As above |  | As above | As above |
| Schneider, 2002[259] | England | 132 | alone: 37, others: 64, residential care: 31 | 80.81 (7.71), 59-97 | f(84), m(48) | GDS: residential care: 4.42, alone: 3.72 | Dementia | Community, care |
| Shinagawa, 2012[260] | Japan | 271: city: 99, rural: 172 | city: alone=22* (22%), rural: alone=24* (14%) | city: 76.16 (8.26), rural: 74.70 (9.37) | city: f(57), m(42), rural: f(104), m(68) | MMSE. city: 21.96 (5.16), rural: 8.96 (6.23) | City. AD=68, VaD=11, DLB=7, FTD=2, other=11. Rural. AD=94, VaD=15, DLB=21, FTD=15, other=27 | Community |

| Sm-Rahman, 2021[261] | Sweden | total: 2004409, dementia: 43370 | PwD: alone 26881, without dementia alone: 906783 | male PwD: cohabiting: 79.9 (6.8), alone: 80.9 (7.7), female PwD: cohabiting: 79.1 (6.8), alone: 88.4 (7.2) | PwD alone: f(19067), m(7814), PwD not alone: f(7033), m(9458) |  | Dementia | Community, care |
| --- | --- | --- | --- | --- | --- | --- | --- | --- |
| Sm-Rahman, 2023[262] | Sweden | PwD: 1821 (29%), without: 4473 (71%) | alone at time of death (PwD): 74.4%, others: 25.6%. Without: alone: 67.3%, others: 32.7% | age at death. PwD: 86.9 (6.5), without: 83.6 (8.0) | PwD: f(60.1%, 1094*), m(39.9%, 727*), without: f(50.2%, 2245*), m(49.8%, 2228*) |  | Dementia | Community, care |
| Souza, 2016[263] | United States of America | 136 | alone: 40 (29%). others: 96 (71%) | 75 (9.25) | f(63, 46%), m(73, 54%) | MMSE: 23 (4.49 | Dementia | Community; Assisted living (4, 2%), private home (113, 84%), retirement community (14, 10%), senior housing (5, 4%) |
| Swanwick, 1999[264] | Ireland | 209 | alone: female (35%, 54*), male (11%, 6*) | 74 (6.4), 54-92 | f(153), m(56) |  | AD=129, VaD=19, mixed=671 | community |
| Thoits, 2020[265] | United States of America | 581 | alone: 130. spouse: 328, other: 123 | neuropsychological evaluation: 75.6 (8.0), n=196, multidisciplinary neurocognitive clinic: 80.4 (6.4), n=385 | m(241, 41.5%*) | mild (MoCA 20-26): 282 (48.5%*), moderate (MoCA 13-19): 203 (34.9%*), severe or profound (MoCA 0-12): 93 (16%*) | AD=412 (70.9%*), VaD=111 (19.1%*), mixed=58 (10.0%*) | Not stated, likely community or mostly community |
| Thiruchselvam, 2012[266] | Canada | 339 | 339 | no non-adherence: 83.77 (6.58), n=280, non-adherence: 84.28 (6.03), n=59 | f(268), m(71) | mDRS. no non-adherence: 120.29 (8.44), n=280, non-adherence: 119.05 (7.12), n=59 | Cognitive impairment | Community |
| Tierney, 2001[267] | Canada | 139 | 139 | 82.9 (6.9) | f(97), m(42) | MMSE: 22.7 (3.6), mDRS: 116.3 (9.5) | 54 had a dementia diagnosis | Community |
| Tierney, 2004[268] | Canada | 139 | 139 | 83.0 (7.0) | f(99), m(40) | MMSE. Harm: 21.4 (3.8), n=30, no harm: 23.4 (3.4), n=109 | Cognitive impairment | Community |
| Tierney, 2007[269] | Canada | 130 | 130 | 82.95 (6.81) | f(92), m(38) | MMSE. Harm: 23.05 (3.69), n=27, no harm: 23.92 (3.37), n=103 | Cognitive impairment | Community |
| Tochimoto, 2015[270] | Japan | 391 | alone: 69 (18%), spouse: 66 (17%), other: 256 (65%) | 81.6 (6.9) | m(160 (41%) | MMSE: 11.3 (7.7) | AD=135 (60%), DLB=78 (20%), VaD=38 (10%), other=40 (10%) | Community living before hospital |
| Tsai, 2022[271] | Taiwan | 1268 | alone: 375 (29.6%*) | No LTC use: 78.82 (8.09), Use LTC: 79.47 (7.73) | No LTC use: m(410, 39.7%); 32.3%* of full sample, Use LTC: m(89, 37.7%); 7.0%* of full sample | MMSE. No LTC use: 17.45 (6.45), Use LTC: 15.61 (6.16) | AD=895 (70.6%*), VaD=153 (12.1%*), DLB/PDD=97 (7.7%*), FTD=20 (1.6%*), other/unknown=103 (8.1%*) | Not stated, likely community |
| Tuokko, 1999, study 2[168] | Canada | 152 | alone: 82 (53.95%*), spouses: 70 (46.05%*) | alone: 80.85 (6.84), spouse: 75.01 (6.10) |  | MMSE. alone: 19.10 (5.14), spouse: 18.88 (6.25) | Dementia | Community |
| Ura, 2021[272] | Japan | baseline: 198, follow-up: 126 | alone. baseline: 90 (45.5%*), alone. follow-up: 55 (43.7%) | baseline: 83.1 (6.6) follow-up: 80.1 (5.2) | baseline: m(80, 40.4%*), follow-up: f(77, 69.4%), m(49, 38.9%) | MMSE. follow-up: 20.5 (2.8) | Cognitive impairment | Community |
| Villars, 2013[273] | France | 390 | living alone with anosognosia: 12.37% (48) | 81.79 (7.37) | f(59.97%, 230) | MMSE: 12.34 (7.19) | AD=83.88% (326*), other dementia: 11.17% (43*), psychiatric: 5.45% (21*) | Care |
| Vislapuu, 2021[274] | Norway | 105 | alone: 47 (45.6%) | 81.8 (6.9) | f(64, 61%) | MMSE: 20.8 (3.7) | Dementia | Community |
| Wang (J), 2023[275] | United States of America | 889 | alone: 157 (17.7%), others: 423 (47.6%), congregate facilities: 309 (34.8%) | 84.0 (7.94) | f(545, 61.3%) |  | Dementia | Community, care |
| Wang (W-F), 2014[276] | Taiwan | 691 | alone: 42 (6.1%), spouse/child: 530 (76.9%), friend/other family: 76 (11.0%), group (e.g., veterans home, monastery): 36 (5.2%), nursing home/foreign carer: 5 (0.7%) | 79.3 (7.7) | f(446, 64.5%) | MMSE: 15.7 (6.5), n=673 | AD | Community, care |
| Washida, 2021[277] | Japan | not stated, data are for 251 hospitals with median stroke patients per year for each=281. 31% had dementia | alone: 23.7%, family: 20.9%, institutionalization: 50.7% |  |  |  | Poststroke dementia | Community, care |
| Watari & Gatz, 2004[278] | United States of America | 272 | alone: 86, spouse only: 63, spouse and others: 23, family: 93, others: 7 | Korean Americans: 76.14 (10.04), African Americans: 77.98 (8.20), Hispanic or Latino/a: 74.97 (9.64), White: 78.72 (8.94) | f(180), m(92) | MMSE. Korean Americans: 16.03 (7.62), African Americans: 13.56 (7.35), Hispanic or Latino/a: 16.35 (7.95), White: 18.89 (7.99) | AD/dementia | Community |
| Watari & Gatz, 2006[279] | United States of America | 277: 187 local carers, 90 long distance carers | alone: 38 (14.0%*), spouse: 119 (43.9%*), adult child: 18 (6.6%*), paid carers: 31 (11.4%*), facility: 53 (19.6%*), other family: 12 (4.4%*) | local: 77.8 (8.6), long distance carers: 78.8 (8.0) | f(176, 66.2%*), m(90, 33.8%*) |  | Dementia | Community |
| Wattmo, 2011[280] | Sweden | 880. nursing home: 206, not nursing home: 674 | alone. nursing home: 104 (50%). not nursing home: 199 (30%) | alone. nursing home: 76.4 (6.2); 53-87. not nursing home: 74.7 (7.2); 47-88 | alone. f(149, 72%). not nursing home: 405 (60%) | nursing home: 20.0 (4.1); 10-26. not nursing home: 21.7 (3.6); 10-26 | Dementia | Community, care |
| Wattmo, 2014[281] | Sweden | 1021 | alone: 355 (35%), others: 666 (65%) | alone: 77.1 (6.4); 52-88, others: 74.2 (7.1); 47-88 | alone: f(301, 85%), others: f(353, 53%) | MMSE. alone: 21.4 (3.7); 10-26, others: 21.4 (3.7); 10-26 | Dementia | Community, care |
| Webber, 1994[282] | United States of America | 2505 | alone: 479 (19.1%*), others: 2026 (80.9%*) | alone. <65: 18.4%, 65-74: 22.6%, 75-84: 50.2%, 85+: 8.8%. others. <65: 20.7%, 65-74: 29.3%, 75-84: 41.3%, 85+: 8.7%. | alone: f(84.1%), m(15.9%), others: f(63.6%), m(36.4%) | MMSE. alone: 18.9, others: 16.1 | AD | Community, care |
| Wenborn, 2021[283] | England | 468 | alone: 90 (19.2%), spouse: 352 (75.2%*), others: 26 (5.6%*) | 78.6; 55-97 | f(201), m(267) | MMSE. intervention: 20.7 (5.3), n=249, TAU: 21.4 (4.8), n=219 | AD=247, VaD=92, mixed=60, other=69 | Community |
| Wenger, 1994[284] | England | 61 | alone: 19 (31%), one person: 27 (44%), two+: 14 (23%) | 69-99, 90% 80+ |  |  | Dementia | Community |
| Wilkins, 2007[285] | United States of America | 411 | alone: 201 (48.9%) | 80.9 (7.7); 58-103 | f(321, 78.1%), m(90, 21.9%) | MMSE. 19.07 (7.74); 6-30 | AD=80%, mixed=20% | Community |
| Wong, 2020[286] | Canada | 333 | alone: 69 (20.7%*), spouse: 192 (57.7%*), other: 62 (18.6%*) | <65: median: 59, 44-64, 65+: median: 77, 65-94 | f(194, 58.3%*), m(139, 41.7%*) | MMSE: <65: 25.2 (4.5), 65+: 22.7 (4.3) | MCI: 66 (19.8%), FTD: 37 (11.1%), AD: 170 (51.1%), VaD: 18 (5.45%), DLB: 15 (4.5%), other: 27 (8.1%) | Not stated, likely community |
| Woods, 1991[287] | Scotland | 155 | alone: 58 (37%), spouse: 62 (40%), other: 35 (22%) | female: 79.7, male: 75.9 | f(106), m(49) |  | Dementia | Community |
| Wübbeler, 2015[288] | Germany | 560 | alone: 119 (21.3%) | 79.7 (8.4) | f(325, 58.3%) |  | AD=211 (44.3%), VaD=92 (19.3%), unspecified=155 (32.6%), other=18 (3.8%) | Not stated, likely community and care |
| Yaffe, 2002[289] | United States of America | 5788, split into development: 3859, and validation: 1929 cohorts | alone: 789 (13.6%*) | dev: 78.9 (7.8), validation: 78.8 (7.5) | f(3455, 60%) | MMSE: 14.2 (8.8) | Dementia | Community |

| Zafeiridi, 2020[290] | Northern Ireland | 25418 | alone: 8828 (34.73%), others: 12200 (48%), care home: 4390 (these were excluded from all analysis) | alone: 75.49 (8.50), others: 77.15 (7.82) | f(13338, 63.4%*), m(7690, 36.6%*) |  | Dementia | Community |
| --- | --- | --- | --- | --- | --- | --- | --- | --- |
| Zhao, 2012[291] | France, Switzerland | 122 | alone: 30 (24.8%), others: 91 (75.2%) | 82 (6) | f(78, 63.9%), m(44, 36.1%) | MMSE: 21 (5) | AD | Community, care |

Note: Shading indicates articles using the same study data. * Given percentages or the number of participants included was calculated for this review as these data were not provided in the original paper. ** These are observations rather than participants. The researchers did not provide data for individual participants; they took each timepoint as a separate data point as people with cognitive impairment at one timepoint may not be classified as having cognitive impairment at the others in which they took part.

Abbreviations: Activities of daily living (ADL), China Health and Retirement Longitudinal Study (CHARLS), Clinical Dementia Rating Sum of Boxes (CDR-SoB), Confidence interval (CI), Functional Assessment Staging (FAST), English Longitudinal Study of Ageing (ELSA), Global Deterioration Scale (GDS), Health and Retirement Study (HRS), Hispanic Established Population for the Epidemiological Study of the Elderly (HEPESE), Instrumental activities of daily living (IADL), Long term care (LTC), Mattis Dementia Rating Scale (mDRS), Mexican Health and Aging Study (MHAS), Mini-Mental State Examination (MMSE), Modified Mini-Mental State Examination (3MS), Montreal Cognitive Assessment (MoCA), Survey of Health, Ageing and Retirement in Europe (SHARE),

Dementia type abbreviations: Alzheimer's disease (AD), Dementia with Lewy bodies (DLB), Frontotemporal Dementia (FTD), mild cognitive impairment (MCI), Parkinson's Disease Dementia (PDD), People with dementia (PwD), Vascular dementia (VaD)

Supplementary Table 5. Summary of included qualitative studies

| **Study** | **Country of study** | **n** | **N living alone** | **Mean age** | **Females** | **Severity** | **Dementia type** | **Living situation** |
| --- | --- | --- | --- | --- | --- | --- | --- | --- |
| Andrew, 2022[292] | Scotland | 3 | 3 (100%) | 79 | f (2) |  | Alcohol dementia,  PDD, VaD | Community |
| Baruch, 2004[293] | England | 1 | 1 (100%) |  | f (1) | Mild | Dementia | Community |
| Cott, 2013[294] | Canada | 40 (PwD=20, carers=20) | 20 (100%) | 67-95 | f (15) | MMSE: 20-28 | Cognitive impairment | Community |
| De Witt, 2009[295] | Canada | 8 | 8 (100%) | 78 (58-87) | f (8) | FAST: 4-5 | Dementia | Community |
| De Witt, 2010[296] | Canada | 8 | 8 (100%) | 78 | f (8) | FAST: 4-5 | Dementia | Community |
| De Witt, 2016[297] | Canada | 15 health care professionals:  Hearing counsellors=2, Medicine=2, Nursing=9, Social work=2 |  |  |  |  | Dementia |  |
| Duane, 2013[298] | Australia | 19 | 19 (100%) | female: 79-93  male: 69-91 | f (13) |  | Cognitive impairment or early-stage dementia | Community |
| Evans, 2011[299] | England | 1 | 1 (100%) | 85 | f (1) | Mild to moderate MMSE: 21 | Dementia | Community |
| Evans, 2016[300] | Australia | 21 health professionals:  Social workers, Nurses,  Allied health professionals, Dementia support workers |  |  |  |  | Dementia |  |
| Fowler-Davis,  2020[181] | England | 60 (PwD=30, carers=30 | 30 (100%) | female: 78-96  male: 65-92 | f (23) |  | Dementia | Community |
| Frazer, 2012[301] | England | 8 | 8 (100%) | 83 (82-95) | f (8) | Mild (n=3, MMSE 14-26), moderate (n=5, MMSE 10-19) | AD (n= 5)  Mild dementia (n= 1)  Multi-infarct Dementia (n= 2) | Community |
| Gethin-Jones, 2014[302] | England | Family carers=20 |  |  |  |  | Dementia |  |
| Gilmour, 2003[303] | Northern Ireland | 47 (PwD=10, carers=12, care staff=9, GPs=6, district nurses=4, social workers=6) | 10 (100%) | 83 (74-93) | f (8) | GDS: 4-5 | Dementia | Community |
| Groen-de Ven, 2017[304] | Netherlands | 113 | 10 (8.9%*) | 84 (78-89) | f (7) | Early dementia: 4  Moderate dementia: 5  Advanced dementia: 1 | Dementia | Community (n= 7), care (n=3) |
| Harris, 2006[305] | United States | 15 | 15 (100%) | 75 (62-87) | f (13) | Early-stage AD or MCI | AD (n= 11), AD with Lewy body (n= 1), MCI (n= 3) | Community |
| Heaton, 2021[306] | Wales | 48 (PwD=24, carers=24) | 24 (100%) | 81 (69-91) | f (21) | MMSE: 19-29 | AD (n= 8), VaD (n= 8), Mixed AD and VaD (n= 8) | Community |
| Illiger, 2021[307] | Germany | 12 | 12 (100%) | 65-79 (n= 4)  80-84 (n= 4)  >84 (n = 4) | f (10) |  | Dementia | Community |
| Johannessen, 2018[308] | Norway | 10 | 10 (100%) | 60 (49-67) | f (7) |  | Dementia (Young onset) |  |
| Johannessen, 2019[309] | Norway | 10 | 10 (100%) | 60 (49-67) | f (7) |  | Dementia (Young onset) |  |
| Thorsen, 2020[310] | Norway | 1 | 1 (100%) |  | f (1) |  | Dementia (Young onset) |  |
| Kitamura, 2019[311] | Japan | 10 (PwD=5, carers=5) | 5 (100%) | 83 (78-88) | f (4) | MMSE: 12-26 | AD (n= 3), DLB (n= 2) | Community |
| Knight, 2017[312] | England | 1 | 1 (100%) | 79 | f (1) | Moderate to advanced | AD | Community |
| Kuhn, 2002[313] | United States | 1 | 1 (100%) | 75 | f (1) | Early stage | AD | Community |
| Lloyd, 2015[314] | Australia | 7 | 7 (100%) | 48-85 | f (3) | Early to moderate | Dementia | Community |
| Lussier, 2020[315] | Canada | 1 | 1 (100%) | 91 | f (1) |  | AD | Community |
| Mizuno, 2021[316] | Japan | 1 | 1 (100%) | 70 | f (1) |  | AD | Community |
| Nygård, 2003[317] | Sweden | 10 | 10 (100%) | 82 (75-87) | f (7) | MMSE: 11-27 | AD (n= 6), VaD (n= 2), Mixed (n=1), Unspecified (n=1) | Community |
| Nygård, 2007[318] | Sweden | 8 | 8 (100%) | 69 (57-82) | f (5) | MMSE: 19-28 | AD (n=5), VaD (n=1), other (n=2) | Community |
| Nygård, 2008[239] | Sweden | 8 | 8 (100%) | 69 (57-82 | f (5) | MMSE: 19-28 | AD (n=5), VaD (n=1), other (n=2) | Community |
| Odzakovic, 2020[319] | Sweden | 14 | 4 (28.6%*) | 76 (62-87) | f (3) |  | AD (n= 3), AD and VaD (n=1) | Community |
| Odzakovic, 2021[320] | Sweden, Scotland, England | 14 | 14 (100%) | 79 (62-88) | f (11) |  | AD (n=7), VaD and AD (n=1), VaD (n=1), Unspecified (n=5) | Community |
| Price, 2007[321] | England | 3 | 3 (100%) |  | f (3) | Moderate | VaD (n=1), dementia (n=2) | Community |
| Quail, 2020[322] | China | 1 | 1 (100%) | Late 70’s | f (1) | MMSE: 11, GDS: 5, CDR: 2 | Dementia | Apartment |
| Smith, 2007[323] | United States | 14 | 14 (100%) | Mean by group:  Video: 79.8  Phone: 81.9  Control: 85.5 |  | Mean MMSE score per group:  Video: 23.2, Phone: 22, Control: 25.7 | Dementia | Community |
| Suwa, 2018[324] | Japan | 1 | 1 (100%) | Diary entries start in 2003 (age 92) and continue to 2013 (age 102) | f (1) | HDS-R score. 2003: 23, 2013: 8 | AD | Moved to private nursing home aged 105 after 12 years living at home alone |
| Svanström, 2015[325] | Sweden | 6 | 6 (100%) | 84 (85-90) | f (5) |  | Dementia | Community |
| Waugh, 2009[326] | Australia | Manager: 2  Dementia coordinator: 2  Outreach worker: 1 |  |  |  | Varied from young onset to moderate and severe stages | Dementia |  |
| Zwierenberg, 2018[327] | Netherlands | 63 (PwD=50, case managers=13) |  |  |  | GDS: 2-5 | Dementia |  |

Note: Shading indicates articles using the same study data. * Given percentages or the number of participants included was calculated for this review as these data were not provided in the original paper.

Abbreviations: Clinical Dementia Rating (CDR), Functional Assessment Staging (FAST), General practitioners (GPs), Global Deterioration Scale (GDS), The Revised Hasegawa's Dementia Scale (HDS-R), Mini-Mental State Examination (MMSE), Modified Mini-Mental State Examination (3MS).

Dementia type abbreviations: Alzheimer's disease (AD), Dementia with Lewy bodies (DLB), mild cognitive impairment (MCI), Parkinson's Disease Dementia (PDD), People with dementia (PwD), Vascular dementia (VaD)

References

* indicates articles included in the scoping review

1. Abrams RC, Lachs M, McAvay G, Keohane DJ, Bruce ML: Predictors of self-neglect in community-dwelling elders. *The American Journal of Psychiatry* 2002, 159(10):1724-1730. <https://doi.org/10.1176/appi.ajp.159.10.1724>

2. Barker NN, Himchak MV: Environmental issues affecting elder abuse victims in their reception of community based services. *Journal of Gerontological Social Work* 2006, 48(1/2):233-255. <https://doi.org/10.1300/j083v48n01_16>

3. Bloch F, Lundy JE, Rigaud AS: Profile differences of purchasers, non-purchasers, and users and non-users of Personal Emergency Response Systems: results of a prospective cohort study. *Disability and Health Journal* 2017, 10(4):607-610. <https://doi.org/10.1016/j.dhjo.2017.01.008>

4. Boynton B: Indicators of diogenes syndrome in community dwelling elderly. ProQuest Information & Learning; 2014.

5. McArthur C, Turcotte LA, Sinn C-LJ, Berg K, Morris JN, Hirdes JP: Social engagement and distress among home care recipients during the COVID-19 pandemic in Ontario, Canada: a retrospective cohort study. *Journal of the American Medical Directors Association* 2022, 23(7):1101-1108. <https://doi.org/10.1016/j.jamda.2022.04.005>

6. Andrews J: Maintaining continence in people with dementia. *Nursing Times* 2013, 109(27):20-21.

7. Barry HE, Hughes CM: Managing medicines in the time of COVID-19: implications for community-dwelling people with dementia. *International Journal of Clinical Pharmacy* 2021, 43(1):275-279. <https://doi.org/10.1007/s11096-020-01116-y>

8. Gilmour H: Living alone with dementia: risk and the professional role. *Nursing Older People* 2004, 16(9):20-24. <https://doi.org/10.7748/nop2004.12.16.9.20.c2349>

9. Griffith R, Channon C: Wills and the district nurse: the importance of caution. *British Journal of Community Nursing* 2005, 10(8):387-391. <https://doi.org/10.12968/bjcn.2005.10.8.18580>

10. Keady J: Living alone with dementia. *British Journal of Nursing* 1994, 3(13):648-650. <https://doi.org/10.12968/bjon.1994.3.13.648>

11. Kikuchi K, Ooguchi T, Ikeuchi T, Ito K, Awata S: Current status and issues of missing older persons with dementia living alone in Japan. *Geriatrics & Gerontology International* 2022, 22(8):684-686. <https://doi.org/10.1111/ggi.14434>

12. Kikuchi K, Ikeuchi T, Awata S: A study on the incidence rate of missing persons with dementia living alone in Chiba prefecture, Japan. *Geriatrics & Gerontology International* 2023, 23(11):890-891. <https://doi.org/10.1111/ggi.14695>

13. Kolanowski A, Fortinsky RH, Calkins M, Devanand DP, Gould E, Heller T, Hodgson NA, Kales HC, Kaye J, Lyketsos C *et al*: Advancing research on care needs and supportive approaches for persons with dementia: recommendations and rationale. *Journal of the American Medical Directors Association* 2018, 19(12):1047-1053. <https://doi.org/10.1016/j.jamda.2018.07.005>

14. Medical Ethics Advisor: Older adults living alone with cognitive impairment lack support. *Medical Ethics Advisor* 2018, 34(7).

15. Miyamae F, Taga T, Okamura T, Awata S: Toward a society where people with dementia 'living alone' or 'being a minority group' can live well. *Psychogeriatrics* 2022, 22(4):586-587. <https://doi.org/10.1111/psyg.12836>

16. The National LGB&T Partnership: The dementia challenge for LGBT communities: a paper based on a roundtable discussion 2 December 2014: National LGB&T Partnership; 2015.

17. Newhouse BJ, Niebuhr L, Stroud T, Newhouse E: Living alone with dementia: innovative support programs. *Alzheimer's Care Quarterly* 2001, 2(2):53-61.

18. Portacolone E: On living alone with Alzheimer's disease. *Care Weekly* 2018, 2018:1-4. <https://doi.org/10.14283/cw.2018.3>

19. Portacolone E, Halpern J, Luxenberg J, Harrison KL, Covinsky KE: Ethical issues raised by the introduction of artificial companions to older adults with cognitive impairment: a call for empowerment. *Alzheimer's & Dementia* 2019, 15(Supplement 7):P550. <https://doi.org/10.1016/j.jalz.2019.06.4484>

20. Portacolone E, Torres JM, Johnson JK, Benton D, Rapp T, Tran T, Martinez P, Graham C: The living alone with cognitive impairment project's policy advisory group on long-term services and supports: setting a research equity agenda. *International Journal of Environmental Research and Public Health* 2022, 19(10). <https://doi.org/10.3390/ijerph19106021>

21. Regal P, Heatherington E: Baseline instrumental activities of daily living and incident dementia. *Journal of the American Geriatrics Society* 2012, 60(6):1189-1190. <https://doi.org/10.1111/j.1532-5415.2012.03986.x>

22. Robinson H: 'I have Alzheimer's disease and I have a voice'. *Nursing Older People* 2017, 29(5):14. <https://doi.org/10.7748/nop.29.5.14.s18>

23. Soniat BA: Dementia patients who live alone: research and clinical challenges. *Journal of the American Geriatrics Society* 2004, 52(9):1576-1577. <https://doi.org/10.1111/j.1532-5415.2004.52427.x>

24. Starns MK, Karner TX, Montgomery RJ: Exemplars of successful Alzheimer's demonstration projects. *Home Health Care Services Quarterly* 2002, 21(3-4):141-175. <https://doi.org/10.1300/J027v21n03_08>

25. Tierney MC: How safe are cognitively impaired seniors who live alone? *Canadian Journal on Aging* 1997, 16(2):177-182. <https://doi.org/10.1017/S0714980800014288>

26. Webb C: In response to: de Witt L., Ploeg J. & Black M. (2010) Living alone with dementia: an interpretive phenomenological study with older women. Journal of Advanced Nursing 66(8), 1697-1706. *Journal of Advanced Nursing* 2010, 66(9):2138. <https://doi.org/10.1111/j.1365-2648.2010.05389.x>

27. Casaccia S, Revel GM, Scalise L, Bevilacqua R, Rossi L, Serrano A, Karkowski I, Marconi F, Suijkerbuijk S, Lukkien D *et al*: A context-aware social robot to improve the quality of life of people with dementia. International Society for Gerontechnology's (ISG) 12th World Conference of Gerontechnology, October 6-9, 2020 (Virtual). *Gerontechnology* 2020, 19:55. <https://doi.org/10.4017/gt.2020.19.s.69926>

28. Flatt JD, Pollock K, Lee R, Song Y, Wharton W, Anderson JG: Feasibility of the Savvy Caregiver program for care providers of LGBTQ adults living with Alzheimer's disease and related dementias. *Alzheimer's & Dementia* 2021, 17(Supplement 8):e055633. <https://doi.org/10.1002/alz.055633>

29. Wiese LK, Williams CL, Galvin JE, Hain DD: Cognitive screening in rural Florida subsidized housing units among ethnically diverse older adults. *Alzheimer's & Dementia* 2018, 14(Supplement 7):P559. <https://doi.org/10.1016/j.jalz.2018.06.596>

30. Bourennane W, Charlon Y, Bettahar F, Campo E, Esteve D: Homecare monitoring system: a technical proposal for the safety of the elderly experimented in an Alzheimer's care unit. *IRBM* 2013, 34(2):92-100. <https://doi.org/10.1016/j.irbm.2013.02.002>

31. Campbell S, Manthorpe J, Samsi K, Abley C, Robinson L, Watts S, Bond J, Keady J: Living with uncertainty: mapping the transition from pre-diagnosis to a diagnosis of dementia. *Journal of Aging Studies* 2016, 37:40-47. <https://doi.org/10.1016/j.jaging.2016.03.001>

32. Cañabate P, Martin E, Moreno M, Preckler S, Ortega G, Hernandez I, Rosende-Roca M, Vargas L, Mauleon A, Rodriguez O *et al*: An interdisciplinary portrait of dementia: The reality of Barcelona, Spain. *Alzheimer's & Dementia* 2015, 11(Supplement 7):P600. <https://doi.org/10.1016/j.jalz.2015.06.824>

33. Chou H-C: Remote monitoring and control smart floor for detecting falls and wandering patterns in people with dementia. *Journal of Intelligent & Fuzzy Systems* 2019, 36(2):1323-1331. <https://doi.org/10.3233/jifs-169903>

34. Chu IP: Family care of the elderly with dementia in Hong Kong. *International Social Work* 1991, 34(4):365-372. <https://doi.org/10.1177/002087289103400405>

35. Cloutier DS, Penning MJ: Janus at the crossroads: perspectives on long-term care trajectories for older women with dementia in a Canadian context. *The Gerontologist* 2017, 57(1):68-81. <https://doi.org/10.1093/geront/gnw158>

36. Cunningham NA, Cowie J, Methven K: Right at home: living with dementia and multi-morbidities. *Ageing & Society* 2022, 42(3):632-656. <https://doi.org/10.1017/s0144686x2000104x>

37. de Medeiros K, Berlinger N, Girling L: Not wanting to lose the dignity of risk: on living alone with dementia. *Perspectives in Biology and Medicine* 2022, 65(2):274-282. <https://doi.org/10.1353/pbm.2022.0023>

38. Dean J, Silversides K, Crampton J, Wrigley J: Evaluation of the Bradford Dementia Friendly Communities programme. 2015.

39. Dickins M, Goeman D, O'Keefe F, Iliffe S, Pond D: Understanding the conceptualisation of risk in the context of community dementia care. *Social Science & Medicine* 2018, 208(1):72-79. <https://doi.org/10.1016/j.socscimed.2018.05.018>

40. Domínguez-Berjón MF, Esteban-Vasallo MD, Zoni AC, Gènova-Maleras R, Astray-Mochales J: Place of death and associated factors among patients with amyotrophic lateral sclerosis in Madrid (Spain). *Amyotrophic Lateral Sclerosis and Frontotemporal Degeneration* 2015, 17(1-2):62-68. <https://doi.org/10.3109/21678421.2015.1089908>

41. Doron I, Werner P, Spanier B, Lazar O: The legal appearances of dementia in court rulings: mapping the terrain. *International Psychogeriatrics* 2017, 29(5):755-763. <https://doi.org/10.1017/s1041610216002453>

42. Doughty K, Williams G, King PJ, Woods R: DIANA - A telecare system for supporting dementia sufferers in the community. In: *20th Annual International Conference of the IEEE-Engineering-in-Medicine-and-Biology-Society: Oct 29-Nov 01 1998; Hong Kong, Peoples R China*. 1998: 1980-1983. <https://doi.org/10.1109/IEMBS.1998.746991>

43. Evans SC, Atkinson T, Cameron A, Johnson EK, Smith R, Darton R, Porteus J, Lloyd L: Can extra care housing support the changing needs of older people living with dementia? *Dementia* 2020, 19(5):1492-1508. <https://doi.org/10.1177/1471301218801743>

44. Fæo SE, Tranvåg O, Samdal R, Husebo BS, Bruvik FK: The compound role of a coordinator for home-dwelling persons with dementia and their informal caregivers: qualitative study. *BMC Health Services Research* 2020, 20(1):1045. <https://doi.org/10.1186/s12913-020-05913-z>

45. Fredriksen-Goldsen K, Teri L, Kim HJ, Goldsen J, McKenzie G, La Fazia DM: Aging with pride, idea: lessons learned from the first tested clinical intervention addressing dementia and Alzheimer's disease in the LGBT community. *Alzheimer's & Dementia* 2019, 15(Supplement 7):P206-P207. <https://doi.org/10.1016/j.jalz.2019.06.4546>

46. Ganzini L, Johnston WS, Hoffman WF: Correlates of suffering in amyotrophic lateral sclerosis. *Neurology* 1999, 52(7):1434-1440. <https://doi.org/10.1212/wnl.52.7.1434>

47. Gold DP, Reis MF, Markiewicz D, Andres D: When home caregiving ends - a longitudinal-study of outcomes for caregivers of relatives with dementia. *Journal of the American Geriatrics Society* 1995, 43(1):10-16. <https://doi.org/10.1111/j.1532-5415.1995.tb06235.x>

48. Goldfarb D, Allen AM, Nisson LE, Petitti DB, Saner D, Langford C, Burke WJ, Reiman EM, Atri A, Tariot PN: Design and development of a community-based, interdisciplinary, collaborative dementia care program. *The American Journal of Geriatric Psychiatry* 2022, 30(6):651-660. <https://doi.org/10.1016/j.jagp.2021.10.014>

49. González AB, Selmes M, Selmes J: Can smart homes extend people with Alzheimer's disease stay at home? *Journal of Enabling Technologies* 2017, 11(1):6-12. <https://doi.org/10.1108/jet-12-2015-0039>

50. Gordon C: Guardianship in Oxfordshire: Hits and misses. *Psychiatric Bulletin* 1998, 22(4):233-235. <https://doi.org/10.1192/pb.22.4.233>

51. Gwyther LP: The perspective of the person with Alzheimer disease: which outcomes matter in early to middle stages of dementia? *Alzheimer Disease & Associated Disorders* 1997, 11(Supplement 6):18-24.

52. Harrison KL, Garrett SB, Halim M, Sideman AB, Allison TA, Dohan D, Naasan G, Miller BL, Smith AK, Ritchie CS: "I didn't sign up for this": perspectives from persons living with dementia and care partners on challenges, supports, and opportunities to add geriatric neuropalliative care to dementia specialty care. *Journal of Alzheimers Disease* 2022, 90(3):1301-1320. <https://doi.org/10.3233/jad-220536>

53. Hildick Smith M: Parkinson's disease in the elderly. *Journal of the Royal Society of Health* 1983, 103(5):166-169. <https://doi.org/10.1177/146642408310300503>

54. Hobson E, Baird W, Bradburn M, Cooper C, Mawson S, Quinn A, Shaw PJ, Walsh T, McDermott CJ: Process evaluation and exploration of telehealth in motor neuron disease in a UK specialist centre. *BMJ Open* 2019, 9(10):e028526. <https://doi.org/10.1136/bmjopen-2018-028526>

55. Hossain MA, Ray SK, Harris G, Ahmed S, Ieee: An emergency response system to support early stage dementia patients. In: *IEEE Symposium on Computers and Communications (ISCC): Jun 30-Jul 03 2022; Rhodes, Greece*. 2022. <https://doi.org/10.1109/iscc55528.2022.9912916>

56. Hughes AM: Caregivers of family members with dementia were involved in a dynamic decision making process to maintain tolerable situations. *Evidence Based Nursing* 2000, 3(1):32-32. <https://doi.org/10.1136/ebn.3.1.32>

57. James CM, Harper PS, Wiles CM: Motor neurone disease-a study of prevalence and disability. *Quarterly Journal of Medicine* 1994, 87(11):693-699.

58. King APY, Siu KWM: Participant observation in cognitive gameplay as a rehabilitation tool for living alone elderly with dementia in hong kong: a pilot study. *The Design Journal* 2017, 20(Supplement 1):S2426-S2438. <https://doi.org/10.1080/14606925.2017.1352756>

59. Lancioni GE, Singh NN, O'Reilly MF, Sigafoos J, Ferlisi G, Ferrarese G, Zullo V, Addante LM, Spica A, Oliva D: Technology-aided programs for assisting communication and leisure engagement of persons with amyotrophic lateral sclerosis: Two single-case studies. *Research in Developmental Disabilities* 2012, 33(5):1605-1614. <https://doi.org/10.1016/j.ridd.2012.03.028>

60. Lebert F: Diogene syndrome, a clinical presentation of fronto-temporal dementia or not? *International Journal of Geriatric Psychiatry* 2005, 20(12):1203-1204. <https://doi.org/10.1002/gps.1430>

61. Macleod AD, Counsell CE: Predicting poor functional outcome in Parkinson's disease. *Movement Disorders* 2016, 31(Supplement 2):S155. <https://doi.org/10.1002/mds.26688>

62. Maunsell RH, Bloomfield S, Erridge C, Foster C, Hardcastle M, Hogden A, Kidd A, Lisiecka D, McDermott CJ, Morrison K *et al*: Developing a web-based patient decision aid for gastrostomy in motor neurone disease. *Clinical Nutrition ESPEN* 2020, 35(1):P235. <https://doi.org/10.1016/j.clnesp.2019.12.062>

63. McGoldrick C, Crawford S, Evans JJ: MindMate: A single case experimental design study of a reminder system for people with dementia. *Neuropsychological Rehabilitation* 2021, 31(1):18-38. <https://doi.org/10.1080/09602011.2019.1653936>

64. Meuleners LB, Hobday MB: A population-based study examining injury in older adults with and without dementia. *Journal of the American Geriatrics Society* 2017, 65(3):520-525. <https://doi.org/10.1111/jgs.14523>

65. Naharci MI, Kayahan Satis N, Ozsurekci C, Tasci I: Assessment of clinical features and coexisting geriatric syndromes in newly diagnosed dementia with Lewy bodies: a retrospective study in a tertiary geriatrics setting in Turkey. *European Geriatric Medicine* 2023, 14(1):19-27. <https://doi.org/10.1007/s41999-022-00727-0>

66. Nwabuobi L, Barbosa W, Sweeney M, Oyler S, Meisel T, Di Rocco A, Chodosh J, Fleisher JE: Sex-related differences in homebound advanced Parkinson's disease patients. *Clinical Interventions in Aging* 2019, 14(1):1371-1377. <https://doi.org/10.2147/cia.S203690>

67. Ohta Y, Yamashita T, Hishikawa N, Sato K, Hatanaka N, Takemoto M, Doutare S, Abe K: Affective improvement of neurological disease patients and caregivers using an automated telephone call service. *Journal of Clinical Neuroscience* 2018, 56(1):74-78. <https://doi.org/10.1016/j.jocn.2018.07.006>

68. Pentecost C, Collins R, Stapley S, Victor C, Quinn C, Hillman A, Litherland R, Allan L, Clare L: Effects of social restrictions on people with dementia and carers during the pre-vaccine phase of the COVID-19 pandemic: Experiences of IDEAL cohort participants. *Health and Social Care in the Community* 2022, 30(6):e4594-e4604. <https://doi.org/10.1111/hsc.13863>

69. Pfalzer AC, Hale LM, Huitz E, Buchanan DA, Brown BK, Moroz S, Rouleau RM, Hay KR, Hoadley J, Laird A *et al*: Healthcare delivery and huntington's disease during the time of COVID-19. *Journal of Huntingtons Disease* 2021, 10(2):313-322. <https://doi.org/10.3233/jhd-200460>

70. Pizzi LT, Jutkowitz E, Prioli KM, Lu E, Babcock Z, McAbee-Sevick H, Wakefield DB, Robison J, Molony S, Piersol CV *et al*: Cost-benefit analysis of the COPE program for persons living with dementia: toward a payment model. *Innovation in Aging* 2022, 6(1):igab042. <https://doi.org/10.1093/geroni/igab042>

71. Rahja M, Comans T, Clemson L, Crotty M, Laver K: Are there missed opportunities for occupational therapy for people with dementia? An audit of practice in Australia. *Australian Occupational Therapy Journal* 2018, 65(6):565-574. <https://doi.org/10.1111/1440-1630.12514>

72. Read S, Waterman H, Morgan JE, Harper RA, Spencer AF, Stanford P: Glaucoma, dementia, and the "precipice of care": transitions between states of medication adherence. *Patient Preference and Adherence* 2018, 12(1):1315-1325. <https://doi.org/10.2147/ppa.S167080>

73. Read S, Hu B, Wittenberg R, Brimblecombe N, Robinson L, Banerjee S: A longitudinal study of functional unmet need among people with dementia. *Journal of Alzheimer's Disease* 2021, 84(2):705-716. <https://doi.org/10.3233/jad-210724>

74. Rees J, Burton A, Walters K, Cooper C: Exploring the provision and support of care for long-term conditions in dementia: A qualitative study combining interviews and document analysis. *Dementia* 2023, 22(4):820-837. <https://doi.org/10.1177/14713012231161854>

75. Risi L, Brown J, Hodkinson I, Sugarhood P, Thormod C: Integrated person centred support preparation - a handy approach. *International Journal of Integrated Care* 2016, 16(6):1-2. <https://doi.org/10.5334/ijic.2888>

76. Savitch N, Abbott E, Parker G: Dementia: through the eyes of women. York: Joseph Rowntree Foundation (JRF); 2015.

77. Tsunawaki S, Abe M, DeJonckheere M, Cigolle CT, Philips KK, Rubinstein EB, Matsuda M, Fetters MD, Inoue M: Primary care physicians' perspectives and challenges on managing multimorbidity for patients with dementia: a Japan-Michigan qualitative comparative study. *BMC Primary Care* 2023, 24(1):132. <https://doi.org/10.1186/s12875-023-02088-4>

78. Walker R, Sweeney W, Gray W: Access to care services for rural dwellers with idiopathic Parkinson's disease. *British Journal of Neuroscience Nursing* 2011, 7(2):494-496. <https://doi.org/10.12968/bjnn.2011.7.2.494>

79. Watts DT, Cassel CK, Howell T: Dangerous behavior in a demented patient. Preserving autonomy in a patient with diminished competence. *Journal of the American Geriatrics Society* 1989, 37(7):658-662. <https://doi.org/10.1111/j.1532-5415.1989.tb01258.x>

80. Zafeiridi E, McMichael A, O'Hara L, Passmore P, McGuinness B: Hospital admissions and emergency department visits for people with dementia. *QJM* 2023, 117(2):119-124. <https://doi.org/10.1093/qjmed/hcad232>

81. Barry HE, McGrattan M, Ryan C, Passmore AP, Robinson AL, Molloy GJ, Darcy CM, Buchanan H, Hughes CM: Perspectives of community-dwelling people with dementia and their carers about medicines management: a qualitative study. *International Journal of Pharmacy Practice* 2019, 27(Supplement 2):15-16. <https://doi.org/10.1111/ijpp.12532>

82. Cambridge P, Beadle-Brown J, Milne A, Mansell J, Whelton B: Exploring the incidence, risk factors, nature and monitoring of adult protection alerts. Canterbury: University of Kent. Tizard Centre; 2006.

83. Chen YH, Lebouvier T, Skrobala E, Volpe-Gillot L, Huvent-Grelle D, Jourdan N, Leroy M, Richard F, Pasquier F, Meotis N: Twenty-year trends in patient referrals throughout the creation and development of a regional memory clinic network. *Alzheimer's & Dementia: Translational Research & Clinical Interventions* 2020, 6(1):e12048. <https://doi.org/10.1002/trc2.12048>

84. Chen S, Zhang H, Underwood BR, Wang D, Chen X, Cardinal RN: Trends in gender and racial/ethnic disparities in physical disability and social support among U.S. older adults with cognitive impairment living alone, 2000-2018. *Innovation in Aging* 2023, 7(4):igad028. <https://doi.org/10.1093/geroni/igad028>

85. Dura-Perez E, Goodman-Casanova JM, Vega-Nuñez A, Guerrero-Pertiñez G, Varela-Moreno E, Garolera M, Quintana M, Cuesta-Vargas AI, Barnestein-Fonseca P, Gómez Sánchez-Lafuente C *et al*: The impact of COVID-19 confinement on cognition and mental health and technology use among socially vulnerable older people: retrospective cohort study. *Journal of Medical Internet Research* 2022, 24(2):e30598. <https://doi.org/10.2196/30598>

86. Edwards RD, Brenowitz WD, Portacolone E, Covinsky KE, Bindman A, Glymour MM, Torres JM: Difficulty and help with activities of daily living among older adults living alone with cognitive impairment. *Alzheimer's & Dementia* 2020, 16(8):1125-1133. <https://doi.org/10.1002/alz.12102>

87. Fæo SE, Husebo BS, Bruvik FK, Tranvåg O: "We live as good a life as we can, in the situation we're in" - the significance of the home as perceived by persons with dementia. *BMC Geriatrics* 2019, 19(1):158. <https://doi.org/10.1186/s12877-019-1171-6>

88. Feng M, Igarashi A, Yamamoto-Mitani N, Watanabe-Noguchi M, Yoshie S, Iijima K: Care management agency affects the service cost of home help and day care: a population-based study in Japan. *Journal of the American Geriatrics Society* 2016, 64(Supplement 1):S251. <https://doi.org/10.1111/jgs.14231>

89. Field B, Coates E, Mountain G: What influences uptake of psychosocial interventions by people living with early dementia? A qualitative study. *Dementia* 2021, 20(8):2668-2688. <https://doi.org/10.1177/14713012211007397>

90. Gill TM, Richardson ED, Tinetti ME: Evaluating the risk of dependence in activities of daily living among community-living older adults with mild to moderate cognitive impairment. *The Journals of Gerontology Series A: Biological Sciences and Medical Sciences* 1995, 50(5):M235-241. <https://doi.org/10.1093/gerona/50a.5.m235>

91. Goodman-Casanova JM, Dura-Perez E, Guzman-Parra J, Cuesta-Vargas A, Mayoral-Cleries F: Telehealth home support during COVID-19 confinement for community-dwelling older adults with mild cognitive impairment or mild dementia: survey study. *Journal of Medical Internet Research* 2020, 22(5):e19434. <https://doi.org/10.2196/19434>

92. Green-Harris G, Houston S, Skora T, Farrar-Edwards D: Connecting the dots: Meeting the diagnostic and treatment needs of underserved urban African American elders with memory loss. *Alzheimer's & Dementia* 2016, 12(7 Supplement):P793. <https://doi.org/10.1016/j.jalz.2016.06.1597>

93. Guzman-Parra J, Barnestein-Fonseca P, Guerrero-Pertiñez G, Anderberg P, Jimenez-Fernandez L, Valero-Moreno E, Goodman-Casanova JM, Cuesta-Vargas A, Garolera M, Quintana M *et al*: Attitudes and use of information and communication technologies in older adults with mild cognitive impairment or early stages of dementia and their caregivers: cross-sectional study. *Journal of Medical Internet Research* 2020, 22(6):e17253. <https://doi.org/10.2196/17253>

94. Hagiwara C, Sasaki H: Conditions and characteristics of older adults and primary caregivers who use short-stay services: a comparison between long-term and short-term service groups by service type. *Journal of Rural Medicine* 2022, 17(4):196-204. <https://doi.org/10.2185/jrm.2022-013>

95. Hashimoto M, Suzuki M, Hotta M, Nagase A, Yamamoto Y, Hirakawa N, Nagata Y, Satake Y, Suehiro T, Kanemoto H *et al*: The influence of the COVID-19 outbreak on the lifestyle of older patients with dementia or mild cognitive impairment who live alone. *Frontiers in Psychiatry* 2020, 11(1):570580. <https://doi.org/10.3389/fpsyt.2020.570580>

96. Ishii H, Kimino K, Aljehani M, Ohe N, Inoue M: An early detection system for dementia using the M2M/IoT platform. *Procedia Computer Science* 2016, 96:1332-1340. <https://doi.org/10.1016/j.procs.2016.08.178>

97. Ito K, Okamura T, Tsuda S, Awata S: Diogenes syndrome in a 10-year retrospective observational study: An elderly case series in Tokyo. *International Journal of Geriatric Psychiatry* 2022, 37(1). <https://doi.org/10.1002/gps.5635>

98. Jarrett P, McCloskey R, Stewart C, McCollum AR, Oakley H: Long stay hospital patients in acute care hospital beds-what does dementia have to do with this? *The American Journal of Geriatric Psychiatry* 2013, 21(3 Supplement 1):S141. <https://doi.org/10.1016/j.jagp.2012.12.187>

99. Jurkeviciute M, van Velsen L, Eriksson H, Lifvergren S, Trimarchi PD, Andin U, Svensson J: Identifying the value of an ehealth intervention aimed at cognitive impairments: observational study in different contexts and service models. *Journal of Medical Internet Research* 2020, 22(10):e17720. <https://doi.org/10.2196/17720>

100. Kadoya Y, Khan MSR, Oba H, Narumoto J: Factors affecting knowledge about the adult guardianship and civil trust systems: evidence from Japan. *Journal of Women & Aging* 2021, 33(5):541-555. <https://doi.org/10.1080/08952841.2020.1727711>

101. Steketee G, Frost RO, Kim H: Hoarding by elderly people. *Health & Social Work* 2001, 26(3):176-184. <https://doi.org/10.1093/hsw/26.3.176>

102. Kirk A, Philippon O, Karunanayake C, Morgan D: Differences between men and women referred to a rural and remote memory clinic. *Journal of the Neurological Sciences* 2017, 381(Supplement 1):671. <https://doi.org/10.1016/j.jns.2017.08.1889>

103. Kullberg K, Aberg AC, Bjorklund A, Ekblad J, Sidenvall B: Daily eating events among co-living and single-living, diseased older men. *The Journal of Nutrition, Health and Aging* 2008, 12(3):176-182. <https://doi.org/10.1007/bf02982615>

104. Larsson K, Thorslund M, Forsell Y: Dementia and depressive symptoms as predictors of home help utilization among the oldest old: population-based study in an urban area of Sweden. *Journal of Aging and Health* 2004, 16(5):641-668. <https://doi.org/10.1177/0898264304268586>

105. Livingston G, Manela M, Katona C: Cost of community care for older people. *British Journal of Psychiatry* 1997, 171(1):56-59. <https://doi.org/10.1192/bjp.171.1.56>

106. Lu LT, Huang CM, Huang SF, Wu SI, Guo JL: Perspectives of the elderly with mild cognitive impairment living alone on participating in a dementia prevention program: a Q methodology study. *International Journal of Environmental Research and Public Health* 2020, 17(21):7712. <https://doi.org/10.3390/ijerph17217712>

107. Luppa M, Luck T, Matschinger H, Konig HH, Riedel-Heller SG: Predictors of institutionalization in individuals with and without dementia - Results from the leipzig longitudinal study of the aged (LEILA75+). *Psychiatrische Praxis* 2011, 38(Supplement 1):P38_EC. <https://doi.org/10.1055/s-0031-1277903>

108. Machesney D, Wexler SS, Chen T, Coppola JF: Gerontechnology companion: virutal pets for dementia patients. In: *IEEE Long Island Systems, Applications and Technology Conference (LISAT): May 02 2014; Farmingdale, NY*. 2014. <https://doi.org/10.1109/LISAT.2014.6845226>

109. Muurling M, Au-Yeung WM, Beattie Z, Wu CY, Dodge H, Rodrigues NK, Gothard S, Silbert LC, Barnes LL, Steele JS *et al*: Differences in life space activity patterns between older adults with mild cognitive impairment living alone or as a couple: cohort study using passive activity sensing. *JMIR Aging* 2023, 6(1):e45876. <https://doi.org/10.2196/45876>

110. Newman SJ: The accuracy of reports on housing and neighborhood conditions by persons with severe mental illness. *Psychosocial Rehabilitation Journal* 1995, 18(3):129-136. <https://doi.org/10.1037/h0095495>

111. Nordloh GE: The influence of marriage and other factors on early diagnosis seeking for Alzheimer's disease. ProQuest Information & Learning; 2011.

112. Oxford Brookes University Institute of Public Care: Oxfordshire County Council: support to the early intervention and prevention services for older people and vulnerable adults programme: report on study of care pathways. Oxford: Oxford Brookes University. Institute of Public Care; 2010.

113. Portacolone E, Johnson JK, Covinsky KE, Halpern J, Rubinstein RL: The effects and meanings of receiving a diagnosis of mild cognitive impairment or Alzheimer's disease when one lives alone. *Journal of Alzheimer's Disease* 2018, 61(4):1517-1529. <https://doi.org/10.3233/jad-170723>

114. Portacolone E, Rubinstein RL, Covinsky KE, Halpern J, Johnson JK: The precarity of older adults living alone with cognitive impairment. *The Gerontologist* 2019, 59(2):271-280. <https://doi.org/10.1093/geront/gnx193>

115. Portacolone E, Chodos A, Halpern J, Covinsky KE, Keiser S, Fung J, Rivera E, Tran T, Bykhovsky C, Johnson JK: The effects of the Covid-19 pandemic on the lived experience of diverse older adults living alone with cognitive impairment. *The Gerontologist* 2021, 61(2):251-261. <https://doi.org/10.1093/geront/gnaa201>

116. Robertson O, Kirk A, Karunanayake C, Morgan D, O'Connell ME: Women and men presenting to a rural and remote memory clinic in Saskatchewan, Canada show similar cognitive findings for dementia. *Alzheimer Disease & Associated Disorders* 2020, 34(2):183-187. <https://doi.org/10.1097/wad.0000000000000307>

117. Rockwood K, Lin L, Mitnitski A, Zeng A, Leibman C, Mucha L: Dementia symptom profiles in relation to stage. *Alzheimer's & Dementia* 2011, 7(Supplement 4):S544. <https://doi.org/10.1016/j.jalz.2011.05.1536>

118. Schiedermayer DL, Duthie EH, Shelley MV: Emergency detention of the elderly: Demographics, diagnoses, and outcome. *Journal of the American Geriatrics Society* 1982, 30(6):383-386. <https://doi.org/10.1111/j.1532-5415.1982.tb02836.x>

119. Schwartz LB: The importance of health-related quality of life in persons with cognitive impairment. ProQuest Information & Learning; 2013.

120. Senesi B, Prete C, Zigoura E, Pinna A, Siri G, Zora S, Novello C, Solfrizzi V, Custodero C, Cella A *et al*: Feasibility, acceptability and usefulness of a telemedicine (TM) program in older people with dementia during COVID-19 pandemic. *European Geriatric Medicine* 2020, 11(Supplement 1):S102. <https://doi.org/10.1007/s41999-020-00428-6>

121. Sibley A, MacKnight C, Rockwood K, Fisk J, Gauthier S, Guzman DA, Hogan DB, Consortium to Investigate Vascular Impairment of Cognition: The effect of the living situation on the severity of dementia at diagnosis. *Dementia and Geriatric Cognitive Disorders* 2002, 13(1):40-45. <https://doi.org/10.1159/000048632>

122. Tan TH, Gochoo M, Jean FR, Huang SC, Kuo SY: Front-door event classification algorithm for elderly people living alone in smart house using wireless binary sensors. *IEEE Access* 2017, 5(1):10734-10743. <https://doi.org/10.1109/access.2017.2711495>

123. Taylor JS, Figueroa Gray MS, Mar CM, Crane PK, Kariya H, Freitag C, Taneja P, Ramaprasan A, Shell-Duncan B, O'Hare AM *et al*: Kinless older adults with dementia: qualitative analysis of data from the adult changes in thought study. *The Journals of Gerontology, Series B: Psychological Sciences and Social Sciences* 2023, 78(6):1060-1072. <https://doi.org/10.1093/geronb/gbad030>

124. Tsuda S, Inagaki H, Okamura T, Sugiyama M, Ogawa M, Miyamae F, Edahiro A, Ura C, Sakuma N, Awata S: Promoting cultural change towards dementia friendly communities: a multi-level intervention in Japan. *BMC Geriatrics* 2022, 22(1):360. <https://doi.org/10.1186/s12877-022-03030-6>

125. Walker M, Orrell M, Manela M, Livingston G, Katona C: Do health and use of services differ in residents of sheltered accommodation? A pilot study. *International Journal of Geriatric Psychiatry* 1998, 13(9):617-624. <https://doi.org/10.1002/(sici)1099-1166(199809)13:9><617::aid-gps833>3.0.co;2-3

126. Wrigley M, Cooney C: Diogenes syndrome: An Irish series. *Irish Journal of Psychological Medicine* 1992, 9(1):37-41. <https://doi.org/10.1017/S0790966700013896>

127. Xu E, Kim P, Yee C, Zhang M, Reckrey J, Lubetsky S, Ornstein K, Franzosa E: "I depend on her for everything": A mixed-methods analysis of the role of home care workers caring for medically and socially complex patients during the COVID-19 pandemic. *Journal of the American Geriatrics Society* 2022, 70(Supplement 1):S301. <https://doi.org/10.1111/jgs.v70.S1>

128. Yang YL, Swinnerton K, Portacolone E, Allen IE, Torres JM, Duchowny K: Difficulties with activities of daily living and receipt of care among older adults with cognitive impairment: differences between those living alone and those living with others. *Journal of Alzheimers Disease* 2022, 89(1):31-37. <https://doi.org/10.3233/jad-220172>

129.* Abel B, Eckert T, Pomiersky R, Dautel A, Schäufele M, Pfeiffer K, Hauer K: Transition from inpatient rehabilitation to the home environment in cognitively impaired older persons after hip fracture. *Journal of Rehabilitation Medicine* 2020, 52(11):jrm00130. <https://doi.org/10.2340/16501977-2757>

130.* Amjad H, Roth DL, Samus QM, Yasar S, Wolff JL: Potentially unsafe activities and living conditions of older adults with dementia. *Journal of the American Geriatrics Society* 2016, 64(6):1223-1232. <https://doi.org/10.1111/jgs.14164>

131.* Amjad H, Roth DL, Sheehan OC, Lyketsos CG, Wolff JL, Samus QM: Underdiagnosis of dementia: an observational study of patterns in diagnosis and awareness in US older adults. *Journal of General Internal Medicine* 2018, 33(7):1131-1138. <https://doi.org/10.1007/s11606-018-4377-y>

132.* Okoye SM, Fabius CD, Reider L, Wolff JL: Predictors of falls in older adults with and without dementia. *Alzheimer's & Dementia* 2023, 19(7):2888-2897. <https://doi.org/10.1002/alz.12916>

133.* Andrieu S, Coley N, Rolland Y, Cantet C, Arnaud C, Guyonnet S, Nourhashemi F, Grand A, Vellas B, group P: Assessing Alzheimer's disease patients' quality of life: discrepancies between patient and caregiver perspectives. *Alzheimer's & Dementia* 2016, 12(4):427-437. <https://doi.org/10.1016/j.jalz.2015.09.003>

134.* Nourhashemi F, Gillette-Guyonnet S, Andrieu S, Rolland Y, Ousset PJ, Vellas B, Berrut, Barre, Rainfra Y, Harston *et al*: A randomized trial of the impact of a specific care plan in 1120 Alzheimer's patients (PLASA study) over a two-year period: design and baseleve data. *Journal of Nutrition, Health and Aging* 2008, 12(4):263-271. <https://doi.org/10.1007/BF02982632>

135.* Soto M, Andrieu S, Gares V, Cesari M, Gillette-Guyonnet S, Cantet C, Vellas B, Nourhashémi F: Living alone with Alzheimer's disease and the risk of adverse outcomes: results from the Plan de Soin et d'Aide dans la maladie d'Alzheimer Study. *Journal of the American Geriatrics Society* 2015, 63(4):651-658. <https://doi.org/10.1111/jgs.13347>

136.* Angel JL, López-Ortega M, Chiu CT, Rote SM, Cantu P, Antequera F, Chen CA: Loss of autonomy: likely dementia and living arrangement transitions among Mexicans and Mexican Americans. *The Gerontologist* 2024, 64(2):gnad083. <https://doi.org/10.1093/geront/gnad083>

137.* Cantu PA, Kim J, López-Ortega M, Rote S, Mejia-Arango S, Angel JL: Living arrangements and dementia among the oldest old: a comparison of Mexicans and Mexican Americans. *Innovation in Aging* 2022, 6(3):igac014. <https://doi.org/10.1093/geroni/igac014>

138.* Rote SM, Angel JL, Kim J, Markides KS: Dual trajectories of dementia and social support in the Mexican-origin population. *The Gerontologist* 2021, 61(3):374-382. <https://doi.org/10.1093/geront/gnaa100>

139.* Balouch S, Rifaat E, Chen HL, Tabet N: Social networks and loneliness in people with Alzheimer's dementia. *International Journal of Geriatric Psychiatry* 2019, 34(5):666-673. <https://doi.org/10.1002/gps.5065>

140.* Bostrom F, Jonsson L, Minthon L, Londos E: Patients with dementia with Lewy bodies have more impaired quality of life than patients with Alzheimer disease. *Alzheimer Disease & Associated Disorders* 2007, 21(2):150-154. <https://doi.org/10.1097/WAD.0b013e318065c4a9>

141.* Brown H, D'Amico F, Knapp M, Orrell M, Rehill A, Vale L, Robinson L: A cost effectiveness analysis of maintenance cognitive stimulation therapy (MCST) for people with dementia: examining the influence of cognitive ability and living arrangements. *Aging & Mental Health* 2019, 23(5):602-607. <https://doi.org/10.1080/13607863.2018.1442410>

142.* Cañabate P, Martínez G, Rosende-Roca M, Moreno M, Preckler S, Valero S, Sotolongo O, Hernández I, Alegret M, Ortega G *et al*: Social representation of dementia: an analysis of 5,792 consecutive cases evaluated in a memory clinic. *Journal of Alzheimer's Disease* 2017, 58(4):1099-1108. <https://doi.org/10.3233/JAD-161119>

143.* Carter L, O'Neill S, Keogh F, Pierce M, O'Shea E: Intensive home care supports, informal care and private provision for people with dementia in Ireland. *Dementia* 2021, 20(1):47-65. <https://doi.org/10.1177/1471301219863580>

144.* Keogh F, Pierce M, Neylon K, Fleming P, Carter L, O'Neill S, O'Shea E: Supporting older people with complex needs at home: report 1: evaluation of the HSE intensive home care package initiative Dublin: Health Service Executive; 2018.

145.* O'Brien I, Smuts K, Fan CW, O'Sullivan M, Warters A: High prevalence of dementia among community dwelling older adults in receipt of state funded home care packages: implications for health care planning. *Irish Journal of Psychological Medicine* 2019, 36(2):139-144. <https://doi.org/10.1017/ipm.2017.80>

146.* Čermáková P, Nelson M, Secnik J, Garcia-Ptacek S, Johnell K, Fastbom J, Kilander L, Winblad B, Eriksdotter M, Religa D: Living alone with Alzheimer's disease: data from SveDem, the Swedish dementia registry. *Journal of Alzheimer's Disease* 2017, 58(4):1265-1272. <https://doi.org/10.3233/jad-170102>

147.* Haaksma ML, Eriksdotter M, Rizzuto D, Leoutsakos J-MS, Olde Rikkert MGM, Melis RJF, Garcia-Ptacek S: Survival time tool to guide care planning in people with dementia. *Neurology* 2020, 94(5):e538-e548. <https://doi.org/10.1212/WNL.0000000000008745>

148.* Lexomboon D, Gavriilidou NN, Höijer J, Skott P, Religa D, Eriksdotter M, Sandborgh-Englund G: Discontinued dental care attendance among people with dementia: a register-based longitudinal study. *Gerodontology* 2021, 38(1):57-65. <https://doi.org/10.1111/ger.12498>

149.* Schwertner E, Zelic R, Secnik J, Johansson B, Winblad B, Eriksdotter M, Religa D: Biting the bullet: firearm ownership in persons with dementia. a registry-based observational study. *Journal of Alzheimer's Disease* 2021, 81(1):179-188. <https://doi.org/10.3233/jad-201365>

150.* Schwertner E: Factors associated with behavioral and psychological symptoms of dementia. Sweden: Karolinska Institutet; 2021.

151.* Charles J, Naglie G, Lee J, Moineddin R, Jaglal S, Tierney MC: Self-report measures of well-being predict incident harm due to self-neglect in cognitively impaired seniors who live alone. *Journal of Alzheimer's Disease* 2015, 44(2):425-430. <https://doi.org/10.3233/jad-141671>

152.* Chen Y-J, Wang W-F, Jhang K-M, Chang M-C, Chang C-C, Liao Y-C: Prediction of institutionalization for patients with dementia in Taiwan according to condition at entry to dementia collaborative care. *Journal of Applied Gerontology* 2022, 41(5):1357-1364. <https://doi.org/10.1177/07334648211073129>

153.* Chi LY, Brayne C, Todd CJ, O'Connor DW, Pollitt PA: Predictors of hospital contact by very elderly people: a pilot study from a cohort of people aged 75 years and over. *Age and Ageing* 1995, 24(5):382-388. <https://doi.org/10.1093/ageing/24.5.382>

154.* Clare L, Martyr A, Henderson C, Gamble L, Matthews FE, Quinn C, Nelis SM, Rusted J, Thom J, Knapp M *et al*: Living alone with mild-to-moderate dementia: findings from the IDEAL cohort. *Journal of Alzheimer's Disease* 2020, 78(3):1207-1216. <https://doi.org/10.3233/jad-200638>

155.* Clare L, Gamble LD, Martyr A, Henderson C, Knapp M, Matthews FE, on behalf of the IDEAL study team: Living alone with mild-to-moderate dementia over a two-year period: longitudinal findings from the IDEAL cohort. *The American Journal of Geriatric Psychiatry* 2024, 32(11):1309-1321. <https://doi.org/10.1016/j.jagp.2024.05.012>

156.* Henderson C, Knapp M, Nelis SM, Quinn C, Martyr A, Wu YT, Jones IR, Victor CR, Pickett JA, Hindle JV *et al*: Use and costs of services and unpaid care for people with mild-to-moderate dementia: baseline results from the IDEAL cohort study. *Alzheimer's & Dementia: Translational Research & Clinical Interventions* 2019, 5(1):685-696. <https://doi.org/10.1016/j.trci.2019.09.012>

157.* Henderson C, Knapp M, Martyr A, Gamble LD, Nelis SM, Quinn C, Pentecost C, Collins R, Wu Y-T, Jones IR *et al*: The use and costs of paid and unpaid care for people with dementia: longitudinal findings from the IDEAL cohort. *Journal of Alzheimer's Disease* 2022, 86(1):135-153. <https://doi.org/10.3233/JAD-215117>

158.* Sabatini S, Martyr A, Gamble LD, Collins R, Matthews FE, Morris RG, Rusted JM, Pentecost C, Quinn C, Clare L: Longitudinal predictors of informant-rated involvement of people with dementia in everyday decision-making: findings from the IDEAL program. *Journal of Applied Gerontology* 2023, 42(2):290-301. <https://doi.org/10.1177/07334648221128558>

159.* Victor CR, Rippon I, Nelis SM, Martyr A, Litherland R, Pickett J, Hart N, Henley J, Matthews F, Clare L: Prevalence and determinants of loneliness in people living with dementia: findings from the IDEAL programme. *International Journal of Geriatric Psychiatry* 2020, 35(8):851-858. <https://doi.org/10.1002/gps.5305>

160.* Conde-Sala JL, Garre-Olmo J, Turró-Garriga O, López-Pousa S, Vilalta-Franch J: Factors related to perceived quality of life in patients with Alzheimer's disease: the patient's perception compared with that of caregivers. *International Journal of Geriatric Psychiatry* 2009, 24(6):585-594. <https://doi.org/10.1002/gps.2161>

161.* Curnow E, Rush R, Gorska S, Forsyth K: Differences in assistive technology installed for people with dementia living at home who have wandering and safety risks. *BMC Geriatrics* 2021, 21(1):613. <https://doi.org/10.1186/s12877-021-02546-7>

162.* Curran JSM: The impact of day care on people with dementia. *International Journal of Geriatric Psychiatry* 1996, 11(9):813-817. <https://doi.org/10.1002/(sici)1099-1166(199609)11:9><813::Aid-gps386>3.0.Co;2-i

163.* Dartigues JF, Avila-Funes JA, Letenneur L, Meillon C, Helmer C, Amieva H, Pérès K: Ten years after the national Alzheimer's plan: dementia remains a hidden syndrome in France. *The Journal of Prevention of Alzheimer's Disease* 2023, 10(3):600-606. <https://doi.org/10.14283/jpad.2023.24>

164.* Diesfeldt H: Psychogeriatric day-care outcome - a 5-year follow-up. *International Journal of Geriatric Psychiatry* 1992, 7(9):673-679. <https://doi.org/10.1002/gps.930070909>

165.* Dramé M, Lang PO, Jolly D, Narbey D, Mahmoudi R, Lanièce I, Somme D, Gauvain JB, Heitz D, Voisin T *et al*: Nursing home admission in elderly subjects with dementia: predictive factors and future challenges. *Journal of the American Medical Directors Association* 2012, 13(1):83.e17-20. <https://doi.org/10.1016/j.jamda.2011.03.002>

166.* Durand M, James A, Ravishankar A, Bamrah JS, Purandare NB: Domiciliary and day care services: why do people with dementia refuse? *Aging & Mental Health* 2009, 13(3):414-419. <https://doi.org/10.1080/13607860902879318>

167.* Ebly EM, Hogan DB, Rockwood K: Living alone with dementia. *Dementia and Geriatric Cognitive Disorders* 1999, 10(6):541-548. <https://doi.org/10.1159/000017202>

168.* Tuokko H, Mac CP, Heath Y: Home alone with dementia. *Aging & Mental Health* 1999, 3(1):21-27. <https://doi.org/10.1080/13607869956406>

169.* Edwards DF, Baum CM: Functional performance of inner city African-American older persons with dementia. *Topics in Geriatric Rehabilitation* 1996, 12(2):17-27.

170.* Edwards DF, Morris JC: Alone and confused: Community-residing older African Americans with dementia. *Dementia* 2007, 6(4):489-506. <https://doi.org/10.1177/1471301207084367>

171.* Eichler T, Hoffmann W, Hertel J, Richter S, Wucherer D, Michalowsky B, Dreier A, Thyrian JR: Living alone with dementia: prevalence, correlates and the utilization of health and nursing care services. *Journal of Alzheimer's Disease* 2016, 52(2):619-629. <https://doi.org/10.3233/jad-151058>

172.* Michalowsky B, Thyrian JR, Eichler T, Hertel J, Wucherer D, Flessa S, Hoffmann W: Economic analysis of formal care, informal care, and productivity losses in primary care patients who screened positive for dementia in Germany. *Journal of Alzheimer's Disease* 2016, 50(1):47-59. <https://doi.org/10.3233/jad-150600>

173.* Michalowsky B, Flessa S, Eichler T, Hertel J, Dreier A, Zwingmann I, Wucherer D, Rau H, Thyrian JR, Hoffmann W: Healthcare utilization and costs in primary care patients with dementia: baseline results of the DelpHi-trial. *The European Journal of Health Economics* 2018, 19(1):87-102. <https://doi.org/10.1007/s10198-017-0869-7>

174.* Michalowsky B, Xie F, Eichler T, Hertel J, Kaczynski A, Kilimann I, Teipel S, Wucherer D, Zwingmann I, Thyrian JR *et al*: Cost-effectiveness of a collaborative dementia care management-Results of a cluster-randomized controlled trial. *Alzheimer's & Dementia* 2019, 15(10):1296-1308. <https://doi.org/10.1016/j.jalz.2019.05.008>

175.* Rädke A, Michalowsky B, Thyrian JR, Eichler T, Xie F, Hoffmann W: Who benefits most from collaborative dementia care from a patient and payer perspective? a subgroup cost-effectiveness analysis. *Journal of Alzheimer's Disease* 2020, 74(2):449-462. <https://doi.org/10.3233/jad-190578>

176.* Thyrian JR, Hertel J, Wucherer D, Eichler T, Michalowsky B, Dreier-Wolfgramm A, Zwingmann I, Kilimann I, Teipel S, Hoffmann W: Effectiveness and safety of dementia care management in primary care: a randomized clinical trial. *JAMA Psychiatry* 2017, 74(10):996-1004. <https://doi.org/10.1001/jamapsychiatry.2017.2124>

177.* Ennis SK, Larson EB, Grothaus L, Helfrich CD, Balch S, Phelan EA: Association of living alone and hospitalization among community-dwelling elders with and without dementia. *Journal of General Internal Medicine* 2014, 29(11):1451-1459. <https://doi.org/10.1007/s11606-014-2904-z>

178.* Farsi A, Casaccia S, Revel GM, Ieee: Assessment of normal and abnormal behaviour of people with dementia in living environment through non-invasive sensors and unsupervised AI. *2022 IEEE International Workshop on Metrology for Living Environment (MetroLivEn)* 2022(1):71-75. <https://doi.org/10.1109/MetroLivEnv54405.2022.9826949>

179.* Ferretti M, Seematter-Bagnoud L, Martin E, Büla CJ: New diagnoses of dementia among older patients admitted to postacute care. *Journal of the American Medical Directors Association* 2010, 11(5):371-376. <https://doi.org/10.1016/j.jamda.2009.10.002>

180.* Fisk AA, Pannill FC: Assessment and care of the community-dwelling Alzheimer's disease patient. *Journal of the American Geriatrics Society* 1987, 35(4):307-311. <https://doi.org/10.1111/j.1532-5415.1987.tb04636.x>

181.* Fowler-Davis S, Barnett D, Kelley J, Curtis D: Potential for digital monitoring to enhance wellbeing at home for people with mild dementia and their family carers. *Journal of Alzheimer's Disease* 2020, 73(3):867-872. <https://doi.org/10.3233/jad-190844>

182.* Fritze F, Ehrt U, Hortobagyi T, Ballard C, Aarsland D: Depressive symptoms in Alzheimer's disease and lewy body dementia: a one-year follow-up study. *Dementia and Geriatric Cognitive Disorders* 2011, 32(2):143-149. <https://doi.org/10.1159/000332016>

183*. Fukatsu T, Kanemoto K: Phantom boarder symptom in elderly Japanese. *Psychogeriatrics* 2022, 22(1):108-112. <https://doi.org/10.1111/psyg.12785>

184.* Gage H, Cheynel J, Williams P, Mitchell K, Stinton C, Katz J, Holland C, Sheehan B: Service utilisation and family support of people with dementia: a cohort study in England. *International Journal of Geriatric Psychiatry* 2015, 30(2):166-177. <https://doi.org/10.1002/gps.4118>

185.* Gallucci M, Spagnolo P, Aricò M, Grossi E: Predictors of response to cholinesterase inhibitors treatment of Alzheimer’s disease: date mining from the TREDEM registry. *Journal of Alzheimer's Disease* 2016, 50(4):969-979. <https://doi.org/10.3233/jad-150747>

186.* Gibson A: Investigating the association of social network and well-being of individuals living alone with cognitive impairment: Ohio State University; 2014.

187.* Gibson AK, Richardson VE: Living alone with cognitive impairment. *American Journal of Alzheimer's Disease & Other Dementias* 2017, 32(1):56-62. <https://doi.org/10.1177/1533317516673154>

188.* Giebel C, Hollinghurst J, Akbari A, Schnier C, Wilkinson T, North L, Gabbay M, Rodgers S: Socio-economic predictors of time to care home admission in people living with dementia in Wales: a routine data linkage study. *International Journal of Geriatric Psychiatry* 2021, 36(4):511-520. <https://doi.org/10.1002/gps.5446>

189.* Gregory S: Memory maintenance groups in the community. *British Journal of Occupational Therapy* 1996, 59(1):25-26. <https://doi.org/10.1177/030802269605900109>

190.* Hamers JP, Bleijlevens MH, Gulpers MJ, Verbeek H: Behind closed doors: involuntary treatment in care of persons with cognitive impairment at home in the Netherlands. *Journal of the American Geriatrics Society* 2016, 64(2):354-358. <https://doi.org/10.1111/jgs.13946>

191.* Han EJ, Lee J, Cho E, Kim H: Socioeconomic costs of dementia based on utilization of health care and long-term-care services: a retrospective cohort study. *International Journal of Environmental Research and Public Health* 2021, 18(2):376. <https://doi.org/10.3390/ijerph18020376>

192.* Hansen ML, Waldorff FB, Waldemar G: Prognostic factors for weight loss over 1-year period in patients recently diagnosed with mild Alzheimer disease. *Alzheimer Disease & Associated Disorders* 2011, 25(3):269-275. <https://doi.org/10.1097/WAD.0b013e3182096624>

193.* Harsányiová M, Prokop P: Living condition, weight loss and cognitive decline among people with dementia. *Nursing Open* 2018, 5(3):275-284. <https://doi.org/10.1002/nop2.137>

194.* Hattori Y, Hiramatsu M, Isowa T, Kitagawa A, Tsujikawa M: The impact of cognitive impairment on disaster preparedness: a cross-sectional study of older adults over the age of 75 requiring special care in Japan. *Journal of Gerontological Social Work* 2022, 65(5):562-579. <https://doi.org/10.1080/01634372.2021.2004568>

195.* Herrick TH: Service utilization among people with cognitive impairment: does living alone make a difference? Boston: University of Massachusetts; 2017.

196.* Houttekier D, Reyniers T, Deliens L, Van den Noortgate N, Cohen J: Dying in hospital with dementia and pneumonia: a nationwide study using death certificate data. *Gerontology* 2014, 60(1):31-37. <https://doi.org/10.1159/000354378>

197.* Hsieh SW, Kim SY, Shim YS, Huang LC, Yang YH: A comparison of sociobehavioral impact on cognitive preservation in Alzheimer's disease between Taiwan and Korea: a cross-national study. *Medicine* 2020, 99(15):e19690. <https://doi.org/10.1097/md.0000000000019690>

198.* Iwasaki M, Motokawa K, Watanabe Y, Hayakawa M, Mikami Y, Shirobe M, Inagaki H, Edahiro A, Ohara Y, Hirano H *et al*: Nutritional status and body composition in cognitively impaired older persons living alone: the Takashimadaira study. *PLoS One* 2021, 16(11):e0260412. <https://doi.org/10.1371/journal.pone.0260412>

199.* Janssen O, Vos SJB, Handels R, Vermunt L, Verheij R, Verhey FRJ, van Hout H, Visser PJ, Joling KJ: Duration of care trajectories in persons with dementia differs according to demographic and clinical characteristics. *Journal of the American Medical Directors Association* 2020, 21(8):1102-1107.e1106. <https://doi.org/10.1016/j.jamda.2020.01.008>

200.* Ju YJ, Nam CM, Lee SG, Park S, Hahm M-I, Park E-C: Evaluation of the South Korean national long-term care insurance-funded cognitive function training programme for older people with mild dementia. *Age and Ageing* 2019, 48(5):636-642. <https://doi.org/10.1093/ageing/afz067>

201.* Kahle-Wrobleski K, Andrews JS, Belger M, Ye W, Gauthier S, Rentz DM, Galasko D: Dependence levels as interim clinical milestones along the continuum of Alzheimer’s disease: 18-month results from the GERAS observational study. *The Journal of Prevention of Alzheimer's Disease* 2017, 4(2):72-80. <https://doi.org/10.14283/jpad.2017.2>

202.* Kamimura T: Older adults with Alzheimer's disease who have used an automatic medication dispenser for 3 or more years. *Clinical Gerontologist* 2019, 42(1):127-133. <https://doi.org/10.1080/07317115.2017.1347594>

203.* Kerpershoek L, de Vugt M, Wolfs C, Orrell M, Woods B, Jelley H, Meyer G, Bieber A, Stephan A, Selbaek G *et al*: Is there equity in initial access to formal dementia care in Europe? The Andersen Model applied to the Actifcare cohort. *International Journal of Geriatric Psychiatry* 2020, 35(1):45-52. <https://doi.org/10.1002/gps.5213>

204.* Kikuchi K, Ijuin M, Awata S, Suzuki T: Exploratory research on outcomes for individuals missing through dementia wandering in Japan. *Geriatrics & Gerontology International* 2019, 19(9):902-906. <https://doi.org/10.1111/ggi.13738>

205.* Kikuchi K, Ooguchi T, Ikeuchi T, Awata S: Exploratory study on the factors related with the early detection of missing older persons with dementia living alone in Japan. *Geriatrics & Gerontology International* 2023, 23(5):362-365. <https://doi.org/10.1111/ggi.14580>

206.* Kisvetrová H, Skoloudík D, Herzig R, Langová K, Kurková P, Tomanová J, Yamada Y: Psychometric properties of the Czech version of the Falls Efficacy Scale-international in patients with early-stage dementia. *Dementia and Geriatric Cognitive Disorders Extra* 2019, 9(2):319-329. <https://doi.org/10.1159/000501676>

207.* Kisvetrová H, Herzig R, Bretšnajdrová M, Tomanová J, Langová K, Školoudík D: Predictors of quality of life and attitude to ageing in older adults with and without dementia. *Aging & Mental Health* 2021, 25(3):535-542. <https://doi.org/10.1080/13607863.2019.1705758>

208.* Kitamura T, Shiota S, Jinkawa S, Kitamura M, Hino S: Effect of preceding home-visit nursing on time to discharge in hospitalization for the treatment of behavioural and psychological symptoms of dementia among patients with limited familial care. *Psychogeriatrics* 2018, 18(1):36-41. <https://doi.org/10.1111/psyg.12282>

209.* Knapp M, Chua KC, Broadbent M, Chang CK, Fernandez JL, Milea D, Romeo R, Lovestone S, Spencer M, Thompson G *et al*: Predictors of care home and hospital admissions and their costs for older people with Alzheimer's disease: findings from a large London case register. *BMJ Open* 2016, 6(11):e013591. <https://doi.org/10.1136/bmjopen-2016-013591>

210.* Lam D, Sewell M, Bell G, Katona C: Who needs psychogeriatric continuing care? *International Journal of Geriatric Psychiatry* 1989, 4(2):109-114. <https://doi.org/10.1002/gps.930040209>

211.* Lampinen J, Conradsson M, Nyqvist F, Olofsson B, Gustafson Y, Nilsson I, Littbrand H: Loneliness among very old people with and without dementia: prevalence and associated factors in a representative sample. *European Journal of Ageing* 2022, 19(4):1441-1453. <https://doi.org/10.1007/s10433-022-00729-8>

212.* Lehmann SW, Black BS, Shore A, Kasper J, Rabins PV: Living alone with dementia: lack of awareness adds to functional and cognitive vulnerabilities. *International Psychogeriatrics* 2010, 22(5):778-784. <https://doi.org/10.1017/s1041610209991529>

213.* Lin HR, Otsubo T, Imanaka Y: Survival analysis of increases in care needs associated with dementia and living alone among older long-term care service users in Japan. *BMC Geriatrics* 2017, 17(1):182. <https://doi.org/10.1186/s12877-017-0555-8>

214.* Lin Z, Qian Y, Gill TM, Hou X, Chen S, Chen X: Absence of care among community-living older persons with dementia and functional limitations: a cross-national analysis of population survey from 22 countries. *medRxiv* 2023. <https://doi.org/10.1101/2023.10.05.23296622>

215.* Li C, Jin S, Cao X, Han L, Sun N, Allore H, Hoogendijk EO, Xu X, Feng Q, Liu X *et al*: Catastrophic health expenditure among Chinese adults living alone with cognitive impairment: findings from the CHARLS. *BMC Geriatrics* 2022, 22(1):640. <https://doi.org/10.1186/s12877-022-03341-8>

216.* White L, Ingraham B, Larson E, Fishman P, Park S, Coe NB: Observational study of patient characteristics associated with a timely diagnosis of dementia and mild cognitive impairment without dementia. *Journal of General Internal Medicine* 2022, 37(12):2957-2965. <https://doi.org/10.1007/s11606-021-07169-7>

217.* Zuo D, Heflin CM: Cognitive impairment and supplemental nutrition assistance program take-up among the eligible older Americans. *The Journals of Gerontology, Series B: Psychological Sciences and Social Sciences* 2023, 78(1):99-110. <https://doi.org/10.1093/geronb/gbac111>

218.* Lofthouse-Jones C, King P, Pocock H, Ramsay M, Jadzinski P, England E, Taylor S, Cavalier J, Fogg C: Reducing ambulance conveyance for older people with and without dementia: evidence of the role of social care from a regional, year-long service evaluation using retrospective routine data. *British Paramedic Journal* 2021, 6(3):58-69. <https://doi.org/10.29045/14784726.2021.12.6.3.58>

219.* LoGiudice D, Hassett A, Cook R, Flicker L, Ames D: Equity of access to a memory clinic in Melbourne? Non-English speaking background attenders are more severely demented and have increased rates of psychiatric disorders. *International Journal of Geriatric Psychiatry* 2001, 16(3):327-334. <https://doi.org/10.1002/gps.346>

220.* Manivannan M, Heunis J, Hooper SM, Bernstein Sideman A, Lui KP, Braley TL, Possin KL, Chiong W: Use of telephone- and internet-based support to elicit and address financial abuse and mismanagement in dementia: experiences from the care ecosystem study. *Journal of Alzheimer's Disease* 2022, 86(1):219-229. <https://doi.org/10.3233/jad-215284>

221.* Måvall L, Malmberg B: Day care for persons with dementia: an alternative for whom? *Dementia* 2007, 6(1):27-43. <https://doi.org/10.1177/1471301207075627>

222.* Meaney AM, Croke M, Kirby M: Needs assessment in dementia. *International Journal of Geriatric Psychiatry* 2005, 20(4):322-329. <https://doi.org/10.1002/gps.1284>

223.* Mengelers A, Moermans VRA, Bleijlevens MHC, Verbeek H, Capezuti E, Tan F, Milisen K, Hamers JPH: Involuntary treatment in dementia care at home: results from the Netherlands and Belgium. *Journal of Clinical Nursing* 2022, 31(13-14):1998-2007. <https://doi.org/10.1111/jocn.15457>

224.* Mets T, De Deyn PP, Pals P, De Lepeleire J, Vandewoude M, Ventura M, Ivanoiu A, Albert A, Seghers AK, Grp C: COGNOS: care for people with cognitive dysfunction: a national observational study. *Alzheimer Disease & Associated Disorders* 2013, 27(2):123-132. <https://doi.org/10.1097/WAD.0b013e318256d1d0>

225.* Michelet M, Lund A, Strand BH, Engedal K, Selbaek G, Bergh S: Characteristics of patients assessed for cognitive decline in primary healthcare, compared to patients assessed in specialist healthcare. *Scandinavian Journal of Primary Health Care* 2020, 38(2):107-116. <https://doi.org/10.1080/02813432.2020.1753334>

226.* Miranda-Castillo C, Woods B, Orrell M: People with dementia living alone: what are their needs and what kind of support are they receiving? *International Psychogeriatrics* 2010, 22(4):607-617. <https://doi.org/10.1017/s104161021000013x>

227.* Miyawaki CE, McClellan A, Bouldin ED, Brohard C, Spencer H, Tahija N, Kunik ME: Feasibility and efficacy of life review delivered by virtually-trained family caregivers. *Journal of Alzheimer's Disease* 2023, 95(2):573-583. <https://doi.org/10.3233/jad-230371>

228.* Mjørud M, Selbæk G, Bjertness E, Edwin TH, Engedal K, Knapskog AB, Strand BH: Time from dementia diagnosis to nursing-home admission and death among persons with dementia: a multistate survival analysis. *PLoS One* 2020, 15(12):e0243513. <https://doi.org/10.1371/journal.pone.0243513>

229.* Moholt JM, Friborg O, Blix BH, Henriksen N: Factors affecting the use of home-based services and out-of-home respite care services: a survey of family caregivers for older persons with dementia in Northern Norway. *Dementia* 2020, 19(5):1712-1731. <https://doi.org/10.1177/1471301218804981>

230.* Montastruc F, Gardette V, Cantet C, Piau A, Lapeyre-Mestre M, Vellas B, Montastruc J-L, Andrieu S, Group RF: Potentially inappropriate medication use among patients with Alzheimer disease in the REAL.FR cohort: be aware of atropinic and benzodiazepine drugs! *European Journal of Clinical Pharmacology* 2013, 69(8):1589-1597. <https://doi.org/10.1007/s00228-013-1506-8>

231.* Nourhashemi F, Amouyal-Barkate K, Gillette-Guyonnet S, Cantet C, Vellas B: Living alone with Alzheimer's disease: cross-sectional and longitudinal analysis in the REAL.FR Study. *The Journal of Nutrition, Health and Aging* 2005, 9(2):117-120.

232.* Tavassoli N, Perrin A, Bérard E, Gillette S, Vellas B, Rolland Y: Factors associated with undertreatment of atrial fibrillation in geriatric outpatients with Alzheimer disease. *American Journal of Cardiovascular Drugs* 2013, 13(6):425-433. <https://doi.org/10.1007/s40256-013-0040-5>

233.* Morris PA: A survey of 100 female senile admissions to a mental hospital. *Journal of Mental Science* 1962, 108(457):801-803. <https://doi.org/10.1192/bjp.108.457.801>

234.* Morycz RK: Caregiving strain and the desire to institutionalize family members with Alzheimer's disease: possible predictors and model development. *Research on Aging* 1985, 7(3):329-361. <https://doi.org/10.1177/0164027585007003002>

235.* Murayama N, Iseki E, Endo T, Nagashima K, Yamamoto R, Ichimiya Y, Arai H, Sato K: Risk factors for delusion of theft in patients with Alzheimer's disease showing mild dementia in Japan. *Aging & Mental Health* 2009, 13(4):563-568. <https://doi.org/10.1080/13607860902774402>

236.* Nakanishi M, Igarashi A, Ueda K, Brnabic AJM, Treuer T, Sato M, Kahle-Wrobleski K, Meguro K, Yamada M, Mimura M *et al*: Costs and resource use associated with community-dwelling patients with Alzheimer’s disease in Japan: baseline results from the prospective observational GERAS-J study. *Journal of Alzheimer's Disease* 2020, 74(1):127-138. <https://doi.org/10.3233/jad-190811>

237.* Nguyen AN, Nguyen BT, Nguyen BT, Nguyen BTT, Nguyen NTA, Dang NTK, Nguyen ATP, Nguyen AT, Pham T, Mantyh WG *et al*: Care needs assessment of older adults with dementia in a semi-rural district in Vietnam: a community-based cross-sectional study. *Psychogeriatrics* 2024, 24(2):249-258. <https://doi.org/10.1111/psyg.13068>

238.* Nicholas LH, Langa KM, Bynum JPW, Hsu JW: Financial presentation of Alzheimer disease and related dementias. *JAMA Internal Medicine* 2021, 181(2):220-227. <https://doi.org/10.1001/jamainternmed.2020.6432>

239.* Nygård L, Starkhammar S, Lilja M: The provision of stove timers to individuals with cognitive impairment. *Scandinavian Journal of Occupational Therapy* 2008, 15(1):4-12. <https://doi.org/10.1080/11038120601124240>

240.* O'Connor DW, Pollitt PA, Brook CP, Reiss BB: The distribution of services to demented elderly people living in the community. *International Journal of Geriatric Psychiatry* 1989, 4(6):339-344. <https://doi.org/10.1002/gps.930040607>

241.* O'Connor DW, Pollitt PA, Brook CP, Reiss BB, Roth M: Does early intervention reduce the number of elderly people with dementia admitted to institutions for long term care? *BMJ* 1991, 302(6781):871-875. <https://doi.org/10.1136/bmj.302.6781.871>

242.* Okamura T, Ura C, Sugiyama M, Inagaki H, Miyamae F, Edahiro A, Taga T, Tsuda S, Nakayama R, Ito K *et al*: Factors associated with inability to attend a follow-up assessment, mortality, and institutionalization among community-dwelling older people with cognitive impairment during a 5-year period: evidence from community-based participatory research. *Psychogeriatrics* 2022, 22(3):332-342. <https://doi.org/10.1111/psyg.12816>

243.* Pentzek M, Wollny A, Wiese B, Jessen F, Haller F, Maier W, Riedel-Heller SG, Angermeyer MC, Bickel H, Mosch E *et al*: Apart from nihilism and stigma: what influences general practitioners' accuracy in identifying incident dementia. *The American Journal of Geriatric Psychiatry* 2009, 17(11):965-975. <https://doi.org/10.1097/JGP.0b013e3181b2075e>

244.* Pitkala KH, Laakkonen M-L, Kallio E-L, Kautiainen H, Raivio MM, Tilvis RS, Strandberg TE, Ohman H: Monetary value of informal caregiving in dementia from a societal perspective. *Age and Ageing* 2021, 50(3):861-867. <https://doi.org/10.1093/ageing/afaa196>

245.* Pongan E, Dorey J-M, Krolak-Salmon P, Federico D, Sellier C, Auguste N, Fabre F, Laurent B, Trombert-Paviot B, Rouch I: Predictors of discharge destinations and three-month evolution of patients initially hospitalized in a cognitive behavioral unit. *Journal of Alzheimer's Disease* 2017, 60(4):1259-1266. <https://doi.org/10.3233/JAD-170419>

246.* Prescop KL, Dodge HH, Morycz RK: Elders with dementia living in the community with and without caregivers. *International Psychogeriatrics* 1999, 11(3):235-250. <https://doi.org/10.1017/S1041610299005803>

247.* Puaschitz NG, Jacobsen FF, Mannseth J, Angeles RC, Berge LI, Gedde MH, Husebo BS: Factors associated with access to assistive technology and telecare in home-dwelling people with dementia: baseline data from the LIVE@Home.Path trial. *BMC Medical Informatics and Decision Making* 2021, 21(1):264. <https://doi.org/10.1186/s12911-021-01627-2>

248.* Puaschitz NGS, Jacobsen FF, Berge LI, Husebo BS: Access to, use of, and experiences with social alarms in home-living people with dementia: results from the LIVE@Home.Path trial. *Frontiers in Aging Neuroscience* 2023, 15(1):1167616. <https://doi.org/10.3389/fnagi.2023.1167616>

249.* Regan C, Katona C, Walker Z, Livingston G: Relationship of exercise and other risk factors to depression of Alzheimer's disease: the LASER-AD study. *International Journal of Geriatric Psychiatry* 2005, 20(3):261-268. <https://doi.org/10.1002/gps.1278>

250.* Rhee Y, Csernansky JG, Emanuel LL, Chang C-G, Shega JW: Psychotropic medication burden and factors associated with antipsychotic use: an analysis of a population-based sample of community-dwelling older persons with dementia. *Journal of the American Geriatrics Society* 2011, 59(11):2100-2107. <https://doi.org/10.1111/j.1532-5415.2011.03660.x>

251.* Risvoll H, Giverhaug T, Halvorsen KH, Waaseth M, Musial F: Direct and indirect risk associated with the use of dietary supplements among persons with dementia in a Norwegian memory clinic. *BMC Complementary Medicine and Therapies* 2017, 17(1):261. <https://doi.org/10.1186/s12906-017-1765-5>

252.* Rokstad AMM, Engedal K, Kirkevold O, Benth JS, Selbæk G: The impact of attending day care designed for home-dwelling people with dementia on nursing home admission: a 24-month controlled study. *BMC Health Services Research* 2018, 18(1):864. <https://doi.org/10.1186/s12913-018-3686-5>

253.* Rongve A, Vossius C, Nore S, Testad I, Aarsland D: Time until nursing home admission in people with mild dementia: comparison of dementia with Lewy bodies and Alzheimer's dementia. *International Journal of Geriatric Psychiatry* 2014, 29(4):392-398. <https://doi.org/10.1002/gps.4015>

254.* Rowe MA, Greenblum CA, Boltz M, Galvin JE: Missing drivers with dementia: antecedents and recovery. *Journal of the American Geriatrics Society* 2012, 60(11):2063-2069. <https://doi.org/10.1111/j.1532-5415.2012.04159.x>

255.* Rozzini L, Cornali C, Chilovi BV, Ghianda D, Padovani A, Trabucchi M: Predictors of institutionalization in demented patients discharged from a rehabilitation unit. *Journal of the American Medical Directors Association* 2006, 7(6):345-349. <https://doi.org/10.1016/j.jamda.2005.11.008>

256.* Salva A, Andrieu S, Fernandez E, Schiffrin EJ, Moulin J, Decarli B, Guigoz Y, Vellas B, Espinosa C, Fort I *et al*: Health and nutritional promotion program for patients with dementia (nutrialz study): design and baseline data. *Journal of Nutrition, Health and Aging* 2009, 13(6):529-537. <https://doi.org/10.1007/s12603-009-0103-0>

257.* Sandberg L, Nilsson I, Rosenberg L: Home care services for older clients with and without cognitive impairment in Sweden. *Health and Social Care in the Community* 2019, 27(1):139-150. <https://doi.org/10.1111/hsc.12631>

258.* Sandberg L: Living at home with cognitive impairments: risks during daily living and support from home care service. Sweden: Karolinska Institutet; 2018.

259.* Schneider J, Hallam A, Murray J, Foley B, Atkin L, Banerjee S, Islam MK, Mann A: Formal and informal care for people with dementia: factors associated with service receipt. *Aging & Mental Health* 2002, 6(3):255-265. <https://doi.org/10.1080/13607860220142486>

260.* Shinagawa S, Yatabe Y, Hashimoto M, Nakayama K, Ikeda M: A comparison of family care infrastructure for demented elderly in inner cities and regional areas in Japan. *Psychogeriatrics* 2012, 12(3):159-164. <https://doi.org/10.1111/j.1479-8301.2011.00395.x>

261.* Sm-Rahman A, Hydén LC, Kelfve S: Eldercare services for people with and without a dementia diagnosis: an analysis of Swedish registry data. *BMC Health Services Research* 2021, 21(1):893. <https://doi.org/10.1186/s12913-021-06891-6>

262.* Sm-Rahman A, Meinow B, Hyden LC, Kelfve S: Patterns of long-term care utilization during the last five years of life among Swedish older adults with and without dementia. *PLoS One* 2023, 18(10):e0286930. <https://doi.org/10.1371/journal.pone.0286930>

263.* Souza AM: Early stage memory loss interventions: utilization, impact and the experience of living alone. Washington: University of Washington; 2017.

264.* Swanwick GR, Coen RF, Maguire CP, Kirby M, Walsh JB, O'Neill D, Coakley D, Lawlor BA: The association between demographic factors, disease severity and the duration of symptoms at clinical presentation in elderly people with dementia. *Age and Ageing* 1999, 28(3):295-299. <https://doi.org/10.1093/ageing/28.3.295>

265.* Thoits T, Sadasivan J, Parker JL, Andersen NJ: Acute healthcare utilization of a multidisciplinary neurocognitive dementia patient cohort. *Journal of Clinical Neurology* 2020, 16(3):433-437. <https://doi.org/10.3988/jcn.2020.16.3.433>

266.* Thiruchselvam T, Naglie G, Moineddin R, Charles J, Orlando L, Jaglal S, Snow W, Tierney MC: Risk factors for medication nonadherence in older adults with cognitive impairment who live alone. *International Journal of Geriatric Psychiatry* 2012, 27(12):1275-1282. <https://doi.org/10.1002/gps.3778>

267.* Tierney MC, Charles J, Jaglal S, Snow WG, Szalai JP, Spizzirri F, Fisher RH: Identification of those at greatest risk of harm among cognitively impaired people who live alone. *Aging Neuropsychology and Cognition* 2001, 8(3):182-191. <https://doi.org/10.1076/anec.8.3.182.829>

268.* Tierney MC, Charles J, Naglie G, Jaglal S, Kiss A, Fisher RH: Risk factors for harm in cognitively impaired seniors who live alone: a prospective study. *Journal of the American Geriatrics Society* 2004, 52(9):1435-1441. <https://doi.org/10.1111/j.0002-8614.2004.52404.x>

269.* Tierney MC, Snow WG, Charles J, Moineddin R, Kiss A: Neuropsychological predictors of self-neglect in cognitively impaired older people who live alone. *The American Journal of Geriatric Psychiatry* 2007, 15(2):140-148. <https://doi.org/10.1097/01.JGP.0000230661.32735.c0>

270.* Tochimoto S, Kitamura M, Hino S, Kitamura T: Predictors of home discharge among patients hospitalized for behavioural and psychological symptoms of dementia. *Psychogeriatrics* 2015, 15(4):248-254. <https://doi.org/10.1111/psyg.12114>

271.* Tsai CF, Huang MH, Cheng CM, Lee JJ, Wang WF, Huang LC, Huang LK, Lee WJ, Sung PS, Liu YC *et al*: Determinants of long-term care service use by persons with dementia: a national dementia registry study conducted in Taiwan. *International Journal of Geriatric Psychiatry* 2022, 37(5). <https://doi.org/10.1002/gps.5719>

272.* Ura C, Okamura T, Sugiyama M, Miyamae F, Yamashita M, Nakayama R, Edahiro A, Taga T, Inagaki H, Ogawa M *et al*: Living on the edge of the community: factors associated with discontinuation of community living among people with cognitive impairment. *BMC Geriatrics* 2021, 21(1):131. <https://doi.org/10.1186/s12877-021-02084-2>

273.* Villars H, Dupuy C, Soler P: A follow-up intervention in severely demented patients after discharge from a special Alzheimer acute care unit. *International Journal of Geriatric Psychiatry* 2013, 28(11):1131-1140. <https://doi.org/10.1002/gps.3932>

274.* Vislapuu M, Angeles RC, Berge LI, Kjerstad E, Gedde MH, Husebo BS: The consequences of COVID-19 lockdown for formal and informal resource utilization among home-dwelling people with dementia: results from the prospective PAN.DEM study. *BMC Health Services Research* 2021, 21(1):1003. <https://doi.org/10.1186/s12913-021-07041-8>

275.* Wang J, Shen JY, Conwell Y, Yu F, Nathan K, Heffner KL, Li Y, Caprio TV: Antipsychotic use among older patients with dementia receiving home health care services: prevalence, predictors, and outcomes. *Journal of the American Geriatrics Society* 2023, 71(12):3768-3779. <https://doi.org/10.1111/jgs.18555>

276.* Wang W-F, Chiu P-Y, Lin Y-T, Hu C-J, Fuh J-L, Yang Y-H: Registration of Alzheimer’s disease in Taiwan: patient and informant. *American Journal of Alzheimer's Disease & Other Dementias* 2013, 29(1):18-22. <https://doi.org/10.1177/1533317513504818>

277.* Washida K, Kitajima E, Tanaka T, Ikeda S, Chiba T, Noda K, Yoshimoto T, Fukuma K, Saito S, Ihara M: A nationwide multi-center questionnaire survey on the real-world state and clinical management of poststroke dementia in Japan. *Journal of Alzheimer's Disease* 2021, 84(3):1103-1114. <https://doi.org/10.3233/jad-215006>

278.* Watari KF, Gatz M: Pathways to care for Alzheimer's disease among Korean Americans. *Cultural Diversity & Ethnic Minority Psychology* 2004, 10(1):23-38. <https://doi.org/10.1037/1099-9809.10.1.23>

279.* Watari K, Wetherell JL, Gatz M, Delaney J, Ladd C, Cherry D: Long distance caregivers: characteristics, service needs, and use of a long distance caregiver program. *Clinical Gerontologist* 2006, 29(4):61-77. <https://doi.org/10.1300/J018v29n04_05>

280.* Wattmo C, Wallin AK, Londos E, Minthon L: Risk factors for nursing home placement in Alzheimer's disease: a longitudinal study of cognition, ADL, service utilization, and cholinesterase inhibitor treatment. *The Gerontologist* 2011, 51(1):17-27. <https://doi.org/10.1093/geront/gnq050>

281.* Wattmo C, Londos E, Minthon L: Solitary living in Alzheimer's disease over 3 years: association between cognitive and functional impairment and community-based services. *Clinical Interventions in Aging* 2014, 9(1):1951-1962. <https://doi.org/10.2147/cia.S71709>

282.* Webber PA, Fox P, Burnette D: Living alone with Alzheimer's disease: effects on health and social service utilization patterns. *The Gerontologist* 1994, 34(1):8-14. <https://doi.org/10.1093/geront/34.1.8>

283.* Wenborn J, O'Keeffe AG, Mountain G, Moniz-Cook E, King M, Omar RZ, Mundy J, Burgess J, Poland F, Morris S *et al*: Community Occupational Therapy for people with dementia and family carers (COTiD-UK) versus treatment as usual (Valuing Active Life in Dementia VALID ) study: a single-blind, randomised controlled trial. *Plos Medicine* 2021, 18(1):e1003433. <https://doi.org/10.1371/journal.pmed.1003433>

284.* Wenger GC: Dementia sufferers living at home. *International Journal of Geriatric Psychiatry* 1994, 9(9):721-733. <https://doi.org/10.1002/gps.930090907>

285.* Wilkins CH, Wilkins KL, Meisel M, Depke M, Williams J, Edwards DF: Dementia undiagnosed in poor older adults with functional impairment. *Journal of the American Geriatrics Society* 2007, 55(11):1771-1776. <https://doi.org/10.1111/j.1532-5415.2007.01417.x>

286.* Wong JFW, Kirk A, Perlett L, Karunanayake C, Morgan D, O'Connell M E: Characteristics of young-onset and late-onset dementia patients at a remote memory clinic. *Canadian Journal of Neurological Sciences* 2020, 47(3):320-327. <https://doi.org/10.1017/cjn.2020.8>

287.* Woods JP, Phanjoo AL: A follow-up study of psychogeriatric day hospital patients with dementia. *International Journal of Geriatric Psychiatry* 1991, 6(3):183-188. <https://doi.org/10.1016/j.yebeh.2010.10.029>

288.* Wübbeler M, Wucherer D, Hertel J, Michalowsky B, Heinrich S, Meyer S, Schaefer-Walkmann S, Hoffmann W, Thyrian JR: Antidementia drug treatment in dementia networks in Germany: use rates and factors associated with treatment use. *BMC Health Services Research* 2015, 15(1):205. <https://doi.org/10.1186/s12913-015-0855-7>

289.* Yaffe K, Fox P, Newcomer R, Sands L, Lindquist K, Dane K, Covinsky KE: Patient and caregiver characteristics and nursing home placement in patients with dementia. *JAMA* 2002, 287(16):2090-2097. <https://doi.org/10.1001/jama.287.16.2090>

290.* Zafeiridi E, McMichael AJ, Passmore AP, McGuinness B: Living alone for people on dementia medication: related use of drugs. *Aging* 2020, 12(20):20924-20929. <https://doi.org/10.18632/aging.104125>

291.* Zhao H, Novella J-L, Dramé M, Mahmoudi R, Barbe C, di Pollina L, Aquino J-P, Pfitzenmeyer P, Rouaud O, George M-Y *et al*: Factors associated with caregivers' underestimation of quality of life in patients with Alzheimer’s disease. *Dementia and Geriatric Cognitive Disorders* 2012, 33(1):11-17. <https://doi.org/10.1159/000333070>

292.* Andrew J, Wilkinson H, Prior S: 'Guid times wi the bad times': the meanings and experiences of befriending for people living alone with dementia. *Dementia* 2022, 21(1):21-40. <https://doi.org/10.1177/14713012211024488>

293.* Baruch J, Downs M, Baldwin C, Bruce E: A case study in the use of technology to reassure and support a person with dementia. *Dementia* 2004, 3(3):372-377. <https://doi.org/10.1177/1471301204045192>

294.* Cott CA, Tierney MC: Acceptable and unacceptable risk: balancing everyday risk by family members of older cognitively impaired adults who live alone. *Health Risk & Society* 2013, 15(5):402-415. <https://doi.org/10.1080/13698575.2013.801936>

295.* de Witt L, Ploeg J, Black M: Living on the threshold: the spatial experience of living alone with dementia. *Dementia* 2009, 8(2):263-291. <https://doi.org/10.1177/1471301209103273>

296.* de Witt L, Ploeg J, Black M: Living alone with dementia: an interpretive phenomenological study with older women. *Journal of Advanced Nursing* 2010, 66(8):1698-1707. <https://doi.org/10.1111/j.1365-2648.2010.05295.x>

297.* de Witt L, Ploeg J: Caring for older people living alone with dementia: healthcare professionals' experiences. *Dementia* 2016, 15(2):221-238. <https://doi.org/10.1177/1471301214523280>

298.* Duane F, Brasher K, Koch S: Living alone with dementia. *Dementia* 2013, 12(1):123-136. <https://doi.org/10.1177/1471301211420331>

299.* Evans N, Carey-Smith B, Orpwood R: Using smart technology in an enabling way: a review of using technology to support daily life for a tenant with moderate dementia. *British Journal of Occupational Therapy* 2011, 74(5):249-253. <https://doi.org/10.4276/030802211x13046730116614>

300.* Evans D, Price K, Meyer J: Home alone with dementia. *Sage Open* 2016, 6(3). <https://doi.org/10.1177/2158244016664954>

301.* Frazer SM, Oyebode JR, Cleary A: How older women who live alone with dementia make sense of their experiences: an interpretative phenomenological analysis. *Dementia* 2012, 11(5):677-693. <https://doi.org/10.1177/1471301211419018>

302.* Gethin-Jones S: Familial perceptions of the impact of outcome-focused homecare with older people experiencing dementia and living alone. *Working with Older People: Community Care Policy & Practice* 2014, 18(2):90-96. <https://doi.org/10.1108/WWOP-12-2013-0031>

303.* Gilmour H, Gibson F, Campbell J: Living alone with dementia: a case study approach to understanding risk. *Dementia* 2003, 2(3):403-420. <https://doi.org/10.1177/14713012030023008>

304.* Groen-van de Ven L, Smits C, Oldewarris K: Decision trajectories in dementia care networks. *Research on Aging* 2017, 39(9):1039-1071. <https://doi.org/10.1177/01640275166567>

305.* Harris PB: The experience of living alone with early stage Alzheimer's disease: what are the person's concerns? *Alzheimer's Care Today* 2006, 7(2):84-94.

306.* Heaton J, Martyr A, Nelis SM, Marková IS, Morris RG, Roth I, Woods RT, Clare L: Future outlook of people living alone with early-stage dementia and their non-resident relatives and friends who support them. *Ageing & Society* 2021, 41(11):2660-2680. <https://doi.org/10.1017/s0144686x20000513>

307.* Illiger K, Walter U, Koppelin F: "I can do that on my own!" On the relevance of subjective factors to the use of formal support, from the perspective of people with dementia. *Health Care for Women International* 2021, 42(11):1298-1320. <https://doi.org/10.1080/07399332.2021.1876064>

308.* Johannessen A, Engedal K, Haugen PK, Dourado MCN, Thorsen K: "To be, or not to be": experiencing deterioration among people with young-onset dementia living alone. *International Journal of Qualitative Studies on Health and Well-being* 2018, 13(1):1490620. <https://doi.org/10.1080/17482631.2018.1490620>

309.* Johannessen A, Engedal K, Haugen PK, Dourado MC, Thorsen K: Coping with transitions in life: a four-year longitudinal narrative study of single younger people with dementia. *Journal of Multidisciplinary Healthcare* 2019, 12:479-492. <https://doi.org/10.2147/jmdh.S208424>

310.* Thorsen K, Dourado MCN, Johannessen A: Developing dementia: the existential experience of the quality of life with young-onset dementia - a longitudinal case study. *Dementia* 2020, 19(3):878-893. <https://doi.org/10.1177/1471301218789990>

311.* Kitamura T, Tanimoto C, Oe S, Kitamura M, Hino S: Familial caregivers' experiences with home-visit nursing for persons with dementia who live alone. *Psychogeriatrics* 2019, 19(1):3-9. <https://doi.org/10.1111/psyg.12352>

312.* Knight C, Harrison Dening K: Management of long-term conditions and dementia: the role of the Admiral Nurse. *British Journal of Community Nursing* 2017, 22(6):295-302. <https://doi.org/10.12968/bjcn.2017.22.6.295>

313.* Kuhn D, Moss L: Preserving autonomy and selfhood in Alzheimer's disease: a case study. *Journal of Social Work in Long-Term Care* 2002, 1(3):17-35. <https://doi.org/10.1300/J181v01n03_06>

314.* Lloyd BT, Stirling C: The will to mobility: life-space satisfaction and distress in people with dementia who live alone. *Ageing & Society* 2015, 35(9):1801-1820. <https://doi.org/10.1017/s0144686x14000683>

315.* Lussier M, Aboujaoudé A, Couture M, Moreau M, Laliberté C, Giroux S, Pigot H, Gaboury S, Bouchard K, Belchior P *et al*: Using ambient assisted living to monitor older adults with Alzheimer disease: single-case study to validate the monitoring report. *JMIR Medical Informatics* 2020, 8(11):e20215. <https://doi.org/10.2196/20215>

316.* Mizuno J, Sadohara K, Nihei M, Onaka S, Nishiura Y, Inoue T: The application of an information support robot to reduce agitation in an older adult with Alzheimer's disease living alone in a community dwelling: a case study. *Hong Kong Journal of Occupational Therapy* 2021, 34(1):50-59. <https://doi.org/10.1177/15691861211005059>

317.* Nygård L, Starkhammar S: Telephone use among noninstitutionalized persons with dementia living alone: mapping out difficulties and response strategies. *Scandinavian Journal of Caring Sciences* 2003, 17(3):239-249. <https://doi.org/10.1046/j.1471-6712.2003.00177.x>

318.* Nygård L, Starkhammar S: The use of everyday technology by people with dementia living alone: mapping out the difficulties. *Aging & Mental Health* 2007, 11(2):144-155. <https://doi.org/10.1080/13607860600844168>

319.* Odzakovic E, Hellström I, Ward R, Kullberg A: 'Overjoyed that I can go outside': using walking interviews to learn about the lived experience and meaning of neighbourhood for people living with dementia. *Dementia* 2020, 19(7):2199-2219. <https://doi.org/10.1177/1471301218817453>

320.* Odzakovic E, Kullberg A, Hellström I, Clark A, Campbell S, Manji K, Rummery K, Keady J, Ward R: 'It's our pleasure, we count cars here': an exploration of the 'neighbourhood-based connections' for people living alone with dementia. *Ageing & Society* 2021, 41(3):645-670. <https://doi.org/10.1017/s0144686x19001259>

321.* Price C: Monitoring people with dementia ‐ controlling or liberating? *Quality in Ageing* 2007, 8(3). <https://doi.org/10.1108/14717794200700020>

322.* Quail Z, Carter MM, Wei A, Li XL: Management of cognitive decline in Alzheimer's disease using a non-pharmacological intervention program A case report. *Medicine* 2020, 99(21):e20128. <https://doi.org/10.1097/md.0000000000020128>

323.* Smith GE, Lunde AM, Hathaway JC, Vickers KS: Telehealth home monitoring of solitary persons with mild dementia. *American Journal of Alzheimer's Disease & Other Dementias* 2007, 22(1):20-26. <https://doi.org/10.1177/1533317506295888>

324.* Suwa S, Otani S, Tsujimura M, Nogawa K, Shiya Y: The diary of a nonagenarian-centenarian woman with dementia: memory loss, life changes, and community care in Japan. *International Journal of Nursing Practice* 2018, 24 Suppl 1:e12655. <https://doi.org/10.1111/ijn.12655>

325.* Svanström R, Sundler AJ: Gradually losing one's foothold--a fragmented existence when living alone with dementia. *Dementia* 2015, 14(2):145-163. <https://doi.org/10.1177/1471301213494510>

326.* Waugh F: Where does risk feature in community care practice with older people with dementia who live alone? *Dementia* 2009, 8(2):205-222. <https://doi.org/10.1177/147130120910325>

327.* Zwierenberg E, Herman H, Lukkien D, Cornelisse L, Finnema E, Dijkstra A, Hagedoorn M, Sanderman R: A lifestyle monitoring system to support (in)formal caregivers of people with dementia: analysis of users need, benefits, and concerns. *Gerontechnology* 2018, 17(4):194-205. <https://doi.org/10.4017/gt.2018.17.4.001.00>
